# Polygenic risk scores for schizophrenia and major depression are associated with socio-economic indicators of adversity in two British community samples

**DOI:** 10.1038/s41398-022-02247-8

**Published:** 2022-11-14

**Authors:** Sandra Machlitt-Northen, Robert Keers, Patricia B. Munroe, David M. Howard, Michael Pluess

**Affiliations:** 1 Department of Biological and Experimental Psychology, Queen Mary University of London, London, UK; 2 Department of Clinical Pharmacology, William Harvey Research Institute, Queen Mary University of London, Charterhouse Square, London, UK; 3 Social, Genetic and Developmental Psychiatry Centre, King’s College London, London, UK; 4 Division of Psychiatry, University of Edinburgh, Royal Edinburgh Hospital, Edinburgh, UK

**Keywords:** Psychology, Genetics

## Abstract

Schizophrenia (SCZ) and major depressive disorder (MDD) are complex psychiatric disorders which contribute substantially to the global burden of disease. Both psychopathologies are heritable with some genetic overlap between them. Importantly, SCZ and MDD have also been found to be associated with environmental risk factors. However, rather than being independent of genetic influences, exposure to environmental risk factors may be under genetic control, known as gene-environment correlation (rGE). In this study we investigated rGE in relation to polygenic risk scores for SCZ and MDD in adults, derived from large genome-wide association studies, across two different British community samples: Understanding Society (USoc) and the National Child Development Study (NCDS). We tested whether established environmental risk factors for SCZ and/or MDD are correlated with polygenic scores in adults and whether these associations differ between the two disorders and cohorts. Findings partially overlapped between disorders and cohorts. In NCDS, we identified a significant correlation between the genetic risk for MDD and an indicator of low socio-economic status, but no significant findings emerged for SCZ. In USoc, we replicated associations between indicators of low socio-economic status and the genetic propensity for MDD. In addition, we identified associations between the genetic susceptibility for SCZ and being single or divorced. Results across both studies provide further evidence that the genetic risk for SCZ and MDD were associated with common environmental risk factors, specifically MDD’s association with lower socio-economic status.

## Introduction

Schizophrenia (SCZ) and major depressive disorder (MDD) are severe psychiatric disorders with a lifetime prevalence of 0.4–0.9% [1–3] and 16.2–19.5% [4–6], respectively. Both contribute substantially to the global burden of disease [7]. The symptomatology of SCZ includes negative symptoms (e.g., flat affect), positive symptoms (e.g., hallucinations and delusions), as well as cognitive impairment (e.g., disorganised thinking) [8]. Common MDD symptoms range from sadness and irritability [9] to suicidal ideation [10].

According to twin and family studies, SCZ and MDD are influenced by both environmental and heritable factors [11, 12]. Most likely, the development of these disorders involves an intricate interplay between genetic and environmental risk factors rather than independent effects. In this study, we investigate one pattern of such interplay, gene-environment correlation (rGE), using polygenic scores for SCZ and MDD (based on existing genome-wide association study results) in two British community samples of adults. In addition to testing whether established environmental risk factors in adulthood are correlated with the genetic liability for SCZ/MDD, we also investigate whether associations differ between the two psychopathologies and the two featured generations.

### Genetic risk for SCZ and MDD

In light of the substantial heritability of both disorders which ranges from 64 to 81% for SCZ [12–14] and 34 to 39% for MDD [15–17] based on twin, family and adoption studies, several molecular genetic studies set out to identify genetic variants underlying these disorders. However, despite several genome-wide association studies (GWAS) in very large samples, variants that survived correction for multiple testing currently only explain a small proportion of the heritability [18–20]. For instance, an early analysis of the SCZ Working Group from the Psychiatric Genomics Consortium (PGC) identified five associated variants in 9,394 cases, whereas the first MDD PGC mega-analysis in 9,240 cases was underpowered to detect any significant variants [21, 22] with the current SNP heritability estimated to be ~33% for SCZ and 8.7% for MDD [23, 24]. These findings suggest that the genetic architecture of these disorders is likely highly polygenic with many variants of small effect [18, 25]. Nevertheless, by summing the individual weights of thousands of variants into polygenic risk scores (PRS), it has become possible to quantify the genetic risk for these psychopathologies [26–29]. That said, although PRSs have shown promise [30, 31], a substantial proportion of the heritable variance of these disorders remains unexplained.

A further important point to consider is that whilst SCZ and MDD are regarded clinically distinct psychopathologies, depressive symptoms are common in individuals who experience psychotic episodes [32] with GWAS studies of five psychiatric disorders, including SCZ and MDD, suggesting some genetic overlap between these psychopathologies [33, 34]. Additionally, findings from the SCZ Working Group of the PGC [35] and the MDD Working Group of the PGC [23], confirm a partial genetic overlap between both disorders but also identified disorder-specific variants.

### Environmental risk for SCZ and MDD

Many studies point to the important role of environmental and psychosocial risk factors in the development of SCZ and MDD [12, 15], with substance abuse, including alcohol consumption [36, 37] and smoking [38, 39], perhaps being the risk factor that research primarily focused on. Other psychosocial risk factors such as unemployment [40–42], low socio-economic status (SES) [43, 44] and lower educational attainment [45, 46] also appear to be implicated in the aetiology of SCZ and MDD. Moreover, separation/divorce emerged as a relevant psychosocial risk factor with a bi-directional relationship in individuals with MDD. In other words, people who are separated or divorced are more likely to develop MDD, but individuals with MDD have also an increased risk of experiencing marital disruption [47]. Similarly, individuals suffering from SCZ are less likely to be married [48], with early onset SCZ associated with worse marital outcome [49].

### Gene-environment interplay

Whilst classic twin and family studies imply that genetic and environmental influences contribute independently to the aetiology of psychopathology, an increasing number of studies suggest that these factors are likely intertwined through a complex interplay [50]. One form of such gene-environment interplay suggests that the genetic susceptibility to mental health disorders may be associated with the exposure to environmental risk factors, resulting in rGE [51, 52]. For instance, Maxwell et al. (2019) tested whether 420 behavioural traits relating to lifestyle, nutrition, psychology and personality, were associated with the PRS for SCZ in a sub-cohort of 307,823 psychiatrically healthy participants from the UK Biobank [53]. The study identified that the genetic liability for SCZ was correlated with 101 out of the 420 traits, including higher odds for self-reported risk taking and smoking [53]. Moreover, another study investigated whether the PRS for MDD was moderated by urbanicity by utilising five indicators of poor mental health as outcomes in 41,198 individuals over the age of 19 years from the Norwegian HUNT study [54]. Results suggested that the PRS for MDD was higher for individuals in urban areas compared to individuals in rural areas which suggests possible rGE [54].

Such findings emphasise the importance of understanding whether and how the genetic risk for psychiatric disorders is associated with exposure to established environmental and psychosocial risk factors in order to prevent and treat these disorders. Where there is evidence for rGE (i.e., the effects of environmental risk appears to be confounded by genetic risk), interventions aimed at reducing genetically confounded environmental risk factors may have little effect on the disease [55]. On the other hand, the absence of rGE may suggest that the investigated environmental risk factors are not confounded by genetic risk and therefore reflect more promising prevention or treatment targets.

### The current study

Given that the specific environmental and psychosocial risk factors for psychopathology may change over time due to cultural factors, it is important to also consider differences between cohorts [56]. The first aim of this study is to explore the presence of rGE in relation to PRSs for SCZ and MDD and a number of established environmental and psychosocial risk factors in individuals older than 16 years in two British cohort studies, the 1958 National Child Development Study (NCDS) and Understanding Society (USoc), which started in 2009. This will help to better understand whether and how genetic and environmental risk factors are associated with each other in the general population. Secondly, we will investigate whether associations between genetic and environmental risk differ between SCZ and MDD given the incomplete genetic overlap between these disorders. Finally, the third aim is to consider cohort effects by comparing rGE results across two different adult samples.

Based on theory and existing empirical research, we expect to (1) detect significant correlations between the PRSs for SCZ/MDD and established environmental and psychosocial risk factors, (2) that rGE findings would differ between the two psychopathologies given the differences in heritability and limited genetic overlap, and (3) that detected rGEs would differ across the two cohorts with different ages due to cultural shifts in environmental and psychosocial risks.

## Methods

### Participants

Existing data were obtained from two British community cohorts with longitudinal data, the 1958 National Child Development Study (NCDS) and the Understanding Society Study (USoc). The NCDS study includes 17,415 unrelated individuals who were born in a specific week in March 1958 in England, Scotland, and Wales [57, 58]. The study was augmented with cohort members who had been born overseas in the relevant week in March 1958 and who had moved to Great Britain at age 7, 11 or 16 years [57, 59]. Surviving cohort members continued to be surveyed throughout their lives. Data collection included repeated surveys of the NCDS cohort as well as a bio-medical survey between the age of 44–45 and DNA collection on 9,340 individuals which was carried out by qualified nurses during 2002–2004 [60, 61] (Table 1).

**Table 1:** Cohort demographics.

Cohort characteristics	NCDS	USoc
Participants	17,415 unrelated individuals	~40,000 households with over 100,000 individuals
Cohort start	1958	2009
Geographic area	England, Scotland and Wales	UK residences
Ethnicity	98% of white background with dataset having been later augmented with immigrants born within the same reference week	~75% (35,920 individuals over 16 years, excluding proxy respondents) of white British background (English, Wales, Scottish or Northern Irish) at wave 1 with dataset having been augmented with Ethnic Minority Boost Sample and a further Ethnic Minority and Immigrant Boost samples
Genetic data	~7,000 individuals	~10,000 individuals
Final cleaned genotype study sample	5,288 individuals	7,384 individuals
Sex	2,668 (50.45%) females 2,620 (49.55%) males	4,281 (57.98%) females 3,103 (42.02%) males
Age	All born in a single week in March 1958	Mixed ages Only individuals over 16 years of age were used for this study (mean age = 52 at wave 1)
Data waves used	6 data points at: age 23 (1981) age 33 (1991) age 42 (2000) age 46 (2004) age 50 (2008) age 55 (2013)	9 data points at: approximately yearly from 2009/2010 (wave 1) until 2017/2018 (wave 9)

*Source:* [57–66, 91].

The USoc study includes approximately 40,000 households from the United Kingdom (UK) of mixed ages, including 6,000 households from the British Household Study, which was supplemented with an Ethnic Minority Boost Sample, and the Immigrant and Ethnic Minority Boost Sample [62–65]. Households are assessed each year through face-to-face interviews or online questionnaires, whereby individuals over the age of 16 complete the adult survey [63]. Adults from the USoc study received a health assessment from a registered nurse in waves 2 and 3 (2010–2012), and DNA samples were collected from approximately 10,000 participants [66] (Table 1).

The datasets for both cohorts include a wide range of phenotypic data as well as genome-wide genetic data [57, 63].

### Measures

#### Environmental risk factors

We selected the following environmental risk factors across three different categories which are implicated in the aetiology of SCZ or MDD: (1) *Economic situation*: unemployment [40–42], financial difficulties [40] and socio-economic status (SES) [43]. We also included additional indicators of SES, such as income, number of bedrooms in the house, and tenure (whether home was rented or owned); (2) *Substance abuse*: alcohol consumption [36, 37], and smoking [38, 39], as well as, (3) *Psychosocial factors*: educational attainment [45] and marital status [47–49] (Supplementary document 1).

#### Genetic data

For NCDS, we analysed genetic data collected as part of three previous genetic studies with NCDS participants: The Wellcome Trust Case Control Consortium (WTCCC1), the Wellcome Trust Case Control Consortium 2 (WTCCC2), and Type 1 Diabetes Genetics Consortium (T1DGC). Data from the WTCCC1 comprised of 1502 individuals (common controls used for case-control comparisons) genotyped using the Affymetrix 500k 1.2 M [67]. The WTCCC2 sample was made up of 2922 individuals (common controls) genotyped using the Illumina 1.2 M array [68] and data from T1DGC comprised of 2592 individuals (controls used for case-control comparisons), genotyped on Infinium Humanhap 550k v3 chips [69]. For USoc, we used genome-wide DNA which was collected as part of the wave 2 and 3 biomedical assessments [70]. The data comprised of 9,961 individuals genotyped by the Wellcome Trust Sanger Institute on the Illumina Infinium HumanCoreExome BeadChip array with more than 250,000 genome-wide tagging SNPs in genome build 37 [71].

#### Quality control, imputation and post-imputation quality control

In both cohorts, quality control (QC) was performed separately for each genetic dataset following Coleman et al. [72] using PLINK 1.9 [73], including the exclusion of duplicated individuals, minor allele frequencies (MAF < 1%), SNPs or individuals with missing data (<99%), SNPs deviating from Hardy-Weinberg-Equilibrium (*p*-value<1 × 10–5) and any related individuals (pi-hat > 0.1875). Additional checks were performed for inconsistencies between genetic and phenotype sex before pruning for linkage disequilibrium (LD) where *r*^2^ < 0.2, and excluding non-autosomal and high-LD regions. We then assessed the genetic files for population stratification (>6 SD from mean) and removed any ancestry outliers before testing for unusual genome-wide heterogeneity (> or <3SD from mean). Forward and reverse coded SNPs were identified with SNPFLIP v0.0.6 [74] and any SNPs with allele frequency mismatches between the European subsample of the 1000 Genomes Project [75] and our data were excluded. The NCDS genetic data was lifted from genome build B36 to B37 for WTCCC2 and T1DGC and from B35 to B37 for WTCCC1 using liftOverPlink [76]. The USoc genetic data did not require a lift-over. Using the Michigan Imputation Server [77], the genetic data was imputed against the 1000 Genomes Project Phase 3 v5 reference panel [75]. Post-imputation QC was performed for each individual genotype file separately. Firstly, using bcftools [78], we filtered on posterior genotype probability imputation confidence (GP threshold of >0.8) as well as imputation quality (R2 > 0.8) before removing duplicate, failed, or missing genetic variants, MAF of <5% and individuals with incomplete genotypes (<99%). In NCDS, WTCCC1, WTCCC2, and T1DGC were combined, and any tri-allelic sites and duplicate individuals excluded. For NCDS only, we repeated the Principal Component Analysis on the combined LD-pruned dataset. The top 5 and 4 principal components which explained the greatest variance were selected as covariates for NCDS and USoc, respectively. The final combined NCDS dataset included 5,288 individuals and 6,398,736 genetic variants. In USoc, 9,039 individuals and 5,218,682 genetic variants passed post-imputation QC (supplementary document 3).

#### Polygenic risk scores for SCZ and MDD

We used existing GWAS results from the MDD Working Group of the PGC [23] and the SCZ Working Group of the PGC [35] to compute polygenic risk scores (PRS) for MDD and SCZ, respectively. However, given that NCDS was used as a control sample in both consortia, the SCZ and MDD GWAS findings were recomputed without the NCDS to avoid sample overlap (for SCZ all UK cohorts except Ireland where excluded; for MDD the NCDS, 23andMe and GenPod samples were excluded). PRSs were computed for each individual using the LD pruned data (*r*^2^ < 0.1) at seven *P*-value thresholds (0.01, 0.1, 0.2, 0.3, 0.4, 0.5, and 1) using PRSice [79].

#### Procedure

Data from NCDS was drawn from six different time points, ranging from 1981 (participants aged 23) until 2013 (participants aged 55). For USoc, we used data from nine different waves, from 2009 to 2010 (including only participants who completed the adult questionnaire aged 16 to 97 years of age, mean age = 52) to 2017–2018 (participants aged 22 to 104). In order to avoid bias by including genetically unrelated individuals from the same household we randomly selected one individual from each family, resulting in a total of 7,384 individuals using STATA v12.1. As this study is focusing on adults, responses from USoc participants who were less than 16 years of age were excluded at each data wave (61 individuals were removed from wave 1 and 16 individuals from wave 2) (supplementary document 3).

According to power analyses, the samples are sufficiently powered to explain 0.5% of the variance using an alpha of .05, except for the variables *tenure* at ages 23 and 55, *employment* at age 23 in NCDS, as well as for *SES* at wave 8–9 in USoc (see supplementary document 2).

### Data analysis

We calculated descriptive statistics, including mean and standard deviation, for NCDS and USoc as well as each cohort’s genotyped sample for each variable at each wave. Differences between cohort samples and the genotyped sub-samples were identified using chi-square tests for binary variables and independent t-tests for continuous/polytomous variables (Supplementary document 2).

For both cohorts, we also calculated correlation matrices using pairwise correlation coefficients to check for multicollinearity across all markers of socio-economic status, including SES, number of bedrooms, tenure, income, financial issues, and employment (Supplementary document 4).

We then ran linear or logistic regressions for environmental variables at a single data wave, whereas linear and logistic mixed-effects regressions or random effects longitudinal models were used to assess any environmental measures with repeated measurements. In both cohorts, all regression models included the principal components, age/year of data collection and sex as covariates. To correct for multiple testing, we applied Bonferroni correction across all variables for both disorders and both cohorts (*p*-value of 0.05 divided by the number of dependent variables [*n* = 30], indicating that a *p* ≤ 1.67 × 10^–3^ was required to reject the null hypothesis). At least one p-value threshold had to meet the Bonferroni correction for the correlation between the environmental risk factor and the genetic susceptibility for SCZ/MDD to be significant.

To assess if any significant findings could have been caused by clinical cases, we conducted sensitivity analyses: (1) In NCDS, significant MDD findings were re-run after removing individuals with depression (*n* = 1397). These individuals answered ‘yes’ to the question from the wave 9 questionnaire asking whether they had suffered from depression since the last interview [80]; (2) In USoc, any statistically significant finding for MDD were re-calculated after removing individuals who reported that they received treatment for psychiatric problems (*n* = 111). In addition, we also removed individuals with a clinical diagnosis of depression (*n* = 448). As no clinical diagnosis or information on symptoms for SCZ were available in USoc, we were not able to conduct the sensitivity analysis in that sample.

Moreover, we performed interaction analyses between independent variables and MDD symptoms for each cohort by using the resulting Wald Chi-squared test statistics to understand if the resulting beta coefficients from the regressions and the sensitivity analyses are statistically different from each other.

All analyses were run in STATA v12.1 [81].

## Results

### Descriptive statistics

We did not detect any statistically significant differences between the whole NCDS cohort and our genotyped target sample for all environmental measures. In USoc, results indicate that there were some statistically significant differences between the complete USoc sample and the genotype sample, whereby individuals in the genotyped sub-sample were less likely to be married at wave 1 and had a lower number of bedrooms at all waves. Our community cohorts were under-powered for tenure at age 23 and 55 and employment at age 23 in NCDS, as well as SES at wave 8 and 9 in USoc (see supplementary document 2).

### rGE findings in NCDS

None of the selected environmental risk factors hypothesised to be associated with the genetic liability to SCZ reached statistical significance after correction for multiple testing in the NCDS cohort. However, the genetic liability for MDD was significant for low SES, low number of bedrooms, rented accommodation, and smoking (but only low number of bedrooms and tenure survived correction for multiple testing). Sensitivity analyses indicated that associations were not confounded by clinical cases after excluding participants with depression (see Table 2 and supplementary document 4).

**Table 2:** NCDS results.

Environment	SCZ				MDD		
	Threshold	Beta	95% CI	*P*-value	Beta	95% CI	*P*-value
SES (5 Professional 4 Managerial 3 Skilled 2 Partly-skilled 1 Unskilled)	0.01	0	−0.03–0.02	9.04E-01	−0.02	−0.04–0.00	6.29E-02
	0.5	0.02	−0.01–0.04	1.89E-01	−0.02	−0.04–0.00	8.95E-02
	1	0.02	−0.01–0.04	1.77E-01	−0.02	−0.04–0.00	1.11E-01
Number of Rooms (continuous)	0.01	0	−0.03–0.02	6.99E-01	−0.03	−0.04-−0.01	8.93E-03*
	0.5	−0.01	−0.03–0.01	3.66E-01	−0.03	−0.05-−0.01	1.51E-03**
	1	−0.01	−0.03–0.01	3.92E-01	−0.03	−0.05-−0.01	1.84E-03*
Marital status (0 = in relationship, 1 = not in relationship)	0.01	0.06	−0.01–0.13	8.36E-02	−0.01	−0.08–0.05	6.89E-01
	0.5	0.02	−0.04–0.09	4.70E-01	−0.02	−0.09–0.04	4.29E-01
	1	0.02	−0.04–0.09	4.91E-01	−0.02	−0.08–0.04	4.77E-01
Smoking (0 = no, 1 = yes)	0.01	0.06	−0.14–0.27	5.52E-01	0.21	0.02–0.40	3.06E-02*
	0.5	0.12	−0.07–0.32	2.11E-01	0.24	0.05–0.42	1.20E-02*
	1	0.12	−0.07–0.32	2.07E-01	0.23	0.05–0.42	1.45E-02*
Employment (0 = Employed, 1 = Unemployed)	0.01	0.08	−0.02–0.18	1.22E-01	0.08	−0.01–0.18	8.92E-02
	0.5	0.08	−0.01–0.18	8.86E-02	0.05	−0.04–0.15	2.78E-01
	1	0.07	−0.02–0.17	1.33E-01	0.04	−0.05–0.14	3.59E-01
Tenure (0 = owns, 1 = rents)	0.01	0.01	−0.11–0.13	8.39E-01	0.1	−0.01–0.20	8.24E-02
	0.5	0	−0.11–0.11	9.62E-01	0.16	0.05–0.27	3.33E-03*
	1	0.01	−0.10–0.12	9.07E-01	0.15	0.04–0.26	6.04E-03*

*Note*: * significant, ** significant after correcting for multiple testing, Not all thresholds have been included (0.1, 0.2, 0.4, 0.4 have been omitted). For MDD tenure, thresholds 0.01 was significant but are not displayed in this table, *NCDS*1958 National Child Developmental Study, *SCZ* Schizophrenia, *MDD* Major Depressive Disorder, *SES* socioeconomic status, *Beta* Beta coefficient, *CI* Confidence Interval.

### rGE findings in USoc

In USoc, the genetic propensity for SCZ was associated with being single or divorced, financial difficulties, as well as unemployment (but only marital status survived correction for multiple testing). The PRS for MDD was associated with low SES, being single or divorced, unemployment, low number of bedrooms, finance issues and low income, of which all but marital status and low SES survived correction for multiple testing. We were unable to run a sensitivity analysis for the SCZ finding due to the lack of information on SCZ cases in USoc. However, according to sensitivity analyses for MDD none of our statistically significant findings were confounded by individuals with depression or those who received psychiatric treatment. (Table 3 and supplementary document 4).

**Table 3:** USoc results.

Environment	SCZ				MDD		
	Threshold	Beta	95%CI	*P*-value	Beta	95%CI	*P*-value
SES (5 Professional 4 Managerial 3 Skilled 2 Partly-skilled 1 Unskilled)	0.01	−0.02	−0.05–0.01	1.45E-01	−0.03	−0.06 to −0.00	2.62E-02*
	0.5	−0.02	−0.05–0.01	2.25E-01	−0.01	−0.04–0.02	4.94E-01
	1	−0.02	−0.05–0.01	2.70E-01	−0.01	−0.04–0.02	4.17E-01
Number of Rooms (continuous)	0.01	−0.02	−0.04–0.01	1.72E-01	−0.04	−0.07-−0.02	4.36E-04**
	0.5	−0.02	−0.04–0.00	8.94E-02	−0.03	−0.05-−0.00	3.46E-02*
	1	−0.02	−0.04–0.00	9.69E-02	−0.03	−0.05-−0.00	2.74E-02*
Marital status (0 = married, 1 = single/divorced)	0.01	0.08	0.02–0.13	5.42E-03*	0.08	0.03–0.14	2.38E-03*
	0.5	0.09	0.04–0.15	1.13E-03**	0.01	−0.04–0.07	6.00E-01
	1	0.09	0.03–0.15	1.63E-03**	0.02	−0.04–0.07	5.18E-01
Income (Grouped into 50 sub-groups)	0.01	−0.02	−0.04–0.00	1.08E-01	−0.03	−0.05-−0.01	6.72E-04**
	0.5	−0.02	−0.04–0.00	7.76E-02	−0.02	−0.04-−0.00	4.75E-02*
	1	−0.02	−0.04–0.00	6.57E-02	−0.02	−0.04-−0.00	4.31E-02*
Alcohol consumption (0 = not drinking, 1–7 = drinking days per week)	0.01	0	−0.02–0.03	9.56E-01	0.01	−0.01–0.04	3.29E-01
	0.5	−0.01	−0.03–0.02	5.80E-01	0	−0.03–0.02	7.07E-01
	1	−0.01	−0.03–0.02	6.42E-01	−0.01	−0.03–0.02	6.25E-01
Employment (0 = employed, 1 = unemployed)	0.01	0.16	0.04–0.27	7.81E-03*	0.2	0.09–0.32	5.50E-04**
	0.5	0.15	0.03–0.26	1.18E-02*	0.21	0.09–0.32	3.98E-04**
	1	0.15	0.04–0.27	8.50E-03*	0.2	0.09–0.31	5.61E-04**
Tenure (0 = owns, 1 = rents)	0.01	0.05	−0.17–0.28	6.48E-01	0.13	−0.10–0.37	2.51E-01
	0.5	0.02	−0.22–0.25	8.86E-01	0.1	−0.14–0.33	4.15E-01
	1	0.01	−0.22–0.25	9.01E-01	0.09	−0.14–0.32	4.29E-01
Finance Issues (0 = no issues, 1 = issues)	0.01	0.12	0.03–0.20	7.99E-03*	0.24	0.16–0.33	2.91E-08**
	0.5	0.14	0.05–0.22	2.29E-03*	0.23	0.14–0.32	1.52E-07**
	1	0.14	0.05–0.22	1.90E-03*	0.23	0.15–0.32	1.36E-07**
Education (0 = A-level or above, 1 = GCSE or below)	0.01	0	−0.36–0.37	9.94E-01	0.2	−0.17–0.57	2.96E-01
	0.5	−0.05	−0.42–0.32	7.88E-01	0.09	−0.27–0.46	6.18E-01
	1	−0.05	−0.42–0.32	7.90E-01	0.1	−0.27–0.46	6.03E-01

Note: * = significant, ** = significant after correcting for multiple testing, Not all thresholds have been included (0.1, 0.2, 0.4, 0.4 have been omitted), *USoc* Understanding Society, *SCZ* Schizophrenia, *MDD* Major Depressive Disorder, *SES* socioeconomic status, *Beta* Beta coefficient, *CI* Confidence Interval.

## Discussion

The first aim of this study was to investigate whether the genetic vulnerability for SCZ and MDD, measured with PRSs from existing large genome-wide association studies, was associated with established environmental and psychosocial risk factors in individuals over the age of 16 across two British community samples.

### rGE results for SCZ

Our finding shows that the genetic risk for SCZ is associated with a reduced likelihood of being married. This association may reflect evocative rGE [82] whereby the genetic propensity for SCZ evokes negative reactions in others which then leads to relationship problems. However, all significant SCZ findings emerged in USoc only and without information on SCZ cases or symptoms, we were not able to exclude the alternative explanation that the detected associations reflect indirect effects of SCZ symptoms, including psychotic episodes, hallucinations and cognitive disabilities with detrimental consequences in work and family.

### rGE results for MDD

We identified significant associations between the genetic risk for MDD and several indicators of low SES, including unemployment, low income, financial difficulties as well as rented accommodation and low number of bedrooms. Although it is important to highlight that the result for SES did not survive correction for multiple testing. Further, our environmental correlation matrices suggest that several indicators of low SES are correlated with each other in both cohorts. Previous studies indicate that a lower SES is associated with a range of mental health disorders, including depressive symptoms [83, 84]. Specifically, measures of SES, such as low income and unemployment, were predicting antidepressant treatment responses in more than 2500 patients with MDD who participated in the Sequenced Treatment Alternatives to Relieve Depression (STAR*D) clinical trial [85]. Therefore, it is possible that individuals with the genetic susceptibility for MDD are more likely to experience subclinical symptoms of psychopathology which may prevent them from securing work or advancing in their careers. This in turn could contribute to difficulties purchasing a home as observed in NCDS in the current study. Consequently, some of these environmental associations may reflect intermediate phenotypes of subclinical disease as opposed to a causal pathway to depression itself, or most likely a combination of both. While we cannot test the causality of these findings, our sensitivity analyses showed that these association were not confounded by clinical cases in either cohort. Hence, it is also plausible that the correlated risk factors reflect at least partially genetic confounding through active rGE which occurs when individuals select themselves into environments based on their genetic susceptibility [82]. In other words, the genetic risk for MDD may be mediating through low SES into which individuals selected themselves into.

### Differences in rGE results between SCZ and MDD

The second objective of our study was to investigate whether rGEs differ between SCZ and MDD. Our study confirmed that there was only one significant finding for SCZ and five for MDD, with none of these associations matching between the two psychopathologies. If the detected correlations are indeed due to genetic confounding, then such differences could be explained by the incomplete genetic overlap between the two psychopathologies.

### Differences in rGE results between cohorts

Our third goal was to identify whether associations between the two community samples differ. Interestingly, we found significant differences in rGE results between the two studies. Firstly, for SCZ, we only obtained one significant finding in USoc but not in NCDS. One possible explanation for this outcome is that investigating psychopathologies, such as SCZ, with a low base rate will result in less powerful PRS predications in the general population [86]. Consequently, replicating the same results across different community cohorts of different sizes may be more challenging. Secondly, MDD findings in USoc and NCDS did not match completely for environments which were available in both cohorts. For instance, the correlation between the genetic liability to MDD and unemployment was only found in USoc but not in NCDS. On the other hand, the PRS for MDD was associated with house ownership in NCDS but not in USoc. It is possible that differences within and between the two community cohorts could be attributed to either cohort or age effects, whereby differential exposures to particular environmental risk factors are not just unique to that cohort population but also to the participants who enter the study at different ages [56, 87]. For instance, all individuals in NCDS were born in 1958 versus a mixed age group in USoc. Therefore, future research should explore whether there is a fundamental discrepancy between possible rGE findings at different developmental stages, such as adolescence, early and late adulthood.

### Strengths and limitations

Our study has several strengths, such as inclusion of various environmental risk factors and consideration of two different psychopathologies in two established and well characterised cohort studies. However, our findings should take into account a number of limitations. First, we did not have information on SCZ cases or symptoms in the USoc dataset to assess whether there was confounding by clinical cases. Second, according to our power analysis some of the tested associations were under-powered. Third, the USoc subsamples with genetic data differed from the original cohort samples in respect to marital status at wave 1 and number of bedrooms at all waves. Therefore, the current findings may not generalise to the original cohorts. Fourthly, it is also important to highlight that the applied PRSs have been derived from associations in datasets that did not account for the influence of environmental factors. In other words, it is not clear to what degree the applied PRSs reflect associated environment risk. Hence, the specificity of our rGE results between SCZ and MDD need to be considered with this caveat in mind. Finally, not all selected environmental measures were available across both cohorts. Thus, the comparison between NCDS and USoc should be considered exploratory.

### Implications

Whilst rGE may mirror the pleiotropic effects of genes on the environment and the disease process, which subsequently would render environmental interventions ineffective, it is important to put the findings into context [82]. Although a high PRS indicates an increased risk for SCZ or MDD, they are in no way deterministic and should not be interpreted as such. PRSs explain only a small portion of the phenotypic variance and the field will need to overcome substantial challenges before these can be implemented in a clinical setting [88]. Moreover, our study was conducted in two British community cohorts and utilised GWAS findings from participants of mostly European ancestry from high income countries [23, 35]. Therefore, the predictive power of the PRS will be higher in individuals from these populations and cannot be generalised to other populations [89]. Furthermore, although our study suggests that the correlations between known environmental risk factors in adults and the genetic liability for SCZ and MDD could be at least partially genetically confounded, it does not preclude the possibility that these adverse environmental exposures can also have causal effects on the progression of the two psychopathologies [90]. Our study does not aim to fully explain the complex interplay between genes and environments and our findings do not suggest that subsequent targets or interventions for SCZ or MDD will be completely unsuccessful. Environmental risk factors, such as financial problems, are incredibly complex and the result of a myriad of factors, including genetic and non-genetic influences. Thus, more research is needed to disentangle this complex interplay in adults, such as using cohorts with intergenerational data to help assess the type of rGE present between environmental risk factors and the genetic risk to SCZ and MDD.

## Conclusion

According to analyses of two British cohorts, the genetic propensities for MDD in individuals from the general population was associated with various markers of socio-economic as well as social adversity. This suggests that rGE may contribute to the aetiology of MDD whereby individuals may select themselves into adverse environments which are correlated with their genetic predisposition. Furthermore, findings suggest that environmental risk factors that are associated with a genetic risk for psychopathology are largely disorder specific, in that SCZ was more strongly associated with psychosocial risk including being divorced/separated, whereas MDD was more correlated with indicators of low SES. Finally, detected rGEs differed between cohorts suggesting that the influence of genetic risk on environmental risk factors may change over time due to societal changes. In sum, our study provides further evidence that environmental and psychosocial risk factors for psychiatric disorders are influenced by the genetic risk for these disorders. Previously identified risk factors for SCZ and MDD may not necessarily have a causal function but mediate a genetic susceptibility to these disorders. More research is needed to disentangle the true causality between environmental risk factors and the genetic susceptibility to SCZ and MDD. For instance, using cohorts with genotypes from multiple generations, such as parental genotypes in addition to the individual’s genotypes, would allow for the identification of the specific type of rGE for these psychopathologies.

## Supplementary information

Supplementary_1_Coded_variables

Supplementary_2_Descriptive_statistics

Supplementary_3_Genetic_QC

Supplementary_4_SCZ_and_MDD_results

## References

[1] McGrath J, Saha S, Chant D, Welham J (2008). Schizophrenia: a concise overview of incidence, prevalence, and mortality. Epidemiol Rev.

[2] Perälä J, Suvisaari J, Saarni SI, Kuoppasalmi K, Isometsä E, Pirkola S (2007). Lifetime prevalence of psychotic and bipolar I disorders in a general population. Arch Gen Psychiatry.

[3] Saha S, Chant D, Welham J, McGrath J (2005). A systematic review of the prevalence of schizophrenia. PLoS Med.

[4] Kendler KS, Gatz M, Gardner CO, Pedersen NL (2006). A Swedish national twin study of lifetime major depression. Am J Psychiatry.

[5] Kessler RC, Berglund P, Demler O, Jin R, Koretz D, Merikangas KR (2003). The epidemiology of major depressive disorder: results from the National Comorbidity Survey Replication (NCS-R). JAMA.

[6] Kruijshaar ME, Barendregt J, Vos T, de Graaf R, Spijker J, Andrews G (2005). Lifetime prevalence estimates of major depression: an indirect estimation method and a quantification of recall bias. Eur J Epidemiol.

[7] Whiteford HA, Degenhardt L, Rehm J, Baxter AJ, Ferrari AJ, Erskine HE (2013). Global burden of disease attributable to mental and substance use disorders: findings from the Global Burden of Disease Study 2010. Lancet.

[8] Gogtay N, Vyas NS, Testa R, Wood SJ, Pantelis C (2011). Age of onset of schizophrenia: perspectives from structural neuroimaging studies. Schizophr Bull.

[9] Lopez Molina MA, Jansen K, Drews C, Pinheiro R, Silva R, Souza L (2014). Major depressive disorder symptoms in male and female young adults. Psychol Health Med.

[10] Du L, Bakish D, Lapierre YD, Ravindran AV, Hrdina PD (2000). Association of polymorphism of serotonin 2A receptor gene with suicidal ideation in major depressive disorder. Am J Med Genet.

[11] Kendler KS, Karkowski-Shuman L (1997). Stressful life events and genetic liability to major depression: genetic control of exposure to the environment?. Psychol Med.

[12] Sullivan PF, Kendler KS, Neale MC (2003). Schizophrenia as a complex trait: evidence from a meta-analysis of twin studies. Arch Gen Psychiatry.

[13] Hilker R, Helenius D, Fagerlund B, Skytthe A, Christensen K, Werge TM (2018). Heritability of schizophrenia and schizophrenia spectrum based on the Nationwide Danish Twin Register. Biol Psychiatry.

[14] Lichtenstein P, Yip BH, Björk C, Pawitan Y, Cannon TD, Sullivan PF (2009). Common genetic determinants of schizophrenia and bipolar disorder in Swedish families: a population-based study. Lancet.

[15] Sullivan PF, Neale MC, Kendler KS (2000). Genetic epidemiology of major depression: review and meta-analysis. Am J Psychiatry.

[16] Kendler KS, Prescott CA (1999). A population-based twin study of lifetime major depression in men and women. Arch Gen Psychiatry.

[17] Nes RB, Czajkowski NO, Røysamb E, Orstavik RE, Tambs K, Reichborn-Kjennerud T (2013). Major depression and life satisfaction: a population-based twin study. J Affect Disord.

[18] Gratten J, Wray NR, Keller MC, Visscher PM (2014). Large-scale genomics unveils the genetic architecture of psychiatric disorders. Nat Neurosci.

[19] Plomin R (2013). Commentary: missing heritability, polygenic scores, and gene-environment correlation. J Child Psychol Psychiatry.

[20] Levinson DF, Mostafavi S, Milaneschi Y, Rivera M, Ripke S, Wray NR (2014). Genetic studies of major depressive disorder: why are there no genome-wide association study findings and what can we do about it?. Biol Psychiatry.

[21] Ripke S, Wray NR, Lewis CM, Hamilton SP, Weissman MM, Breen G (2013). A mega-analysis of genome-wide association studies for major depressive disorder. Mol Psychiatry.

[22] Schizophrenia Psychiatric Genome-Wide Association Study (GWAS) Consortium. (2011). Genome-wide association study identifies five new schizophrenia loci. Nat Genet.

[23] Wray NR, Ripke S, Mattheisen M, Trzaskowski M, Byrne EM, Abdellaoui A (2018). Genome-wide association analyses identify 44 risk variants and refine the genetic architecture of major depression. Nat Genet.

[24] Ripke S, O’Dushlaine C, Chambert K, Moran JL, Kähler AK, Akterin S (2013). Genome-wide association analysis identifies 13 new risk loci for schizophrenia. Nat Genet.

[25] Culverhouse RC, Saccone NL, Horton AC, Ma Y, Anstey KJ, Banaschewski T (2018). Collaborative meta-analysis finds no evidence of a strong interaction between stress and 5-HTTLPR genotype contributing to the development of depression. Mol Psychiatry.

[26] Dudbridge F, Newcombe PJ (2015). Accuracy of Gene Scores when Pruning Markers by Linkage Disequilibrium. Hum Hered.

[27] Dudbridge F (2013). Power and predictive accuracy of polygenic risk scores. PLoS Genet.

[28] Wray NR, Goddard ME, Visscher PM (2007). Prediction of individual genetic risk to disease from genome-wide association studies. Genome Res.

[29] Holland D, Wang Y, Thompson WK, Schork A, Chen CH, Lo MT (2016). Estimating effect sizes and expected replication probabilities from GWAS summary statistics. Front Genet.

[30] Lee JJ, McGue M, Iacono WG, Chow CC (2018). The accuracy of LD Score regression as an estimator of confounding and genetic correlations in genome-wide association studies. Genet Epidemiol.

[31] Bulik-Sullivan B, Finucane HK, Anttila V, Gusev A, Day FR, Loh PR (2015). An atlas of genetic correlations across human diseases and traits. Nat Genet.

[32] Sönmez, N, Romm, KL, Andreasssen, OA, Melle, I, & Røssberg, JI. Depressive symptoms in first episode psychosis: a one-year follow-up study. BMC Psychiatry 2013; 13. 10.1186/1471-244X-13-106 PMC3635985 23560591

[33] Cross-Disorder Group of the Psychiatric Genomics Consortium. (2013). Identification of risk loci with shared effects on five major psychiatric disorders: a genome-wide analysis. Lancet.

[34] Schulze TG, Akula N, Breuer R, Steele J, Nalls MA, Singleton AB (2014). Molecular genetic overlap in bipolar disorder, schizophrenia, and major depressive disorder. World J Biol Psychiatry.

[35] Schizophrenia Working Group of the Psychiatric Genomics Consortium. (2014). Biological insights from 108 schizophrenia-associated genetic loci. Nature.

[36] Grant BF, Stinson FS, Dawson DA, Chou SP, Dufour MC, Compton W (2004). Prevalence and co-occurrence of substance use disorders and independent mood and anxiety disorders: results from the National Epidemiologic Survey on Alcohol and Related Conditions. Arch Gen Psychiatry.

[37] Nielsen SM, Toftdahl NG, Nordentoft M, Hjorthøj C (2017). Association between alcohol, cannabis, and other illicit substance abuse and risk of developing schizophrenia: a nationwide population based register study. Psychol Med.

[38] de Leon J, Diaz FJ (2005). A meta-analysis of worldwide studies demonstrates an association between schizophrenia and tobacco smoking behaviors. Schizophr Res.

[39] Pasco JA, Williams LJ, Jacka FN, Ng F, Henry MJ, Nicholson GC (2008). Tobacco smoking as a risk factor for major depressive disorder: population-based study. Br J Psychiatry.

[40] Kendler KS, Karkowski LM, Prescott CA (1999). Causal relationship between stressful life events and the onset of major depression. Am J Psychiatry.

[41] Evensen S, Wisløff T, Lystad JU, Bull H, Ueland T, Falkum E (2016). Prevalence, employment rate, and cost of schizophrenia in a high-income welfare society: a population-based study using comprehensive health and welfare registers. Schizophr Bull.

[42] Marwaha S, Johnson S, Bebbington P, Stafford M, Angermeyer MC, Brugha T (2007). Rates and correlates of employment in people with schizophrenia in the UK, France and Germany. Br J Psychiatry.

[43] Harrison G, Gunnell D, Glazebrook C, Page K, Kwiecinski R (2001). Association between schizophrenia and social inequality at birth: case-control study. Br J Psychiatry.

[44] Freeman A, Tyrovolas S, Koyanagi A, Chatterji S, Leonardi M, Ayuso-Mateos JL (2016). The role of socio-economic status in depression: results from the COURAGE (aging survey in Europe). BMC Public Health.

[45] Cohen AK, Nussbaum J, Weintraub MLR, Nichols CR, Yen IH (2020). Association of adult depression with educational attainment, aspirations, and expectations. Prev Chronic Dis.

[46] Hakulinen C, McGrath JJ, Timmerman A, Skipper N, Mortensen PB, Pedersen CB (2019). The association between early-onset schizophrenia with employment, income, education, and cohabitation status: nationwide study with 35 years of follow-up. Soc Psychiatry Psychiatr Epidemiol.

[47] Bulloch AG, Williams JV, Lavorato DH, Patten SB (2009). The relationship between major depression and marital disruption is bidirectional. Depress Anxiety.

[48] Walid, MS & Zaytseva, NV. Which neuropsychiatric disorder is more associated with divorce? Journal of Divorce & Remarriage. 2011: 220–4.

[49] Deshmukh V, Bhagat A, Shah N, Sonavane S, Desousa A (2016). Factors affecting marriage in schizophrenia: a cross‐sectional study. J Ment Health Hum Behav.

[50] Kwong ASF, López-López JA, Hammerton G, Manley D, Timpson NJ, Leckie G (2019). Genetic and environmental risk factors associated with trajectories of depression symptoms from adolescence to young adulthood. JAMA Netw Open.

[51] Plomin R, DeFries JC, Loehlin JC (1977). Genotype-environment interaction and correlation in the analysis of human behavior. Psychol Bull.

[52] Jaffee SR, Price TS (2008). Genotype-environment correlations: implications for determining the relationship between environmental exposures and psychiatric illness. Psychiatry.

[53] Maxwell, J, Socrates, A, Glanville, KP, Forti, MD, Murray, RM, Vassos, E, et al., Investigating the role of behaviour in the genetic risk for schizophrenia. bioRxiv, 2019: 611079.

[54] Sund ER, van Lenthe FJ, Avendano M, Raina P, Krokstad S (2021). Does urbanicity modify the relationship between a polygenic risk score for depression and mental health symptoms? Cross-sectional evidence from the observational HUNT Study in Norway. J Epidemiol Community Health.

[55] Wagner B, Li J, Liu H, Guo G (2013). Gene-environment correlation: difficulties and a natural experiment-based strategy. Am J Public Health.

[56] Keyes KM, Nicholson R, Kinley J, Raposo S, Stein MB, Goldner EM (2014). Age, period, and cohort effects in psychological distress in the United States and Canada. Am J Epidemiol.

[57] Power C, Elliott J (2006). Cohort profile: 1958 British birth cohort (National Child Development Study). Int J Epidemiol.

[58] Centre for Longitudinal Studies, National Child Development Study: Childhood Data, Sweeps 0–3, 1958–1974 [data collection]. 2014, National Birthday Trust Fund, National Children’s Bureau: London.

[59] Bann D, Johnson W, Li L, Kuh D, Hardy R (2018). Socioeconomic inequalities in childhood and adolescent body-mass index, weight, and height from 1953 to 2015: an analysis of four longitudinal, observational, British birth cohort studies. Lancet Public Health.

[60] Centre for Longitudinal Studies, National Child Development Study: Biomedical Survey, 2002–2004. 2020, UCL Centre for Longitudinal Studies: London.

[61] Brown M, Goodman A (2014). National Child Development Study (or 1958 Birth Cohort). J Open Health Data.

[62] University of Essex, Institute for Social and Economic Research, NatCen Social Research, & Kantar Public, Understanding Society: Waves 1–9, 2009–2018 and Harmonised BHPS: Waves 1–18, 1991–2009. [data collection]. 2019, UK Data Service.

[63] Buck, N & McFall, S. Understanding Society: design overview. 2011, 2011. 3: 13.

[64] University of Essex and Institute for Social and Economic Research. https://www.understandingsociety.ac.uk/about/about-the-study. [accessed 04 Nov 2021].

[65] Lynn, P. Sample design for understanding society. Underst. Soc. Work. Pap. Ser, 2009. 2009.

[66] University of Essex, Institute for Social and Economic Research, and National Centre for Social Research, Understanding Society: Waves 2 and 3 Nurse Health Assessment, 2010- 2012 [data collection]. 2014, UK Data Service.

[67] Wellcome Trust Case Control Consortium. (2007). Genome-wide association study of 14,000 cases of seven common diseases and 3,000 shared controls. Nature.

[68] WTCCC2. www.wtccc.org.uk/ccc2/. [accessed 23 Sep 2021].

[69] Barrett JC, Clayton DG, Concannon P, Akolkar B, Cooper JD, Erlich HA (2009). Genome-wide association study and meta-analysis find that over 40 loci affect risk of type 1 diabetes. Nat Genet.

[70] Benzeval, M, Davillas, A, Kumari, M, & Lynn, P. Understanding Society: The UK Household Longitudinal Study Biomarker User Guide and Glossary 2014, Institute for Social and Economic Research, University of Essex: Colchester.

[71] Prins BP, Kuchenbaecker KB, Bao Y, Smart M, Zabaneh D, Fatemifar G (2017). Genome-wide analysis of health-related biomarkers in the UK Household Longitudinal Study reveals novel associations. Sci Rep..

[72] Coleman JR, Euesden J, Patel H, Folarin AA, Newhouse S, Breen G (2016). Quality control, imputation and analysis of genome-wide genotyping data from the Illumina HumanCoreExome microarray. Brief Funct Genomics.

[73] Chang CC, Chow CC, Tellier LC, Vattikuti S, Purcell SM, Lee JJ (2015). Second-generation PLINK: rising to the challenge of larger and richer datasets. Gigascience.

[74] Bakken Stovner, E., snpflip. 2017: GitHub.

[75] Auton A, Brooks LD, Durbin RM, Garrison EP, Kang HM, Korbel JO (2015). A global reference for human genetic variation. Nature.

[76] Ritchie, S, liftOverPlink. 2014, GitHub.

[77] Das S, Forer L, Schönherr S, Sidore C, Locke AE, Kwong A (2016). Next-generation genotype imputation service and methods. Nat Genet.

[78] Danecek, P, Bonfield, JK, Liddle, J, Marshall, J, Ohan, V, Pollard, MO, et al. Twelve years of SAMtools and BCFtools. Gigascience, 2021.10. 10.1093/gigascience/giab008 PMC7931819 33590861

[79] Euesden J, Lewis CM, O’Reilly PF (2015). PRSice: Polygenic Risk Score software. Bioinformatics.

[80] University of London, Institute of Education, and Centre for Longitudinal Studies, National Child Development Study: Age 55, Sweep 9, 2013. [data collection]. 2020, UK Data Service: London.

[81] StataCorp, Stata Statistical Software: Release 12. 2011, College Station, TX: StataCorp LP.

[82] Jaffee SR, Price TS (2007). Gene-environment correlations: a review of the evidence and implications for prevention of mental illness. Mol Psychiatry.

[83] Stansfeld SA, Head J, Marmot MG (1998). Explaining social class differences in depression and well-being. Soc Psychiatry Psychiatr Epidemiol.

[84] Muntaner C, Eaton WW, Diala C, Kessler RC, Sorlie PD (1998). Social class, assets, organizational control and the prevalence of common groups of psychiatric disorders. Soc Sci Med.

[85] Wertz J, Belsky J, Moffitt TE, Belsky DW, Harrington H, Avinun R (2019). Genetics of nurture: a test of the hypothesis that parents’ genetics predict their observed caregiving. Dev Psychol.

[86] Anderson JS, Shade J, DiBlasi E, Shabalin AA, Docherty AR (2019). Polygenic risk scoring and prediction of mental health outcomes. Curr Opin Psychol.

[87] Sutin AR, Terracciano A, Milaneschi Y, An Y, Ferrucci L, Zonderman AB (2013). The effect of birth cohort on well-being: the legacy of economic hard times. Psychol Sci.

[88] Lewis CM, Vassos E (2017). Prospects for using risk scores in polygenic medicine. Genome Med.

[89] Palk AC, Dalvie S, de Vries J, Martin AR, Stein DJ (2019). Potential use of clinical polygenic risk scores in psychiatry - ethical implications and communicating high polygenic risk. Philos Ethics Humanit Med.

[90] Knafo A, Jaffee SR (2013). Gene-environment correlation in developmental psychopathology. Dev Psychopathol.

[91] McFall, S, A Nandi, and L Platt, Understanding society: UK household longitudinal study: user guide to ethnicity and immigration research. Institute for Social and Economic Research, 2017.

