# Supplementary material for: Polygenic risk scores for schizophrenia and major depression are associated with socio-economic indicators of adversity in two British community samples
Source: Transl Psychiatry. 2022 Nov 14;12:477. doi: 10.1038/s41398-022-02247-8 (PMC9663827; doi:10.1038/s41398-022-02247-8)
Supplement: Supplementary file 1 — Supplementary_1_Coded_variables [file 41398_2022_2247_MOESM1_ESM.docx]

Supplementary Document 1 – Coded Variables

Table of Contents

[Supplementary Table 1: Breakdown of all coded variables for each wave for NCDS 2](#_Toc99969974)

[Supplementary Table 2: Breakdown of all coded variables for each wave for USoc 4](#_Toc99969975)

[References 8](#_Toc99969976)

# **Supplementary Table 1**: Breakdown of all coded variables for each wave for NCDS

| **Environment** | **Variable** | | **Coded as** | **Variable timeline (Age of participant)** | | | | | | **Mixed-effect regression** | |
| --- | --- | --- | --- | --- | --- | --- | --- | --- | --- | --- | --- |
|  | **Code** | **Name** |  | **23** | **33** | **42** | **46** | **50** | **55** | **Random slope** | **Random Slope + intercept** |
| **SES** | N6149 | 4I Current or last job 1980 social class | 5 Professional  4 Managerial/ occupations 3 Skilled 2 Partly-skilled  1 Unskilled | X |  |  |  |  |  |  | X |
|  | N540056 | CASOC2:2 A4a/b) CURRENT/LAST JOB: RGs Social Class 1981 |  |  | X |  |  |  |  |  |  |
|  | SC | (Current Job) Social Class |  |  |  | X |  |  |  |  |  |
|  | N7SC | (Derived) Social Class (RGSC SC based on Occ 1990) |  |  |  |  | X |  |  |  |  |
|  | N8SC | [SC2] Curr Job: Social Class (RGSC SC based on Occ 1990) |  |  |  |  |  | X |  |  |  |
|  | N9CSC | Social class 1990 based on soc2010 (CM current job) |  |  |  |  |  |  | X |  |  |
| **Employment** | N4755 | 4I Whether currently unemployed | 0 = Employed or Self-employed (full or part-time)  1 = Unemployed, sick, disabled  Removed retired | X |  |  |  |  |  | X |  |
|  | ECONACT | CMs current main activity |  |  |  | X |  |  |  |  |  |
|  | N8ECON02 | [ECONACT2] (Recoded) CM's current economic activity |  |  |  |  |  | X |  |  |  |
|  | ND9ECACT | (Derived) Current economic activity status |  |  |  |  |  |  | X |  |  |
| **Number of Rooms** | N5323 | 4I Number of bedrooms | Continuous variable | X |  |  |  |  |  | X |  |
|  | N502947 | CMI:57 D9 No. rooms  (apart from the bathroom & kitchen) |  |  | X |  |  |  |  |  |  |
|  | BEDROOMS | Number of bedrooms in current acco |  |  |  | X |  |  |  |  |  |
|  | N7NUMRMS | Number of rooms in the house |  |  |  |  | X |  |  |  |  |
|  | ND8NUMRM | (Derived) Number of rooms in the house (n8numrms) |  |  |  |  |  | X |  |  |  |
|  | N9NUMRMS | Number of rooms in home |  |  |  |  |  |  | X |  |  |
| **Tenure** | N5333 | 4I Whether owner or renter | 0 = owns/part-owns  1 = rents (including social housing) | X |  |  |  |  |  | X |  |
|  | TENURE91 | DV:Housing tenure in 1991 |  |  | X |  |  |  |  |  |  |
|  | TENURE2 | Is current accom owned or rented |  |  |  | X |  |  |  |  |  |
|  | N7TEN | Home ownership / tenure status |  |  |  |  | X |  |  |  |  |
|  | N8TEN | [TENURE] Home ownership / tenure status |  |  |  |  |  | X |  |  |  |
|  | N9TEN | Whether CM owns or rents home or some other arrangement |  |  |  |  |  |  | X |  |  |
| **Marital status** | PARTSTAT | 4D Current partnership status | 0 = married/ co-habiting/ in relationship  1 = not in relationship | X |  |  |  |  |  | X |  |
|  | N502549 | CMI:51 C52 CM is cohabiting/married other |  |  | X |  |  |  |  |  |  |
|  | DMSPPART | Whether CM had current  partner in hhld in NCDS V (FF) |  |  |  | X |  |  |  |  |  |
|  | N7MS12 | Person's marital status - 02 |  |  |  |  | X |  |  |  |  |
|  | ND8SPPHH | (Derived) Cohort member lives with a spouse or partner |  |  |  |  |  | X |  |  |  |
|  | ND9COHAB | (Derived) Whether CM cohabiting as a couple |  |  |  |  |  |  | X |  |  |
| **Smoking** | CURRENTN | 4D Smoking patterns | 0 = Never smoked  1 = Smoker/ Ex-smoker | X |  |  |  |  |  | X |  |
|  | SMOKING | CM current smoking status |  |  |  | X |  |  |  |  |  |
|  | N8SMOKIG | [SMOKING] Smoking frequency |  |  |  |  |  | X |  |  |  |
| **Depression** | N9EMOP01 | Type of Emotional Problem - Depression | 0 = No  1 = Yes |  |  |  |  |  | X | Used for sensitivity analysis only | |

Note: Any ‘N/A’, ‘Don’t know’ or ‘other’ answers have been excluded from the analysis. All variables were coded in STATA v12.1 (1)

# **Supplementary Table 2:** Breakdown of all coded variables for each wave for USoc

| **Environment** | **Variable** | | **Coded as** | **Wave** | | | | | | | | | **Mixed-effect regression** | | **Random effects model** |  |
| --- | --- | --- | --- | --- | --- | --- | --- | --- | --- | --- | --- | --- | --- | --- | --- | --- |
|  | **Code** | **Name** |  | **1** | **2** | **3** | **4** | **5** | **6** | **7** | **8** | **9** | **Random slope** | **Random slope + intercept** |  | |
| **Alcohol intake** | b_scnalcl7d | On how many days did you have an alcoholic drink? | Coded as number of days per week on which alcohol is consumed |  | X |  |  |  |  |  |  |  | X |  |  | |
|  | e_scnalcl7d | number of days had alcoholic drink in last 7 |  |  |  |  |  | X |  |  |  |  |  |  |  |  |
| **Education** | a_hiqual_dv | Highest qualification ever reported | 0 = Educated to A-level or above  1 = Educated to GSCE level or below | X |  |  |  |  |  |  |  |  | X |  |  | |
|  | b_hiqual_dv | Highest qualification |  |  | X |  |  |  |  |  |  |  |  |  |  |  |
|  | c_hiqual_dv | Highest qualification |  |  |  | X |  |  |  |  |  |  |  |  |  |  |
|  | d_hiqual_dv | Highest qualification |  |  |  |  | X |  |  |  |  |  |  |  |  |  |
|  | e_hiqual_dv | Highest qualification |  |  |  |  |  | X |  |  |  |  |  |  |  |  |
|  | f_hiqual_dv | Highest qualification |  |  |  |  |  |  | X |  |  |  |  |  |  |  |
|  | g_hiqual_dv | Highest qualification |  |  |  |  |  |  |  | X |  |  |  |  |  |  |
|  | h_hiqual_dv | Highest qualification |  |  |  |  |  |  |  |  | X |  |  |  |  |  |
|  | i_hiqual_dv | Highest qualification |  |  |  |  |  |  |  |  |  | X |  |  |  |  |
| **Number of Rooms** | a_hsbeds | number of bedrooms (top-coded) | Continuous variable | X |  |  |  |  |  |  |  |  |  | X |  | |
|  | b_hsbeds | number of bedrooms (top-coded) |  |  | X |  |  |  |  |  |  |  |  |  |  |  |
|  | c_hsbeds | number of bedrooms (top-coded) |  |  |  | X |  |  |  |  |  |  |  |  |  |  |
|  | d_hsbeds | number of bedrooms (top-coded) |  |  |  |  | X |  |  |  |  |  |  |  |  |  |
|  | e_hsbeds | number of bedrooms (top-coded) |  |  |  |  |  | X |  |  |  |  |  |  |  |  |
|  | f_hsbeds | number of bedrooms (top-coded) |  |  |  |  |  |  | X |  |  |  |  |  |  |  |
|  | g_hsbeds | number of bedrooms (top-coded) |  |  |  |  |  |  |  | X |  |  |  |  |  |  |
|  | h_hsbeds | number of bedrooms (top-coded) |  |  |  |  |  |  |  |  | X |  |  |  |  |  |
|  | i_hsbeds | number of bedrooms (top-coded) |  |  |  |  |  |  |  |  |  | X |  |  |  |  |
| **SES** | a_jbrgsc_dv | Current job: Registrar General's Social Class | 5 = Professional  4 = Managerial-technical  3 = Skilled non-manual and manual  2 = Partly skilled  1 = Unskilled | X |  |  |  |  |  |  |  |  |  | X |  | |
|  | b_jbrgsc_dv | Current job: Registrar General's Social Class |  |  | X |  |  |  |  |  |  |  |  |  |  |  |
|  | c_jbrgsc_dv | Current job: Registrar General's Social Class |  |  |  | X |  |  |  |  |  |  |  |  |  |  |
|  | d_jbrgsc_dv | Current job: Registrar General's Social Class |  |  |  |  | X |  |  |  |  |  |  |  |  |  |
|  | e_jbrgsc_dv | Current job: Registrar General's Social Class |  |  |  |  |  | X |  |  |  |  |  |  |  |  |
|  | f_jbrgsc_dv | Current job: Registrar General's Social Class |  |  |  |  |  |  | X |  |  |  |  |  |  |  |
|  | g_jbrgsc_dv | Current job: Registrar General's Social Class |  |  |  |  |  |  |  | X |  |  |  |  |  |  |
|  | h_jbrgsc_dv | Current job: Registrar General's Social Class |  |  |  |  |  |  |  |  | X |  |  |  |  |  |
|  | i_jbrgsc_dv | Current job: Registrar General's Social Class |  |  |  |  |  |  |  |  |  | X |  |  |  |  |
| **Income** | a_fimngrs_dv | total monthly personal income gross - 50-iles | Grouped into 50 sub-groups – 2% per group (total 100%) | X |  |  |  |  |  |  |  |  |  | X |  | |
|  | b_fimngrs_dv | total monthly personal income gross - 50-iles |  |  | X |  |  |  |  |  |  |  |  |  |  |  |
|  | c_fimngrs_dv | total monthly personal income gross - 50-iles |  |  |  | X |  |  |  |  |  |  |  |  |  |  |
|  | d_fimngrs_dv | total monthly personal income gross - 50-iles |  |  |  |  | X |  |  |  |  |  |  |  |  |  |
|  | e_fimngrs_dv | total monthly personal income gross - 50-iles |  |  |  |  |  | X |  |  |  |  |  |  |  |  |
|  | f_fimngrs_dv | total monthly personal income gross - 50-iles |  |  |  |  |  |  | X |  |  |  |  |  |  |  |
|  | g_fimngrs_dv | total monthly personal income gross - 50-iles |  |  |  |  |  |  |  | X |  |  |  |  |  |  |
|  | h_fimngrs_dv | total monthly personal income gross - 50-iles |  |  |  |  |  |  |  |  | X |  |  |  |  |  |
|  | i_fimngrs_dv | total monthly personal income gross - 50-iles |  |  |  |  |  |  |  |  |  | X |  |  |  |  |
| **Employment** | a_jbstat | Current economic activity | 0 = employed/retired/ maternity leave/ apprenticeship  1 = unemployed/education/ sick/in care/unpaid/gov training | X |  |  |  |  |  |  |  |  |  |  | X | |
|  | b_jbstat | Current economic activity |  |  | X |  |  |  |  |  |  |  |  |  |  |  |
|  | c_jbstat | Current economic activity |  |  |  | X |  |  |  |  |  |  |  |  |  |  |
|  | d_jbstat | Current economic activity |  |  |  |  | X |  |  |  |  |  |  |  |  |  |
|  | e_jbstat | Current economic activity |  |  |  |  |  | X |  |  |  |  |  |  |  |  |
|  | f_jbstat | Current economic activity |  |  |  |  |  |  | X |  |  |  |  |  |  |  |
|  | g_jbstat | Current economic activity |  |  |  |  |  |  |  | X |  |  |  |  |  |  |
|  | h_jbstat | Current economic activity |  |  |  |  |  |  |  |  | X |  |  |  |  |  |
|  | i_jbstat | Current economic activity |  |  |  |  |  |  |  |  |  | X |  |  |  |  |
| **Financial situation** | a_finnow | Subjective financial situation - current | 0 = Comfortable financially and just getting by financially  1 = Experiencing financial difficulties | X |  |  |  |  |  |  |  |  | X |  |  | |
|  | b_finnow | Subjective financial situation - current |  |  | X |  |  |  |  |  |  |  |  |  |  |  |
|  | c_finnow | Subjective financial situation - current |  |  |  | X |  |  |  |  |  |  |  |  |  |  |
|  | d_finnow | Subjective financial situation - current |  |  |  |  | X |  |  |  |  |  |  |  |  |  |
|  | e_finnow | Subjective financial situation - current |  |  |  |  |  | X |  |  |  |  |  |  |  |  |
|  | f_finnow | Subjective financial situation - current |  |  |  |  |  |  | X |  |  |  |  |  |  |  |
|  | g_finnow | Subjective financial situation - current |  |  |  |  |  |  |  | X |  |  |  |  |  |  |
|  | h_finnow | Subjective financial situation - current |  |  |  |  |  |  |  |  | X |  |  |  |  |  |
|  | i_finnow | Subjective financial situation - current |  |  |  |  |  |  |  |  |  | X |  |  |  |  |
| **Tenure** | a_hsownd | house owned or rented | 0 = owner/shared ownership /mortgaged  1 = rented/ rent-free | X |  |  |  |  |  |  |  |  |  |  | X | |
|  | b_hsownd | house owned or rented |  |  | X |  |  |  |  |  |  |  |  |  |  |  |
|  | c_hsownd | own accommodation |  |  |  | X |  |  |  |  |  |  |  |  |  |  |
|  | d_hsownd | house owned or rented |  |  |  |  | X |  |  |  |  |  |  |  |  |  |
|  | e_hsownd | own accommodation |  |  |  |  |  | X |  |  |  |  |  |  |  |  |
|  | f_hsownd | house owned or rented |  |  |  |  |  |  | X |  |  |  |  |  |  |  |
|  | g_hsownd | house owned or rented |  |  |  |  |  |  |  | X |  |  |  |  |  |  |
|  | h_hsownd | house owned or rented |  |  |  |  |  |  |  |  | X |  |  |  |  |  |
|  | i_hsownd | house owned or rented |  |  |  |  |  |  |  |  |  | X |  |  |  |  |
| **Marital Status** | a_mlstat | Present legal marital status | 0 = Married/in civil partnership  1 = single/separated/ divorced/ widowed | X |  |  |  |  |  |  |  |  | Cross-sectional | | | |
| **Depression** | a_hcond17 | Clinical depression | 0 = no  1 = yes | X |  |  |  |  |  |  |  |  | Only used for sensitivity analysis | | | |
|  | c_hcond17 | Clinical depression |  |  |  | X |  |  |  |  |  |  |  |  |  |  |
|  | e_hcond17 | Clinical depression |  |  |  |  |  | X |  |  |  |  |  |  |  |  |
|  | f_hcond17 | Clinical depression |  |  |  |  |  |  | X |  |  |  |  |  |  |  |
|  | g_hcond17 | Clinical depression |  |  |  |  |  |  |  | X |  |  |  |  |  |  |
|  | h_hcond17 | Clinical depression |  |  |  |  |  |  |  |  | X |  |  |  |  |  |
|  | i_hcond17 | Clinical depression |  |  |  |  |  |  |  |  |  | X |  |  |  |  |
| **Psychiatric problems** | bk_hlprxi | Received treatment for psychiatric problems | 0 = no  1 = yes | Individuals who previously participated in the British Household Panel study (now USoc) and received treatment for psychiatric problems during that time | | | | | | | | | Only used for sensitivity analysis | | | |
|  | bp_hlprxi | Received treatment for psychiatric problems |  |  |  |  |  |  |  |  |  |  |  |  |  |  |

Note: Any ‘N/A’, ‘Don’t know’ or ‘other’ answers have been excluded from the analysis. All variables were coded in STATA v12.1 (1).

# **References**

1. StataCorp. *Stata Statistical Software: Release 12*. College Station, TX: StataCorp LP; 2011.
