# Supplementary material for: Polygenic risk scores for schizophrenia and major depression are associated with socio-economic indicators of adversity in two British community samples
Source: Transl Psychiatry. 2022 Nov 14;12:477. doi: 10.1038/s41398-022-02247-8 (PMC9663827; doi:10.1038/s41398-022-02247-8)
Supplement: Supplementary file 2 — Supplementary_2_Descriptive_statistics [file 41398_2022_2247_MOESM2_ESM.docx]

**Supplementary Document 2 – Descriptive Statistics**

Table of Contents

[Supplementary Table 3: Descriptive statistics for each environmental exposure for NCDS 2](#_Toc99970912)

[Supplementary Table 4: Descriptive statistics for each environmental exposure for USoc 7](#_Toc99970913)

[References 15](#_Toc99970914)

# **Supplementary Table** **3**: **Descriptive statistics for each environmental exposure for NCDS**

| **Environment** | **Age** | **Total NCDS** | | | | **Genotyped (Combined)** | | | | **t-test/**  **chi-squared 95%CI** |
| --- | --- | --- | --- | --- | --- | --- | --- | --- | --- | --- |
|  |  | **Number of participants** | **% participants** | **Mean** | **(SD)** | **Number of participants** | **% participants** | **Mean** | **(SD)** |  |
| **Marital status as an adult** | 23 | 8,084 | 100 | N/A | N/A | 4,605 | 100 | N/A | N/A | χ²=2.046  p=.153 |
| Married/Co-habiting/In relationship |  | 4,117 | 50.93 |  |  | 2,406 | 52.25 |  |  |  |
| Not in relationship |  | 3,967 | 49.07 |  |  | 2,199 | 47.75 |  |  |  |
| **Marital status as an adult** | 33 | 7,800 | 100 | N/A | N/A | 4,442 | 100 | N/A | N/A | χ²=.956  p=.328 |
| Married/Co-habiting/In relationship |  | 6,781 | 86.94 |  |  | 3,889 | 87.55 |  |  |  |
| Not in relationship |  | 1,019 | 13.06 |  |  | 553 | 12.45 |  |  |  |
| **Marital status as an adult** | 42 | 9,087 | 100 | N/A | N/A | 5,129 | 100 | N/A | N/A | χ²= 1.980  p=.159 |
| Married/Co-habiting/In relationship |  | 7,443 | 81.91 |  |  | 4,260 | 83.06 |  |  |  |
| Not in relationship |  | 1,644 | 18.09 |  |  | 869 | 16.94 |  |  |  |
| **Marital status as an adult** | 46 | 7,480 | 100 | N/A | N/A | 4,280 | 100 | N/A | N/A | χ²=.329  p=.566 |
| Married/Co-habiting/In relationship |  | 6,309 | 84.34 |  |  | 3,627 84.74 | 3,627 84.74 |  |  |  |
| Not in relationship |  | 1,171 | 15.66 |  |  | 653 15.26 | 653 15.26 |  |  |  |
| **Marital status as an adult** | 50 | 8,181 | 100 | N/A | N/A | 4,661 | 100 | N/A | N/A | χ²=.979  p=.322 |
| Married/Co-habiting/In relationship |  | 6,592 | 80.58 |  |  | 3,789 | 81.29 |  |  |  |
| Not in relationship |  | 1,589 | 19.42 |  |  | 872 | 18.71 |  |  |  |
| **Marital status as an adult** | 55 | 7,664 | 100 | N/A | N/A | 4,422 | 100 | N/A | N/A | χ²=.339  p=.561 |
| Married/Co-habiting/In relationship |  | 6,126 | 79.93 |  |  | 3,554 | 80.37 |  |  |  |
| Not in relationship |  | 1,538 | 20.07 |  |  | 868 | 19.63 |  |  |  |
| **Number of rooms adult** | 23 | 7,884 | 100 | 2.70 | 0.92 | 4,483 | 100 | 2.69 | .91 | p=.622 |
| **Number of rooms adult** | 33 | 8,341 | 100 | 4.62 | 2.13 | 4,730 | 100 | 4.60 | 1.55 | p=.604 |
| **Number of rooms adult** | 42 | 9,053 | 100 | 3.05 | 0.92 | 5,113 | 100 | 3.07 | .90 | p=.407 |
| **Number of rooms adult** | 46 | 8,514 | 100 | 5.46 | 1.73 | 4,853 | 100 | 5.48 | 1.70 | p=.592 |
| **Number of rooms adult** | 50 | 8,161 | 100 | 5.51 | 1.83 | 4,651 | 100 | 5.40 | 1.80 | p=.671 |
| **Number of rooms adult** | 55 | 1,350 | 100 | 4.79 | 1.98 | 774 | 100 | 4.73 | 1.90 | p=.545 |
| **SES adult** | 23 | 6,364 | 100 | 3.01 | 0.77 | 3,627 | 100 | 3.00 | .76 | p=.500 |
| Class I |  | 207 | 3.25 |  |  | 121 | 3.34 |  |  |  |
| Class II |  | 1,036 | 16.28 |  |  | 586 | 16.16 |  |  |  |
| Class III |  | 3,815 | 59.95 |  |  | 2,201 | 60.68 |  |  |  |
| Class IV |  | 1,113 | 17.49 |  |  | 621 | 17.12 |  |  |  |
| Class V |  | 193 | 3.03 |  |  | 98 | 2.70 |  |  |  |
| **SES adult** | 33 | 7,808 | 100 | 3.26 | 0.88 | 4,414 | 100 | 3.21 | .88 | p=.458 |
| Class I |  | 255 | 3.27 |  |  | 149 | 3.38 |  |  |  |
| Class II |  | 1,130 | 14.47 |  |  | 637 | 14.43 |  |  |  |
| Class III |  | 3,455 | 44.25 |  |  | 1,988 | 45.04 |  |  |  |
| Class IV |  | 2,534 | 32.45 |  |  | 1,403 | 31.79 |  |  |  |
| Class V |  | 434 | 5.56 |  |  | 237 | 5.37 |  |  |  |
| **SES adult** | 42 | 7,810 | 100 | 3.32 | 0.87 | 4,437 | 100 | 3.32 | .86 | p=.929 |
| Class I |  | 239 | 3.06 |  |  | 139 | 3.13 |  |  |  |
| Class II |  | 922 | 11.81 |  |  | 503 | 11.34 |  |  |  |
| Class III |  | 3,196 | 40.92 |  |  | 1,838 | 41.42 |  |  |  |
| Class IV |  | 3,004 | 38.46 |  |  | 1,718 | 38.72 |  |  |  |
| Class V |  | 449 | 5.75 |  |  | 239 | 5.39 |  |  |  |
| **SES adult** | 46 | 7,465 | 100 | 3.38 | 0.84 | 4,280 | 100 | 3.37 | .83 | p=.313 |
| Class I |  | 164 | 2.20 |  |  | 87 | 2.03 |  |  |  |
| Class II |  | 818 | 10.96 |  |  | 475 | 11.10 |  |  |  |
| Class III |  | 2,912 | 39.01 |  |  | 1,729 | 40.40 |  |  |  |
| Class IV |  | 3,121 | 41.81 |  |  | 1,750 | 40.89 |  |  |  |
| Class V |  | 450 | 6.03 |  |  | 239 | 5.58 |  |  |  |
| **SES adult** | 50 | 7,069 | 100 | 3.39 | 0.85 | 4,075 | 100 | 3.39 | .84 | p=.742 |
| Class I |  | 159 | 2.25 |  |  | 90 | 2.21 |  |  |  |
| Class II |  | 780 | 11.03 |  |  | 438 | 10.75 |  |  |  |
| Class III |  | 2,698 | 38.17 |  |  | 1,581 | 38.80 |  |  |  |
| Class IV |  | 2,983 | 42.20 |  |  | 1,732 | 42.50 |  |  |  |
| Class V |  | 449 | 6.35 |  |  | 234 | 5.74 |  |  |  |
| **SES adult** | 55 | 6,056 | 100 | 3.39 | 0.86 | 3,518 | 100 | 3.39 | .85 | p=.847 |
| Class I |  | 134 | 2.21 |  |  | 75 | 2.13 |  |  |  |
| Class II |  | 706 | 11.66 |  |  | 394 | 11.20 |  |  |  |
| Class III |  | 2,264 | 37.38 |  |  | 1,355 | 38.52 |  |  |  |
| Class IV |  | 2,547 | 42.06 |  |  | 1,472 | 41.84 |  |  |  |
| Class V |  | 405 | 6.69 |  |  | 222 | 6.31 |  |  |  |
| **Tenure adult** | 23 | 4,615 | 100 | N/A | N/A | 2,679 | 100 | N/A | N/A | χ²=.0009  p=.976 |
| Owns/part-owns |  | 2,522 | 54.65 |  |  | 1,465 | 54.68 |  |  |  |
| rents |  | 2,093 | 45.35 |  |  | 1,214 | 45.32 |  |  |  |
| **Tenure adult** | 33 | 7,606 | 100 | N/A | N/A | 4,338 | 100 | N/A | N/A | χ²=.481  p=.488 |
| Owns/part-owns |  | 6,290 | 82.70 |  |  | 3,609 | 83.20 |  |  |  |
| rents |  | 1,316 | 17.30 |  |  | 729 | 16.80 |  |  |  |
| **Tenure adult** | 42 | 8,806 | 100 | N/A | N/A | 4,986 | 100 | N/A | N/A | χ²=.018  p=.892 |
| Owns/part-owns |  | 7,534 | 85.56 |  |  | 4,270 | 85.64 |  |  |  |
| rents |  | 1,272 | 14.44 |  |  | 716 | 14.36 |  |  |  |
| **Tenure adult** | 46 | 8,460 | 100 | N/A | N/A | 4,826 | 100 | N/A | N/A | χ²=.498  p=.480 |
| Owns/part-owns |  | 7,399 | 87.46 |  |  | 4,241 | 87.88 |  |  |  |
| rents |  | 1,061 | 12.54 |  |  | 585 | 12.12 |  |  |  |
| **Tenure adult** | 50 | 8,114 | 100 | N/A | N/A | 4,623 | 100 | N/A | N/A | χ²=1.142  p=.285 |
| Owns/part-owns |  | 7,028 | 86.62 |  |  | 4,035 | 87.28 |  |  |  |
| rents |  | 1,086 | 13.38 |  |  | 588 | 12.72 |  |  |  |
| **Tenure adult** | 55 | 4,507 | 100 | N/A | N/A | 2,533 | 100 | N/A | N/A | χ²=.012  p=.915 |
| Owns/part-owns |  | 3,609 | 80.08 |  |  | 2,031 | 80.18 |  |  |  |
| rents |  | 898 | 19.92 |  |  | 502 | 19.82 |  |  |  |
| **Smoking adult** | 23 | 8,085 | 100 | N/A | N/A | 4,606 | 100 | N/A | N/A | χ²=.841  p=.359 |
| Non-smoker |  | 2,526 | 31.24 |  |  | 1,403 30.46 | 1,403 30.46 |  |  |  |
| Smoker |  | 5,559 | 68.76 |  |  | 3,203 69.54 | 3,203 69.54 |  |  |  |
| **Smoking adult** | 42 | 9,079 | 100 | N/A | N/A | 5,127 | 100 | N/A | N/A | χ²=.579  p=.447 |
| Non-smoker |  | 4,156 | 45.78 |  |  | 2,313 | 45.11 |  |  |  |
| Smoker |  | 4,923 | 54.22 |  |  | 2,814 | 54.89 |  |  |  |
| **Smoking adult** | 50 | 8,189 | 100 | N/A | N/A | 4,667 | 100 | N/A | N/A | χ²=.343  p=.558 |
| Non-smoker |  | 3,855 | 47.08 |  |  | 2,172 | 46.54 |  |  |  |
| Smoker |  | 4,334 | 52.92 |  |  | 2,495 | 53.46 |  |  |  |
| **Employment adult** | 23 | 3,403 | 100 | N/A | N/A | 1,898 | 100 | N/A | N/A | χ²=.125  p=.724 |
| Employed |  | 2,800 | 82.28 |  |  | 1,569 | 82.67 |  |  |  |
| Unemployed/disabled/in education |  | 603 | 17.72 |  |  | 329 | 17.33 |  |  |  |
| **Employment adult** | 42 | 9,011 | 100 | N/A | N/A | 5,091 | 100 | N/A | N/A | χ²=.828  p=.363 |
| Employed |  | 7,839 | 86.99 |  |  | 4,456 | 87.53 |  |  |  |
| Unemployed/disabled/in education |  | 1,172 | 13.01 |  |  | 635 | 12.47 |  |  |  |
| **Employment adult** | 50 | 8,047 | 100 | N/A | N/A | 4,599 | 100 | N/A | N/A | χ²=1.593  p=.207 |
| Employed |  | 7,091 | 88.12 |  |  | 4,087 | 88.87 |  |  |  |
| Unemployed/disabled/in education |  | 956 | 11.88 |  |  | 512 | 11.13 |  |  |  |
| **Employment adult** | 55 | 7,258 | 100 | N/A | N/A | 4,198 | 100 | N/A | N/A | χ²=.743  p=.389 |
| Employed |  | 6,234 | 85.89 |  |  | 3,630 | 86.47 |  |  |  |
| Unemployed/disabled/in education |  | 1,024 | 14.11 |  |  | 568 | 13.53 |  |  |  |

Notes: NCDS Total = refers to all individuals who submitted the biomedical survey at the age of 44 and for whom we have phenotype data for. All descriptive statistics were calculated using STATA v12.1 (1).

# **Supplementary Table** **4**: **Descriptive statistics for each environmental exposure for USoc**

| **Environment** | **Wave** | **Total Understanding Society** | | | | **Genotyped** | | | | **t-test/**  **chi-squared 95%CI** |
| --- | --- | --- | --- | --- | --- | --- | --- | --- | --- | --- |
|  |  | **Number of participants** | **% participants** | **Mean** | **(SD)** | **Number of participants** | **% participants** | **Mean** | **(SD)** |  |
| **Marital Status** | 1 | 6,844 | 100 | N/A | N/A | 5,272 | 100 | N/A | N/A | χ²= 25.70  p = <0.0001 |
| Married, in relationship |  | 3,943 | 57.61 |  |  | 2,794 | 53.00 |  |  |  |
| Single, divorced, separated, widowed |  | 2,901 | 42.39 |  |  | 2,478 | 47.00 |  |  |  |
| **Alcohol consumption** | 2 | 6,271 | 100 | 3.10 | 1.93 | 4,606 | 100 | 3.12 | 1.96 | p=.448 |
| **Alcohol consumption** | 5 | 5,182 | 100 | 3.17 | 1.90 | 3,790 | 100 | 3.17 | 1.92 | p=.864 |
| **Income** | 1 | 6,939 | 100 | 24.75 | 14.85 | 5,335 | 100 | 24.95 | 14.67 | p=.461 |
| **Income** | 2 | 9,768 | 100 | 24.93 | 14.76 | 7,279 | 100 | 25.04 | 14.53 | p=.617 |
| **Income** | 3 | 9,508 | 100 | 25.02 | 14.70 | 7,066 | 100 | 25.13 | 14.50 | p=.631 |
| **Income** | 4 | 9,080 | 100 | 25.05 | 14.69 | 6,732 | 100 | 25.13 | 14.50 | p=.734 |
| **Income** | 5 | 8,709 | 100 | 25.04 | 14.69 | 6,448 | 100 | 25.15 | 14.54 | p=.633 |
| **Income** | 6 | 8,033 | 100 | 25.15 | 14.64 | 5,938 | 100 | 25.27 | 14.48 | p=.615 |
| **Income** | 7 | 7,650 | 100 | 25.12 | 14.65 | 5,666 | 100 | 25.26 | 14.46 | p=.594 |
| **Income** | 8 | 7,292 | 100 | 25.16 | 14.62 | 5,387 | 100 | 25.28 | 14.42 | p=.665 |
| **Income** | 9 | 6,829 | 100 | 25.05 | 14.69 | 5,038 | 100 | 25.09 | 14.47 | p=.899 |
| **Rooms** | 1 | 6,986 | 100 | 2.99 | .98 | 5,359 | 100 | 2.93 | 0.99 | p=.0006 |
| **Rooms** | 2 | 9,809 | 100 | 2.30 | .98 | 7,307 | 100 | 2.94 | 0.99 | p=.0001 |
| **Rooms** | 3 | 9,548 | 100 | 3.01 | .98 | 7.088 | 100 | 2.95 | .98 | p=.0001 |
| **Rooms** | 4 | 9,061 | 100 | 3.02 | .98 | 6,722 | 100 | 2.96 | .98 | p=.0001 |
| **Rooms** | 5 | 8,710 | 100 | 3.03 | .97 | 6,442 | 100 | 2.97 | .98 | p=.0002 |
| **Rooms** | 6 | 8,072 | 100 | 3.04 | .98 | 5,960 | 100 | 2.98 | 0.99 | p=.0001 |
| **Rooms** | 7 | 7,727 | 100 | 3.05 | .99 | 5,719 | 100 | 3.00 | 0.99 | p=.0002 |
| **Rooms** | 8 | 7,377 | 100 | 3.05 | .99 | 5,435 | 100 | 2.99 | .99 | p=.0007 |
| **Rooms** | 9 | 6,889 | 100 | 3.08 | .99 | 5,055 | 100 | 3.01 | 1.00 | p=.001 |
| **SES** | 1 | 3,938 | 100 | 3.29 | .90 | 2,963 | 100 | 3.31 | .89 | p=.365 |
| Class I |  | 151 | 3.83 |  |  | 100 | 3.37 |  |  |  |
| Class II |  | 480 | 12.19 |  |  | 363 | 12.25 |  |  |  |
| Class III |  | 1,614 | 40.99 |  |  | 1,198 | 40.43 |  |  |  |
| Class IV |  | 1,452 | 36.87 |  |  | 1,116 | 37.66 |  |  |  |
| Class V |  | 241 | 6.12 |  |  | 186 | 6.28 |  |  |  |
| **SES** | 2 | 5,547 | 100 | 3.29 | .88 | 4,019 | 100 | 3.30 | .87 | p=.474 |
| Class I |  | 183 | 3.3 |  |  | 118 | 2.94 |  |  |  |
| Class II |  | 717 | 12.93 |  |  | 525 | 13.06 |  |  |  |
| Class III |  | 2,283 | 41.16 |  |  | 1,636 | 40.71 |  |  |  |
| Class IV |  | 2,058 | 37.1 |  |  | 1,517 | 37.75 |  |  |  |
| Class V |  | 306 | 5.52 |  |  | 223 | 5.55 |  |  |  |
| **SES** | 3 | 5,331 | 100 | 3.29 | .87 | 3,842 | 100 | 3.30 | .87 | p=.542 |
| Class I |  | 168 | 3.15 |  |  | 110 | 2.86 |  |  |  |
| Class II |  | 685 | 12.85 |  |  | 497 | 12.94 |  |  |  |
| Class III |  | 2,223 | 41.7 |  |  | 1,592 | 41.44 |  |  |  |
| Class IV |  | 1,967 | 36.9 |  |  | 1,429 | 37.19 |  |  |  |
| Class V |  | 288 | 5.4 |  |  | 214 | 5.57 |  |  |  |
| **SES** | 4 | 5,063 | 100 | 3.29 | .87 | 3,640 | 100 | 3.30 | .87 | p=.819 |
| Class I |  | 150 | 2.96 |  |  | 106 | 2.91 |  |  |  |
| Class II |  | 662 | 13.08 |  |  | 481 | 13.21 |  |  |  |
| Class III |  | 2,082 | 41.12 |  |  | 1,485 | 40.80 |  |  |  |
| Class IV |  | 1,886 | 37.25 |  |  | 1,356 | 37.25 |  |  |  |
| Class V |  | 283 | 5.59 |  |  | 212 | 5.82 |  |  |  |
| **SES** | 5 | 4,862 | 100 | 3.30 | .87 | 3,477 | 100 | 3.31 | .88 | p=.641 |
| Class I |  | 140 | 2.88 |  |  | 98 | 2.82 |  |  |  |
| Class II |  | 648 | 13.33 |  |  | 460 | 13.23 |  |  |  |
| Class III |  | 1,967 | 40.46 |  |  | 1,401 | 40.29 |  |  |  |
| Class IV |  | 1,831 | 37.66 |  |  | 1,308 | 37.62 |  |  |  |
| Class V |  | 276 | 5.68 |  |  | 210 | 6.04 |  |  |  |
| **SES** | 6 | 4,404 | 100 | 3.33 | .86 | 3,150 | 100 | 3.33 | .86 | p=.962 |
| Class I |  | 111 | 2.52 |  |  | 79 | 2.51 |  |  |  |
| Class II |  | 555 | 12.6 |  |  | 401 | 12.73 |  |  |  |
| Class III |  | 1,758 | 39.92 |  |  | 1,249 | 39.65 |  |  |  |
| Class IV |  | 1,736 | 39.42 |  |  | 1,245 | 39.52 |  |  |  |
| Class V |  | 244 | 5.54 |  |  | 176 | 5.59 |  |  |  |
| **SES** | 7 | 4,135 | 100 | 3.34 | .86 | 2,975 | 100 | 3.34 | .86 | p=.927 |
| Class I |  | 107 | 2.59 |  |  | 73 | 2.45 |  |  |  |
| Class II |  | 513 | 12.41 |  |  | 376 12.64 | 376 12.64 |  |  |  |
| Class III |  | 1,640 | 39.66 |  |  | 1,177 39.56 | 1,177 39.56 |  |  |  |
| Class IV |  | 1,634 | 39.52 |  |  | 1,171 39.36 | 1,171 39.36 |  |  |  |
| Class V |  | 241 | 5.83 |  |  | 178 5.98 | 178 5.98 |  |  |  |
| **SES** | 8 | 3,818 | 100 | 3.34 | .87 | 2,754 | 100 | 3.35 | .86 | p=.824 |
| Class I |  | 100 | 2.62 |  |  | 64 | 2.32 |  |  |  |
| Class II |  | 470 | 12.31 |  |  | 344 | 12.49 |  |  |  |
| Class III |  | 1,507 | 39.47 |  |  | 1,090 | 39.58 |  |  |  |
| Class IV |  | 1,505 | 39.42 |  |  | 1,084 | 39.36 |  |  |  |
| Class V |  | 236 | 6.18 |  |  | 172 | 6.25 |  |  |  |
| **SES** | 9 | 3,449 | 100 | 3.35 | .87 | 2,485 | 100 | 3.35 | .87 | p=.886 |
| Class I |  | 87 | 2.52 |  |  | 59 | 2.37 |  |  |  |
| Class II |  | 427 | 12.38 |  |  | 314 | 12.64 |  |  |  |
| Class III |  | 1,331 | 38.59 |  |  | 968 | 38.95 |  |  |  |
| Class IV |  | 1,391 | 40.33 |  |  | 988 | 39.76 |  |  |  |
| Class V |  | 213 | 6.18 |  |  | 156 | 6.28 |  |  |  |
| **Financial Issues** | 1 | 6,839 | 100 | N/A | N/A | 5,268 | 100 | N/A | N/A | χ²= 0.060  p =.807 |
| Financially comfortable |  | 6,183 | 90.41 |  |  | 4,753 | 90.22 |  |  |  |
| Financial issues |  | 656 | 9.59 |  |  | 515 | 9.78 |  |  |  |
| **Financial Issues** | 2 | 9,740 | 100 | N/A | N/A | 7,258 | 100 | N/A | N/A | χ²=.989  p =.320 |
| Financially comfortable |  | 8,893 | 91.3 |  |  | 6,595 | 90.87 |  |  |  |
| Financial issues |  | 847 | 8.7 |  |  | 663 | 9.13 |  |  |  |
| **Financial Issues** | 3 | 9,429 | 100 | N/A | N/A | 7,014 | 0 | N/A | N/A | χ²=.291  p =.442 |
| Financially comfortable |  | 8,625 | 91 |  |  | 6,392 | 91.13 |  |  |  |
| Financial issues |  | 804 | 8.53 |  |  | 622 | 8.87 |  |  |  |
| **Financial Issues** | 4 | 8,960 | 100 | N/A | N/A | 6,658 | 100 | N/A | N/A | χ²=.796  p =.372 |
| Financially comfortable |  | 8,255 | 92.13 |  |  | 6,108 | 91.74 |  |  |  |
| Financial issues |  | 705 | 7.87 |  |  | 550 | 8.26 |  |  |  |
| **Financial Issues** | 5 | 8,610 | 100 | N/A | N/A | 6,387 | 100 | N/A | N/A | χ²=.296  p =.587 |
| Financially comfortable |  | 8,019 | 93 |  |  | 5,934 | 92.91 |  |  |  |
| Financial issues |  | 591 | 6.86 |  |  | 453 | 7.09 |  |  |  |
| **Financial Issues** | 6 | 7,921 | 100 | N/A | N/A | 5,861 | 100 | N/A | N/A | χ²=1.02  p =.317 |
| Financially comfortable |  | 7,562 | 95.47 |  |  | 5,574 | 95.10 |  |  |  |
| Financial issues |  | 359 | 4.53 |  |  | 287 | 4.90 |  |  |  |
| **Financial Issues** | 7 | 7,549 | 100 | N/A | N/A | 5,602 | 100 | N/A | N/A | χ²=.210  p =.647 |
| Financially comfortable |  | 7,210 | 95.51 |  |  | 5,341 | 95.34 |  |  |  |
| Financial issues |  | 339 | 4.49 |  |  | 261 | 4.66 |  |  |  |
| **Financial Issues** | 8 | 7,232 | 100 | N/A | N/A | 5,346 | 100 | N/A | N/A | χ²=.646  p =.421 |
| Financially comfortable |  | 6,867 | 94.95 |  |  | 5,059 | 94.63 |  |  |  |
| Financial issues |  | 365 | 5.05 |  |  | 287 | 5.37 |  |  |  |
| **Financial Issues** | 9 | 6,803 | 100 | N/A | N/A | 5,016 | 100 | N/A | N/A | χ²=1.19  p =.275 |
| Financially comfortable |  | 6,466 | 95.05 |  |  | 4,745 | 94.60 |  |  |  |
| Financial issues |  | 337 | 4.95 |  |  | 271 | 5.40 |  |  |  |
| **Tenure** | 1 | 6,953 | 100 | N/A | N/A | 5,331 | 100 | N/A | N/A | χ²=2.66  p =.103 |
| Owner, mortgaged, shared owner |  | 5,358 | 77.06 |  |  | 4,041 | 75.80 |  |  |  |
| Rent, rent-free |  | 1,595 | 23 |  |  | 1,290 | 24.20 |  |  |  |
| **Tenure** | 2 | 9,786 | 100 | N/A | N/A | 7,287 | 100 | N/A | N/A | χ²=3.172  p =.075 |
| Owner, mortgaged, shared owner |  | 7,608 | 77.74 |  |  | 5,581 | 76.59 |  |  |  |
| Rent, rent-free |  | 2,178 | 22.26 |  |  | 1,706 | 23.41 |  |  |  |
| **Tenure** | 3 | 9,532 | 100 | N/A | N/A | 7,075 | 100 | N/A | N/A | χ²=2.730  p =.098 |
| Owner, mortgaged, shared owner |  | 7,439 | 78.04 |  |  | 5,445 | 76.96 |  |  |  |
| Rent, rent-free |  | 2,093 | 22 |  |  | 1,630 | 23.04 |  |  |  |
| **Tenure** | 4 | 9,040 | 100 | N/A | N/A | 6,703 | 100 | N/A | N/A | χ²=2.649  p =.104 |
| Owner, mortgaged, shared owner |  | 7,095 | 78.48 |  |  | 5,188 | 77.40 |  |  |  |
| Rent, rent-free |  | 1,945 | 21.52 |  |  | 1,515 | 22.60 |  |  |  |
| **Tenure** | 5 | 8,696 | 100 | N/A | N/A | 6,431 | 100 | N/A | N/A | χ²=2.861  p =.091 |
| Owner, mortgaged, shared owner |  | 6,834 | 79 |  |  | 4,980 | 77.44 |  |  |  |
| Rent, rent-free |  | 1,862 | 21.41 |  |  | 1,451 | 22.56 |  |  |  |
| **Tenure** | 6 | 8,056 | 100 | N/A | N/A | 5,949 | 100 | N/A | N/A | χ²=3.149  p =.076 |
| Owner, mortgaged, shared owner |  | 6,398 | 79.42 |  |  | 4,651 | 78.18 |  |  |  |
| Rent, rent-free |  | 1,658 | 20.58 |  |  | 1,298 | 21.82 |  |  |  |
| **Tenure** | 7 | 7,706 | 100 | N/A | N/A | 5,703 | 100 | N/A | N/A | χ²=2.744  p =.098 |
| Owner, mortgaged, shared owner |  | 6,132 | 80 |  |  | 4,471 | 78.40 |  |  |  |
| Rent, rent-free |  | 1,574 | 20 |  |  | 1,232 | 21.60 |  |  |  |
| **Tenure** | 8 | 7,329 | 100 | N/A | N/A | 5,397 | 100 | N/A | N/A | χ²=2.072  p =.150 |
| Owner, mortgaged, shared owner |  | 5,860 | 80 |  |  | 4,259 | 78.91 |  |  |  |
| Rent, rent-free |  | 1,469 | 20 |  |  | 1,138 | 21.09 |  |  |  |
| **Tenure** | 9 | 6,850 | 100 | N/A | N/A | 5,028 | 100 | N/A | N/A | χ²=1.763  p =.184 |
| Owner, mortgaged, shared owner |  | 5,561 | 81 |  |  | 4,033 | 80.21 |  |  |  |
| Rent, rent-free |  | 1,289 | 18.82 |  |  | 995 | 19.79 |  |  |  |
| **Employment** | 1 | 6,903 | 100 | N/A | N/A | 5,306 | 100 | N/A | N/A | χ²=.062  p =.803 |
| employed/retired/maternity leave/apprenticeship |  | 5,854 | 85 |  |  | 4,491 | 84.64 |  |  |  |
| unemployed/education/sick/in care/unpaid/gov training |  | 1,049 | 15 |  |  | 815 | 15.36 |  |  |  |
| **Employment** | 2 | 9,737 | 100 | N/A | N/A | 7,253 | 100 | N/A | N/A | χ²=<.0.0001  p =.978 |
| employed/retired/maternity leave/apprenticeship |  | 8,302 | 85 |  |  | 6,183 | 85.25 |  |  |  |
| unemployed/education/sick/in care/unpaid/gov training |  | 1,435 | 15 |  |  | 1,070 | 14.75 |  |  |  |
| **Employment** | 3 | 9,477 | 100 | N/A | N/A | 7,040 | 100 | N/A | N/A | χ²=.004  p =.950 |
| employed/retired/maternity leave/apprenticeship |  | 8,199 | 87 |  |  | 6,093 | 86.55 |  |  |  |
| unemployed/education/sick/in care/unpaid/gov training |  | 1,278 | 13.49 |  |  | 947 | 13.45 |  |  |  |
| **Employment** | 4 | 9,037 | 100 | N/A | N/A | 6,700 | 100 | N/A | N/A | χ²=.086  p =.770 |
| employed/retired/maternity leave/apprenticeship |  | 7,978 | 88.28 |  |  | 5,925 | 88.43 |  |  |  |
| unemployed/education/sick/in care/unpaid/gov training |  | 1,059 | 11.72 |  |  | 775 | 11.57 |  |  |  |
| **Employment** | 5 | 8,672 | 100 | N/A | N/A | 6,419 | 100 | N/A | N/A | χ²=.046  p =.830 |
| employed/retired/maternity leave/apprenticeship |  | 7,735 | 89 |  |  | 5,713 | 89.00 |  |  |  |
| unemployed/education/sick/in care/unpaid/gov training |  | 937 | 11 |  |  | 706 | 11.00 |  |  |  |
| **Employment** | 6 | 7,996 | 100 | N/A | N/A | 5,913 | 100 | N/A | N/A | χ²=.003  p =.957 |
| employed/retired/maternity leave/apprenticeship |  | 7,276 | 91 |  |  | 5,379 | 90.97 |  |  |  |
| unemployed/education/sick/in care/unpaid/gov training |  | 720 | 9 |  |  | 534 | 9.03 |  |  |  |
| **Employment** | 7 | 7,627 | 100 | N/A | N/A | 5,650 | 100 | N/A | N/A | χ²=.177  p =.674 |
| employed/retired/maternity leave/apprenticeship |  | 6,996 | 91.73 |  |  | 5,171 | 91.52 |  |  |  |
| unemployed/education/sick/in care/unpaid/gov training |  | 631 | 8.27 |  |  | 479 | 8.48 |  |  |  |
| **Employment** | 8 | 7,252 | 100 | N/A | N/A | 5,366 | 100 | N/A | N/A | χ²=.139  p =.709 |
| employed/retired/maternity leave/apprenticeship |  | 6,676 | 92.06 |  |  | 4,930 | 91.87 |  |  |  |
| unemployed/education/sick/in care/unpaid/gov training |  | 576 | 7.94 |  |  | 436 | 8.13 |  |  |  |
| **Employment** | 9 | 6,791 | 100 | N/A | N/A | 5,015 | 100 | N/A | N/A | χ²=.221  p =.638 |
| employed/retired/maternity leave/apprenticeship |  | 6,276 | 92 |  |  | 4,623 | 92.18 |  |  |  |
| unemployed/education/sick/in care/unpaid/gov training |  | 515 | 7.58 |  |  | 392 | 7.82 |  |  |  |
| **Education** | 1 | 6,112 | 100 | N/A | N/A | 4,691 | 100 | N/A | N/A | χ²=.046  p =.830 |
| A-Level and above |  | 3,637 | 59.51 |  |  | 2,801 | 59.71 |  |  |  |
| GCSE and below |  | 2,475 | 40.49 |  |  | 1,890 | 40.29 |  |  |  |
| **Education** | 2 | 8,609 | 100 | N/A | N/A | 6,404 | 100 | N/A | N/A | χ²=<.0001  p =.996 |
| A-Level and above |  | 5,178 | 60.15 |  |  | 3,852 | 60.15 |  |  |  |
| GCSE and below |  | 3,431 | 39.85 |  |  | 2,552 | 39.85 |  |  |  |
| **Education** | 3 | 8,401 | 100 | N/A | N/A | 6,221 | 100 | N/A | N/A | χ²=.006  p =.936 |
| A-Level and above |  | 5,102 | 60.73 |  |  | 3,774 | 60.67 |  |  |  |
| GCSE and below |  | 3,299 | 39.27 |  |  | 2,447 | 39.33 |  |  |  |
| **Education** | 4 | 8,076 | 100 | N/A | N/A | 5,960 | 100 | N/A | N/A | χ²=.055  p =.815 |
| A-Level and above |  | 5,032 | 62.31 |  |  | 3,702 | 62.11 |  |  |  |
| GCSE and below |  | 3,044 | 37.69 |  |  | 2,258 | 37.89 |  |  |  |
| **Education** | 5 | 7,749 | 100 | N/A | N/A | 5,710 | 100 | N/A | N/A | χ²=.688  p =.407 |
| A-Level and above |  | 4,880 | 62.98 |  |  | 3,590 | 62.87 |  |  |  |
| GCSE and below |  | 2,869 | 37.02 |  |  | 2,120 | 37.13 |  |  |  |
| **Education** | 6 | 7,172 | 100 | N/A | N/A | 5,273 | 100 | N/A | N/A | χ²=.125  p =.724 |
| A-Level and above |  | 4,584 | 63.92 |  |  | 3,354 | 63.61 |  |  |  |
| GCSE and below |  | 2,588 | 36.08 |  |  | 1,919 | 36.39 |  |  |  |
| **Education** | 7 | 6,854 | 100 | N/A | N/A | 5,052 | 100 | N/A | N/A | χ²=.042  p =.837 |
| A-Level and above |  | 4,415 | 64.41 |  |  | 3,245 | 64.23 |  |  |  |
| GCSE and below |  | 2,439 | 36 |  |  | 1,807 | 35.77 |  |  |  |
| **Education** | 8 | 6,538 | 100 | N/A | N/A | 4,812 | 100 | N/A | N/A | χ²=.103  p =.748 |
| A-Level and above |  | 4,250 | 65 |  |  | 3,114 | 64.71 |  |  |  |
| GCSE and below |  | 2,288 | 35 |  |  | 1,698 | 35.29 |  |  |  |
| **Education** | 9 | 6,133 | 100 | N/A | N/A | 4,506 | 100 | N/A | N/A | χ²=.057  p =.812 |
| A-Level and above |  | 4,022 | 65.58 |  |  | 2,945 | 65.36 |  |  |  |
| GCSE and below |  | 2,111 | 34.42 |  |  | 1,561 | 34.64 |  |  |  |

Notes: All descriptive statistics were calculated using STATA v12.1 (1).

# **References**

1. StataCorp. *Stata Statistical Software: Release 12*. College Station, TX: StataCorp LP; 2011.
