# Supplementary material for: Polygenic risk scores for schizophrenia and major depression are associated with socio-economic indicators of adversity in two British community samples
Source: Transl Psychiatry. 2022 Nov 14;12:477. doi: 10.1038/s41398-022-02247-8 (PMC9663827; doi:10.1038/s41398-022-02247-8)
Supplement: Supplementary file 3 — Supplementary_3_Genetic_QC [file 41398_2022_2247_MOESM3_ESM.docx]

**Supplementary Document**

Table of Contents

[Supplementary Table 5: Power Calculation for NCDS 2](#_Toc99010915)

[Supplementary Table 6: Dataset process flow - NCDS 2](#_Toc99010916)

[Supplementary Table 7: Pre-Imputation QC steps for each genotype group for NCDS 3](#_Toc99010917)

[Supplementary Fig 3: WTCCC1 ancestry grouping (NCDS) 4](#_Toc99010918)

[Supplementary Table 8: Imputation for NCDS 5](#_Toc99010919)

[Supplementary Table 9: Post-Imputation QC for NCDS 5](#_Toc99010920)

[Supplementary Table 10: Combining of WTCCC2 , T1DGC and WTCCC1 genetic information into a single dataset - NCDS 7](#_Toc99010921)

[Supplementary Table 11A: Creation of PRS - NCDS 8](#_Toc99010922)

[Supplementary Table 11B: PRS Base file summary statistics - NCDS 8](#_Toc99010923)

[Supplementary Table 12: Principal Components – Combined NCDS 8](#_Toc99010924)

[Supplementary Fig 4: Principal Components – Combined NCDS 9](#_Toc99010925)

[Supplementary Table 13: Merging of genotype, phenotype, eigenvector and PRS information (separate merges for SCZ and MDD) – NCDS 9](#_Toc99010926)

[Supplementary Table 14: Power Calculation for USoc 10](#_Toc99010927)

[Supplementary Table 15: for USoc dataset process flow 10](#_Toc99010928)

[Supplementary Table 16: Pre-Imputation QC steps for USoc 11](#_Toc99010929)

[Supplementary Table 17: Principal Components Selection for USoc 11](#_Toc99010930)

[Supplementary Fig 7: Principal Components Plotting for USoc 12](#_Toc99010931)

[Supplementary Fig 8: Ancestry grouping for USoc 12](#_Toc99010932)

[Supplementary Table 18: Imputation for USoc 13](#_Toc99010933)

[Supplementary Table 19: Post-Imputation QC for USoc 13](#_Toc99010934)

[Supplementary Table 20A: Creation of PRS for USoc 15](#_Toc99010935)

[Supplementary Table 20B: PRS Base file summary statistics for USoc 15](#_Toc99010936)

[Supplementary Table 22A: Random selection of one individual from each household for USoc 16](#_Toc99010937)

[Supplementary Table 22B: Age of individuals for USoc 17](#_Toc99010938)

[References: 17](#_Toc99010939)

# **Supplementary Table 5:** Power Calculation for NCDS

| Power calculation model used | F-test (Fixed linear regression) |  |
| --- | --- | --- |
| Power | 0.8 |  |
| Effect size | 0.005 |  |
| Error rate | 0.0042 (=0.05/12 environments tested) |  |
| Number of tested predictors | 1 |  |
| **Minimum sample size required** | **2,749** |  |

Note: Minimum sampe size calculated using G*Power 3.1.9.6 (1). Effect sizes are based on similar rGE studies (2)

# **Supplementary Table 6:** Dataset process flow - NCDS

WTCCC1

TD1GC

WTCCC2

Pre-imputation genetic

QC (*T7, Fig 1-3)*

WTCCC1

T1DGC

WTCCC2

Imputation of genetic datasets

(*T8*)

WTCCC1

T1DGC

WTCCC2

Post-imputation genetic

QC (*T9*)

Combined WTCCC2/T1DGC/WTCCC1

Combining

of genetic datasets (*T10)*

Creation of individual

Combined WTCCC2/T1DGC/WTCCC1

PRS scores & (*T11A&B)*

& Principal components *(T12 & Fig 4)*

Merging of genetic, phenotype,

Combined WTCCC2/T1DGC/WTCCC1

eigenvector & PRS information

*(T13)*

Note: WTCCC2 = The Wellcome Trust Case Control Consortium 2 - 2,922 individuals (controls) genotyped using the Illumina 1.2M array (https://www.ncbi.nlm.nih.gov/projects/gap/cgi-bin/study.cgi?study_id=EGAS00000000028) , T1DGC = Type 1 Diabetes Genetics Consortium - 2,592 individuals (controls), genotyped on Infinium Humanhap 550k v3 chips (3), WTCCC1 = The Wellcome Trust Case Control Consortium - 1,502 individuals (controls) genotyped using the Affymetix 500k 1.2M (4), PRS = Polygenic Risk Scores

# **Supplementary Table 7:** Pre-Imputation QC steps for each genotype group for NCDS

|  |  | |  | | |  |  |  |
| --- | --- | --- | --- | --- | --- | --- | --- | --- |
|  | **WTCCC2** | | **T1DGC** | | | **WTCCC1** | |  |
|  | N of Variants | N of individuals | | N of Variants | N of individuals | N of Variants | N of individuals |  |
| Start | 1,157,986 | 2,922 | 561,303 | | 2,592 | 490,032 | 1,502 |  |
| Duplicates | 1,157,986 | 2,916 | 561,303 | | 2,592 | 490,032 | 1,496 |  |
| MAF | 964,060 | 2,916 | 536,881 | | 2,592 | 420,099 | 1,496 |  |
| Missing data | 941,084 | 2,774 | 531,152 | | 2,545 | 373,076 | 1,476 |  |
| HWE | 934,674 | 2,774 | 529,691 | | 2,545 | 371,722 | 1,476 |  |
| Pruning | 121,437 | 2,774 | 98,911 | | 2,545 | 80,684 | 1,476 |  |
| Adding Phenotype | 121,437 | 2,774 | 98,911 | | 2,545 | 80,684 | 1,476 |  |
| Check gender | 121,437 | 2,771 | 98,911 | | 2,540 | -------- | -------- |  |
| IBD check | 121,437 | 2,769 | 98,911 | | 2,540 | 80,684 | 1,473 |  |
| Pop strat | 121,437 | 2,769 | 98,911 | | 2,514 | 80,684 | 1,456 |  |
| Heterozygosity | 121,437 | 2,631 | 98,911 | | 2,485 | 80,684 | 1,432 |  |
|  |  |  |  | |  |  |  |  |
| *Preparation for Imputation* | | | | | | | |  |
| Start | 934,674 | 2,631 | 529,691 | | 2,485 | 371,722 | 1,432 |  |
| Liftover (un-pruned) | 908,075 | 2,631 | 516,922 | | 2,485 | 371,657 | 1,432 |  |
| SNPFlip (un-pruned) |  |  |  | |  |  |  |  |
| - SNPs flipped | 446,061 | 2,631 | 258,708 | | 2,485 | 58 | 1,432 |  |
| - SNP ambiguous | 16,358 | 2,631 | 0 | | 2,485 | 60,678 | 1,432 |  |
| Finish | 891,717 | 2,631 | 516,922 | | 2,485 | 310,979 | 1,432 |  |
| VCF files |  |  |  | |  |  |  |  |

Note: Duplicates = Removal of duplicated individuals, MAF = Discarding SNPs with MAF <1%, Missing data = variants or samples with missing data or data of low quality were removed (90-99% threshold in 1% intervals), HWE = Removal of SNPs with Hardy-Weinberg Equilibrium p<1x10-5, Pruning = Pruning for LD (r2<0.2) & excl. high-LD/ non-autosomal regions, Gender Check = Check mismatching phenotype & genetic sex, IBD check = Identical-by-descent checks (pi-hat<0.1875), Pop Strat = Population stratification & ancestry groupings were run using EIGENSTRAT (5) and PERL (6), Ancestry outliers were plotted in R v3.6.1 (7) using the 1,000 Genome reference panel (8) Heterozygosity = Check for unusual patterns of genome-wide Heterozygosity, LiftOver = Lift-Over from genome-built 36 to Build 37 for WTCCC2 & T1DGC and Lift-Over from genome-built 35 to Build 37 for WTCCC1 using liftOverPlink (9), SNPFlip = SNPflip (flipping of reverse strand SNPs) v0.0.6 (10), VCF files = creation of individual chromosome VCF files for imputation

**Supplementary Fig 1:** WTCCC2 ancestry grouping (NCDS)

**Supplementary Fig 2:** T1DGC ancestry grouping (NCDS)

# **Supplementary Fig 3:** WTCCC1 ancestry grouping (NCDS)

Note: All ancestry groupings were plotted using R (7), ASW = Americans of African Ancestry in SW, CEU = Utah Residents (CEPH) with Northern and Western European Ancestry, CHB = Han Chinese in Beijing, China, CHS = Han Chinese South, CLM = Colombians from Medellin, Colombia, FIN = Finnish from Finland, GBR = British England and Scotland, IBS = Iberian Population in Spain, JPT = Japanese in Tokyo, Japan, LWK = Luhya in Webuye, Kenya, MXL = Mexican Ancestry from Los Angeles, PUR = Puerto Ricans from Puerto Rico, TSI = Toscani in Italy, YRI = Yoruba in Ibadan, Nigeria, CONTROLNCDS refers to WTCCC2, T1DGC & WTCCC1 sample; All line graphs were plotted in Microsoft excel displaying the top 100 principal components

# **Supplementary Table 8:** Imputation for NCDS

|  | **WTCCC2** | **T1DGC** | **WTCC1** |
| --- | --- | --- | --- |
| Start | 891,717 | 516,922 | 310,979 |
| Sample size | 2,631 | 2,485 | 1,432 |
| Chunks | 154 | 153 | 153 |
| Reference Overlap | 99.62% | 99.72% | 99.72% |
| Matches | 619,669 | 357,201 | 211,735 |
| Allele switches | 265,428 | 156,682 | 97,479 |
| Invalid alleles | 4 | 0 | 0 |
| Duplicated sites | 139 | 0 | 0 |
| Allele mismatches | 3,070 | 1,617 | 909 |
|  |  |  |  |
| *Imputation summary* |  |  |  |
| Sites excluded | 3,213 | 1,617 | 909 |
| Sites remaining | 885,097 | 513,883 | 309,214 |
| Typed only sites | 3,407 | 1,422 | 856 |
| Chunks excluded | 1 | 0 | 1 |
| Chunks remaining | 153 | 153 | 152 |

Note: Imputation has been performed using the Michigan imputation server (11), Genotype Imputation using Minimac4 v1.2.4, Reference panel: 1000G Phase 3 v5, Array Build: GRCH37/hg19, Phasing: Eagle v2.4 (12), Population: EUR, Data type: Unphased

# **Supplementary Table 9:** Post-Imputation QC for NCDS

|  |  |  |
| --- | --- | --- |

|  | **WTCCC2** | | | **T1DGC** | | | **WTCCC1** | |
| --- | --- | --- | --- | --- | --- | --- | --- | --- |
|  | N of Variants | | N of individuals | N of Variants | N of individuals | | N of variants | N of individuals |
| *Imputation quality & Posterior genotype probability confidence filter* | | | | | | | | |
| > Chromosome 1 | 869,554 | | 2,631 | 738,102 | | 2,485 | 503,497 | 1,432 |
| > Chromosome 2 | 961,075 | | 2,631 | 863,333 | | 2,485 | 598,167 | 1,432 |
| > Chromosome 3 | 825,382 | | 2,631 | 739,629 | | 2,485 | 506,099 | 1,432 |
| > Chromosome 4 | 826,906 | | 2,631 | 733,811 | | 2,485 | 507,345 | 1,432 |
| > Chromosome 5 | 747,037 | | 2,631 | 669,215 | | 2,485 | 470,437 | 1,432 |
| > Chromosome 6 | 795,618 | | 2,631 | 728,751 | | 2,485 | 527,897 | 1,432 |
| > Chromosome 7 | 659,356 | | 2,631 | 573,192 | | 2,485 | 378,350 | 1,432 |
| > Chromosome 8 | 633,722 | | 2,631 | 577,899 | | 2,485 | 399,525 | 1,432 |
| > Chromosome 9 | 493,179 | | 2,631 | 440,602 | | 2,485 | 286,753 | 1,432 |
| > Chromosome 10 | 585,237 | | 2,631 | 519,747 | | 2,485 | 376,148 | 1,432 |
| > Chromosome 11 | 567,225 | | 2,631 | 494,897 | | 2,485 | 361,085 | 1,432 |
| > Chromosome 12 | 550,836 | | 2,631 | 478,980 | | 2,485 | 332,166 | 1,432 |
| > Chromosome 13 | 420,325 | | 2,631 | 383,005 | | 2,485 | 267,388 | 1,432 |
| > Chromosome 14 | 372,767 | | 2,631 | 329,884 | | 2,485 | 215,359 | 1,432 |
| > Chromosome 15 | 314,484 | | 2,631 | 272,987 | | 2,485 | 168,928 | 1,432 |
| > Chromosome 16 | 326,080 | | 2,631 | 274,563 | | 2,485 | 160,298 | 1,432 |
| > Chromosome 17 | 280,681 | | 2,631 | 228,988 | | 2,485 | 114,821 | 1,432 |
| > Chromosome 18 | 317,537 | | 2,631 | 285,365 | | 2,485 | 184,805 | 1,432 |
| > Chromosome 19 | 224,446 | | 2,631 | 173,234 | | 2,485 | 65,439 | 1,432 |
| > Chromosome 20 | 241,152 | | 2,631 | 213,135 | | 2,485 | 135,946 | 1,432 |
| > Chromosome 21 | 146,312 | | 2,631 | 129,702 | | 2,485 | 75,839 | 1,432 |
| > Chromosome 22 | 146,771 | | 2,631 | 123,989 | | 2,485 | 60,822 | 1,432 |
| *Exclude failed SNPs* |  | |  |  | |  |  |  |
| > Chromosome 1 | 869,553 | | 2,631 | 738,102 | | 2,485 | 503,496 | 1,432 |
| > Chromosome 2 | 961,073 | | 2,631 | 863,331 | | 2,485 | 598,167 | 1,432 |
| > Chromosome 3 | 825,381 | | 2,631 | 739,627 | | 2,485 | 506,099 | 1,432 |
| > Chromosome 4 | 826,904 | | 2,631 | 733,810 | | 2,485 | 507,353 | 1,432 |
| > Chromosome 5 | 747,0,37 | | 2,631 | 669,215 | | 2,485 | 470,437 | 1,432 |
| > Chromosome 6 | 795,617 | | 2,631 | 728,749 | | 2,485 | 527,896 | 1,432 |
| > Chromosome 7 | 659,356 | | 2,631 | 573,192 | | 2,485 | 378,350 | 1,432 |
| > Chromosome 8 | 633,721 | | 2,631 | 577,895 | | 2,485 | 399,524 | 1,432 |
| > Chromosome 9 | 493,178 | | 2,631 | 440,602 | | 2,485 | 286,753 | 1,432 |
| > Chromosome 10 | 585,235 | | 2,631 | 519,745 | | 2,485 | 376,147 | 1,432 |
| > Chromosome 11 | 567,224 | | 2,631 | 494,897 | | 2,485 | 361,085 | 1,432 |
| > Chromosome 12 | 550,834 | | 2,631 | 478,978 | | 2,485 | 332,165 | 1,432 |
| > Chromosome 13 | 420,324 | | 2,631 | 383,003 | | 2,485 | 267,387 | 1,432 |
| > Chromosome 14 | 372,767 | | 2,631 | 329,884 | | 2,485 | 215,359 | 1,432 |
| > Chromosome 15 | 314,484 | | 2,631 | 272,987 | | 2,485 | 168,928 | 1,432 |
| > Chromosome 16 | 326,080 | | 2,631 | 274,563 | | 2,485 | 160,298 | 1,432 |
| > Chromosome 17 | 280,681 | | 2,631 | 228,988 | | 2,485 | 114,821 | 1,432 |
| > Chromosome 18 | 317,537 | | 2,631 | 285,365 | | 2,485 | 184,805 | 1,432 |
| > Chromosome 19 | 224,445 | | 2,631 | 173,234 | | 2,485 | 65,439 | 1,432 |
| > Chromosome 20 | 241,152 | | 2,631 | 213,135 | | 2,485 | 135,946 | 1,432 |
| > Chromosome 21 | 146,312 | | 2,631 | 129,702 | | 2,485 | 75,839 | 1,432 |
| > Chromosome 22 | 146,771 | | 2,631 | 123,989 | | 2,485 | 60,822 | 1,432 |
| *Exclude duplicate SNPs* | | | | | | | | |
| > Chromosome 1 | 869,539 | | 2,631 | 738,092 | | 2,485 | 503,488 | 1,432 |
| > Chromosome 2 | 961,053 | | 2,631 | 863,311 | | 2,485 | 598,155 | 1,432 |
| > Chromosome 3 | 825,369 | | 2,631 | 739,617 | | 2,485 | 506,093 | 1,432 |
| > Chromosome 4 | 826,890 | | 2,631 | 733,796 | | 2,485 | 507,345 | 1,432 |
| > Chromosome 5 | 747,023 | | 2,631 | 669,215 | | 2,485 | 470,435 | 1,432 |
| > Chromosome 6 | 795,589 | | 2,631 | 728,723 | | 2,485 | 527,890 | 1,432 |
| > Chromosome 7 | 659,334 | | 2,631 | 573,174 | | 2,485 | 378,342 | 1,432 |
| > Chromosome 8 | 633,715 | | 2,631 | 577,889 | | 2,485 | 399,520 | 1,432 |
| > Chromosome 9 | 493,162 | | 2,631 | 440,590 | | 2,485 | 286,743 | 1,432 |
| > Chromosome 10 | 585,219 | | 2,631 | 519,733 | | 2,485 | 376,139 | 1,432 |
| > Chromosome 11 | 567,208 | | 2,631 | 494,887 | | 2,485 | 361,075 | 1,432 |
| > Chromosome 12 | 550,818 | | 2,631 | 478,966 | | 2,485 | 332,155 | 1,432 |
| > Chromosome 13 | 420,320 | | 2,631 | 382,997 | | 2,485 | 267,387 | 1,432 |
| > Chromosome 14 | 372,761 | | 2,631 | 329,880 | | 2,485 | 215,357 | 1,432 |
| > Chromosome 15 | 314,480 | | 2,631 | 272,985 | | 2,485 | 168,926 | 1,432 |
| > Chromosome 16 | 326,074 | | 2,631 | 274,557 | | 2,485 | 160,298 | 1,432 |
| > Chromosome 17 | 280,661 | | 2,631 | 228,974 | | 2,485 | 114,817 | 1,432 |
| > Chromosome 18 | 317,531 | | 2,631 | 285,361 | | 2,485 | 184,805 | 1,432 |
| > Chromosome 19 | 224,435 | | 2,631 | 173,232 | | 2,485 | 65,439 | 1,432 |
| > Chromosome 20 | 241,148 | | 2,631 | 213,135 | | 2,485 | 135,946 | 1,432 |
| > Chromosome 21 | 146,310 | | 2,631 | 129,700 | | 2,485 | 75,837 | 1,432 |
| > Chromosome 22 | 146,767 | | 2,631 | 123,987 | | 2,485 | 60,822 | 1,432 |
|  |  | |  |  | |  |  |  |
| *Merging of chromosomes & QC* | |  |  |  | |  |  |  |
| Merging chromosomes | 11,305,406 | | 2,631 | 9,972,791 | | 2,485 | 6,697,012 | 1,432 |
| Update varID with rsID | 11,062,080 | | 2,631 | 9,757,707 | | 2,485 | 6,556,279 | 1,432 |
| MAF | 6,329018 | | 2,631 | 6,067,828 | | 2,485 | 4,653,890 | 1,432 |
| Removing missing SNPs | 6,329,018 | | 2,631 | 6,067,828 | | 2,485 | 4,653,890 | 1,432 |
| Check completeness | 6,329,018 | | 2,631 | 6,067,828 | | 2,485 | 4,653,890 | 1,432 |
| Update sex | 6,329,018 | | 2,631 | 6,067,828 | | 2,485 | ----------- | ----------- |

Note: Imputation quality & Posterior genotype probability confidence filter = VCF output files filtered by R2 >.8 and genotype probability threshold of GP >.8 using bcftools (13), Post-imputation QC performed in PLINK: Merging chromosomes 2-22 with chromosome 1 data, Update varID with rsID = Update variant ID with the rsID using 1000genomes v3, MAF = Discarding SNPs with MAF <5%, Updating SNP = Update SNP names using 1000 genomes file, Removing missing SNPs = Remove missing SNPs, incl. those set as missing (>0.01), Check completeness = Removing individuals < 99% complete data

#

# **Supplementary Table 10:** Combining of WTCCC2 , T1DGC and WTCCC1 genetic information into a single dataset - NCDS

|  | |  | | |  | | |  | |  |
| --- | --- | --- | --- | --- | --- | --- | --- | --- | --- | --- |
|  | **WTCCC2** | | | **T1DGC** | | | **WTCCC1** | | | |
|  | N of Variants | | N of individuals | N of Variants | | N of individuals | N of variants | | N of individuals | |
| Start | 6,328,621 | | 2,631 | 6,067,828 | | 2,485 | 4,653,890 | | 1,432 | |
| Exclude tri-allelic | 1,041 | | | | | | | | | |
| Merging datasets | **6,398,736** variants and **5,288** individuals | | | | | | | | | |

Note: Exclude tri-allelic = Remove tri-allelic variants from dataset. Merging = merging of datasets using PLINK (14).

# **Supplementary Table 11A:** Creation of PRS - NCDS

|  | SCZ | MDD |
| --- | --- | --- |
| **Target file** |  | |
| Start SNPs target file | 6,398,736 | |
| Ambiguous SNPs | 888,420 | |
| Total SNPs included | 5,510,316 | |
| **Base file** |  |  |
| Start SNPs base file | 7,589,911 | 13,553,074 |
| Ambiguous SNPs | 5 | 62 |
| SNPs not found | 3,152,029 | 8,546,327 |
| Mismatched SNPs | 13 | 265,140 |
| Info score | 380,492 | 381,258 |
| Total SNPs included | 4,057,385 | 4,361,112 |
| Variants after clumping | 89,331 | 101,553 |

Note: PRS were created for each individual using PRSice 2.2.1 (15), Target file = Combined WTCCC2 and TDGC1 dataset, Base file for SCZ = Schizophrenia Consortium (16) and base file for MDD = Wray et al (17) were downloaded from the Psychiatric Genetics Consortium, Ambiguous SNPs were excluded, Mismatched SNPs were excluded, Info score = SNPs with info score of less than 0.9 were excluded, LD threshold for clumping (r2 < 0.1), The following PRS thresholds were used: .01, .1, .2, .3, .4, .5, 1

# **Supplementary Table 11B:** PRS Base file summary statistics - NCDS

|  | SCZ | MDD |
| --- | --- | --- |
| Number of studies | 73 | 33 |
| Number of cases | 49,881 | 59,369 |
| Number of controls | 69,697 | 110,318 |
| Reference panel | 1,000 Genomes | 1,000 Genomes |
| Summary statistics created | 2021 | 2021 |

Note: Base file for SCZ = Schizophrenia Consortium (16) without cohorts from the UK (but not Ireland) with 7,589,912 autosomal SNPs, Base file from MDD = Wray et al (17) without 23andMe and GenPod

#

# **Supplementary Table 12:** Principal Components – Combined NCDS

|  | Combined NCDS |
| --- | --- |
| Eigenvectors selected | 2.266  1.822  1.756  1.732  1.695 |

Note: Top 100 PCAs were calculated from LD-pruned full cohort dataset using EIGENSTRAT, Eigenvectors selected = eigenvectors used for covariates which explain the majority of the variance

#

# **Supplementary Fig 4:** Principal Components – Combined NCDS

Note: All Principal Components were plotted using Microsoft Excel

# **Supplementary Table 13:** Merging of genotype, phenotype, eigenvector and PRS information (separate merges for SCZ and MDD) – NCDS

| 1. Raw PRS merged with Principal Components into single dataset |
| --- |
| 1. PRS/Principal Components merged with phenotype data into final single dataset |
| 1. Regression of PRS for each threshold using Principal components, sex and year as covariates |

Note: Datasets were merged using STATA v12.1 (18).

# **Supplementary Table 14:** Power Calculation for USoc

| Power calculation model used | F-test (Fixed linear regression) |  |
| --- | --- | --- |
| Power | 0.8 |  |
| Effect size | 0.005 |  |
| Error rate | 0.002778 (=0.05/18 environments tested) |  |
| Number of tested predictors | 1 |  |
| **Minimum sample size required** | **2,943** |  |

Note: Minimum sampe size calculated using G*Power 3.1.9.6 (1). Effect sizes are based on similar rGE studies (2)

# **Supplementary Table 15:** for USoc dataset process flow

Pre-imputation genetic QC (*table 16*), including creation of PC (*table 17, Fig 7 & 8*)

Imputation of genetic dataset (*table 18*)

Post-imputation genetic QC (*table 19*)

Creation of individual PRS scores (*table 20A & B*)

Merging of genetic, phenotype eigenvector & PRS information (*table 21)*

Removal of genetically unrelated individuals from same household *(table 21A & B)*

Note: Understanding Society – 9,921 individuals genotyped using Illumina Infinium HumanCoreExome BeadChip Kit by the Wellcome Trust Sanger Institute (19), PRS = Polygenic Risk Scores

# **Supplementary Table 16:** Pre-Imputation QC steps for USoc

|  |  |  | |  | |  |
| --- | --- | --- | --- | --- | --- | --- |
|  | Number of Variants | | Number of individuals | |  | |
| Start | 248,606 | | 9,921 | |  |  |
| Duplicates | 248,606 | | 9,921 | |  |  |
| MAF | 248,606 | | 9,908 | |  |  |
| Missing data | 245,488 | | 9,908 | |  |  |
| HWE | 245,488 | | 9,908 | |  |  |
| Pruning | 58,856 | | 9,908 | |  |  |
| Adding Phenotype | 58,856 | | 9,908 | |  |  |
| Check gender | 58,856 | | 9,880 | |  |  |
| IBD check | 58,856 | | 9,133 | |  |  |
| Pop strat | 58,856 | | 9,076 | |  |  |
| Heterozygosity | 58,856 | | 9,039 | |  |  |
|  | | | | | | |
| *Preparation for Imputation* | | | | | | |
| Start | 245,488 | | 9,039 | |  |  |
| SNPFlip |  | |  | |  |  |
| - SNPs flipped | 315 | | 9,039 | |  |  |
| - SNP ambiguous | 8,375 | | 9,039 | |  |  |
| Finish | 236,798 | | 9,039 | |  |  |
| VCF files |  | |  | |  |  |

Note: Understanding Society sample includes genome-wide significant SNPs only, Duplicates = Removal of duplicated individuals, MAF = Discarding SNPs with MAF <1%, Missing data = variants or samples with missing data or data of low quality were removed (90-99% threshold in 1% intervals), HWE = Removal of SNPs with Hardy-Weinberg Equilibrium p<1x10-5, Pruning = Pruning for LD (r2<0.2) & excl. high-LD/ non-autosomal regions, Gender Check = Check mismatching phenotype & genetic sex, IBD check = Identical-by-descent checks (pi-hat<0.1875), Pop Strat = Population stratification & ancestry groupings were run using EIGENSTRAT (5) and PERL (6), Ancestry outliers were plotted in R v3.6.1 (7) using the 1,000 Genome reference panel (8) Heterozygosity = Check for unusual patterns of genome-wide Heterozygosity (> or < 3SD from mean), SNPFlip = SNPflip (flipping of reverse strand SNPs) v0.0.6 (10), VCF files = creation of individual chromosome VCF files for imputation

# **Supplementary Table 17:** Principal Components Selection for USoc

|  |  |
| --- | --- |
| Eigenvectors selected | 3.228  2.247  2.184  2.181 |
|  |  |

Note: Top 100 PCAs run using EIGENSTRAT, Eigenvectors selected = eigenvectors used for covariates which explain the majority of the variance

# **Supplementary Fig 7:** Principal Components Plotting for USoc

Note: All Principal Components were plotted using Microsoft Excel

# **Supplementary Fig 8:** Ancestry grouping for USoc

Note: All ancestry groupings were plotted using R (7), ASW = Americans of African Ancestry in SW, CEU = Utah Residents (CEPH) with Northern and Western European Ancestry, CHB = Han Chinese in Beijing, China, CLM = Colombians from Medellin, Colombia, FIN = Finnish from Finland, GBR = British England and Scotland, IBS = Iberian Population in Spain, JPT = Japanese in Tokyo, Japan, LWK = Luhya in Webuye, Kenya, MXL = Mexican Ancestry from Los Angeles, PUR = Puerto Ricans from Puerto Rico, TSI = Toscani in Italy, YRI = Yoruba in Ibadan, Nigeria, CONTROLUSC refers to Understanding Society sample

# **Supplementary Table 18:** Imputation for USoc

|  |  |
| --- | --- |
| Start | 236,798 |
| Sample size | 9,039 |
| Chunks | 153 |
| Reference Overlap | 99.48 % |
| Matches | 157,329 |
| Allele switches | 77,453 |
| Invalid alleles | 0 |
| Duplicated sites | 91 |
| Allele mismatches | 684 |
|  |  |
| *Imputation summary* |  |
| Sites excluded | 775 |
| Sites remaining | 234,782 |
| Typed only sites | 1,241 |
| Chunks excluded | 1 |
| Chunks remaining | 152 |

Note: Imputation has been performed using the Michigan imputation server (11), Genotype Imputation using Minimac4 v1.2.4, Reference panel: 1000G Phase 3 v5, Array Build: GRCH37/hg19, Phasing: Eagle v2.4 (12), Population: EUR, Data type: Unphased

# **Supplementary Table 19:** Post-Imputation QC for USoc

|  |  | |  |  |  | |
| --- | --- | --- | --- | --- | --- | --- |
|  | Number of Variants | | Number of individuals | | |  |
| *Imputation quality & Posterior genotype probability confidence filter* | | | | | |  |
| > Chromosome 1 | 553,057 | | 9,039 | | |  |
| > Chromosome 2 | 654,045 | | 9,039 | | |  |
| > Chromosome 3 | 566,942 | | 9,039 | | |  |
| > Chromosome 4 | 569,940 | | 9,039 | | |  |
| > Chromosome 5 | 503,688 | | 9,039 | | |  |
| > Chromosome 6 | 578,953 | | 9,039 | | |  |
| > Chromosome 7 | 431,976 | | 9,039 | | |  |
| > Chromosome 8 | 436,913 | | 9,039 | | |  |
| > Chromosome 9 | 315,396 | | 9,039 | | |  |
| > Chromosome 10 | 386,027 | | 9,039 | | |  |
| > Chromosome 11 | 380,947 | | 9,039 | | |  |
| > Chromosome 12 | 357,304 | | 9,039 | | |  |
| > Chromosome 13 | 291,946 | | 9,039 | | |  |
| > Chromosome 14 | 240,990 | | 9,039 | | |  |
| > Chromosome 15 | 183,134 | | 9,039 | | |  |
| > Chromosome 16 | 185,862 | | 9,039 | | |  |
| > Chromosome 17 | 150,619 | | 9,039 | | |  |
| > Chromosome 18 | 200,343 | | 9,039 | | |  |
| > Chromosome 19 | 116,075 | | 9,039 | | |  |
| > Chromosome 20 | 147,322 | | 9,039 | | |  |
| > Chromosome 21 | 87,441 | | 9,039 | | |  |
| > Chromosome 22 | 76,155 | | 9,039 | | |  |
| *Exclude failed SNPs* |  | |  | | |  |
| > Chromosome 1 | 553,057 | | 9,039 | | |  |
| > Chromosome 2 | 654,025 | | 9,039 | | |  |
| > Chromosome 3 | 566,940 | | 9,039 | | |  |
| > Chromosome 4 | 569,938 | | 9,039 | | |  |
| > Chromosome 5 | 503,688 | | 9,039 | | |  |
| > Chromosome 6 | 578,953 | | 9,039 | | |  |
| > Chromosome 7 | 431,974 | | 9,039 | | |  |
| > Chromosome 8 | 436,911 | | 9,039 | | |  |
| > Chromosome 9 | 315,396 | | 9,039 | | |  |
| > Chromosome 10 | 386,025 | | 9,039 | | |  |
| > Chromosome 11 | 380,947 | | 9,039 | | |  |
| > Chromosome 12 | 357,302 | | 9,039 | | |  |
| > Chromosome 13 | 291,946 | | 9,039 | | |  |
| > Chromosome 14 | 240,990 | | 9,039 | | |  |
| > Chromosome 15 | 183,134 | | 9,039 | | |  |
| > Chromosome 16 | 185,862 | | 9,039 | | |  |
| > Chromosome 17 | 150,619 | | 9,039 | | |  |
| > Chromosome 18 | 200,343 | | 9,039 | | |  |
| > Chromosome 19 | 116,075 | | 9,039 | | |  |
| > Chromosome 20 | 147,322 | | 9,039 | | |  |
| > Chromosome 21 | 87,441 | | 9,039 | | |  |
| > Chromosome 22 | 76,155 | | 9,039 | | |  |
|  |  | |  | | |  |
| *Exclude duplicate SNPs* |  | |  | | |  |
| > Chromosome 1 | 553,051 | | 9,039 | | |  |
| > Chromosome 2 | 654,025 | | 9,039 | | |  |
| > Chromosome 3 | 566,932 | | 9,039 | | |  |
| > Chromosome 4 | 569,930 | | 9,039 | | |  |
| > Chromosome 5 | 503,680 | | 9,039 | | |  |
| > Chromosome 6 | 578,939 | | 9,039 | | |  |
| > Chromosome 7 | 431,964 | | 9,039 | | |  |
| > Chromosome 8 | 436,909 | | 9,039 | | |  |
| > Chromosome 9 | 315,386 | | 9,039 | | |  |
| > Chromosome 10 | 386,013 | | 9,039 | | |  |
| > Chromosome 11 | 380,947 | | 9,039 | | |  |
| > Chromosome 12 | 357,294 | | 9,039 | | |  |
| > Chromosome 13 | 291,944 | | 9,039 | | |  |
| > Chromosome 14 | 240,988 | | 9,039 | | |  |
| > Chromosome 15 | 183,132 | | 9,039 | | |  |
| > Chromosome 16 | 185,860 | | 9,039 | | |  |
| > Chromosome 17 | 150,609 | | 9,039 | | |  |
| > Chromosome 18 | 200,339 | | 9,039 | | |  |
| > Chromosome 19 | 116,075 | | 9,039 | | |  |
| > Chromosome 20 | 147,322 | | 9,039 | | |  |
| > Chromosome 21 | 87,439 | | 9,039 | | |  |
| > Chromosome 22 | 76,155 | | 9,039 | | |  |
|  |  | |  | | |  |
| *Merging of chromosomes & QC* | |  |  | | |  |
| Merging chromosomes | 7,414,923 | | 9,039 | | |  |
| Update varID with rsID | 7,257,111 | | 9,039 | | |  |
| MAF | 5,218,682 | | 9,039 | | |  |
| Removing missing SNPs | 5,218,682 | | 9,039 | | |  |
| Check completeness | 5,218,682 | | 9,039 | | |  |
| Remove duplicate SNPs | 5,218,682 | | 9,039 | | |  |
| Update sex | 5,218,682 | | 9,039 | | |  |

Note: Imputation quality & Posterior genotype probability confidence filter = VCF output files filtered by R2 >.8 and genotype probability threshold of GP >.8 using bcftools (13), Post-imputation QC performed in PLINK: Merging chromosomes 2-22 with chromosome 1 data, Update varID with rsID = Update variant ID with the rsID using 1000genomes v3, MAF = Discarding SNPs with MAF <5%, Updating SNP = Update SNP names using 1000 genomes file, Removing missing SNPs = Remove missing SNPs, incl. those set as missing (>0.01), Check completeness = Removing individuals < 99% complete data

# **Supplementary Table 20A:** Creation of PRS for USoc

|  | SCZ | MDD |
| --- | --- | --- |
| **Target file** |  | |
| Start SNPs target file | 6,427,282 | |
| Ambiguous SNPs | 1 | |
| Total SNPs found & included | 4,487,120 | |
| **Base file** |  |  |
| Start SNPs base file | 9,444,230 | 13,554,550 |
| Ambiguous SNPs | 68 | 49 |
| SNPs not found in target file | 5,634,770 | 9,457,169 |
| Mismatched SNPs | 1,675 | 191,392 |
| Info score | 0 | 153,937 |
| Total SNPs included | 3,807,785 | 3,752,443 |
| Variants after clumping | 79,234 | 75,527 |

Note: PRS were created for each individual using PRSice 2.2.1 (15), Target file = Understanding Society, Base file for SCZ = Schizophrenia Consortium (16) and base file for MDD = Wray et al (17) were downloaded from the Psychiatric Genetics Consortium, Ambiguous SNPs were excluded, Mismatched SNPs were excluded, Info score = SNPs with info score of less than 0.9 were excluded, LD threshold for clumping (r2 < 0.1), The following PRS thresholds were used: .01, .1, .2, .3, .4, .5, 1

# **Supplementary Table 20B:** PRS Base file summary statistics for USoc

|  | SCZ | MDD |
| --- | --- | --- |
| **Test sample** |  |  |
| Number of samples | 49 | 29 |
| Sample cases | 34,241 | 16,823 |
| Sample controls | 45,604 | 25,632 |
| Family association studies samples | 1,235 | - |
| **Replication sample** |  |  |
| Number of samples | 2 | 6 |
| Sample cases | 1,513 | 118,635 |
| Sample controls | 66,236 | 319,269 |
| Reference panel | 1,000 Genomes | 1,000 Genomes |
| Publication year | 2014 | 2018 |
| Number of loci identified | 108 | 44 |

Note: Base file for SCZ = Schizophrenia Consortium (16), Base file for MDD = Wray et al (17)

**Supplementary Table 21:** Merging of genotype, phenotype, eigenvector and PRS information (separate merges for schizophrenia and major depressive disorder) for USoc

| 1. Merge Principal Components with phenotype dataset |
| --- |
| 1. Random selection of one individual from each household for which Principal Components are available |
| 1. Merge raw PRS with random individual single dataset for SCZ and MDD |
| 1. Regression of PRS for each threshold using Principal components, age, data wave year and sex as covariates |

Note: Datasets were merged using STATA v12.1 (18).

# **Supplementary Table 22A:** Random selection of one individual from each household for USoc

|  | N removed | N remaining |
| --- | --- | --- |
| Start | - | 9,921 |
| Keep genotyped individuals with Principal components scores only | 845 | 9,076 |
| Wave 1 | 1,124 | 7,952 |
| Wave 2 | 512 | 7,440 |
| Wave 3 | 21 | 7,419 |
| Wave 4 | 1 | 7,418 |
| Wave 5 | 0 | 7,418 |
| Wave 6 | 0 | 7,418 |
| Wave 7 | 2 | 7,416 |
| Wave 8 | 0 | 7,416 |
| Wave 9 | 0 | 7,416 |
| Keep genotyped individuals with PRS scores only | 31 | 7,384 |

Note: PCA were created for all genotyped individuals which passed QC before outliers were removed and merged with the total phenotype dataset. A single random sample dataset of genetically unrelated individuals from different households was created. All dataset mergers were performed using STATA v12.1 (18).

# **Supplementary Table 22B:** Age of individuals for USoc

As this study is focusing on adults over the age of 16, any responses from individuals who were less than 16 years of age and born in 1995 (18 individuals) and 1994 (43 individuals) were excluded from wave 1 (data collection between 2009-2010) (20). Responses from individuals who were born in 1995 (16 individuals) were excluded from wave 2 (data collection between 2010-2011) (20).

# **References:**

1. Faul F, Erdfelder E, Buchner A, Lang AG. Statistical power analyses using G*Power 3.1: tests for correlation and regression analyses. Behav Res Methods. 2009;41(4):1149-60.

2. Krapohl E, Hannigan LJ, Pingault JB, Patel H, Kadeva N, Curtis C, et al. Widespread covariation of early environmental exposures and trait-associated polygenic variation. Proc Natl Acad Sci U S A. 2017;114(44):11727-32.

3. Barrett JC, Clayton DG, Concannon P, Akolkar B, Cooper JD, Erlich HA, et al. Genome-wide association study and meta-analysis find that over 40 loci affect risk of type 1 diabetes. Nat Genet. 2009;41(6):703-7.

4. Wellcome Trust Case Control Consortium. Genome-wide association study of 14,000 cases of seven common diseases and 3,000 shared controls. Nature. 2007;447(7145):661-78.

5. Price AL, Patterson NJ, Plenge RM, Weinblatt ME, Shadick NA, Reich D. Principal components analysis corrects for stratification in genome-wide association studies. Nat Genet. 2006;38(8):904-9.

6. Patterson N, Price AL, Reich D. Population structure and eigenanalysis. PLoS Genet. 2006;2(12):e190.

7. R Core Team. R: A Language and Environment for Statistical Computing. Vienna, Austria: R Foundation for Statistical Computing; 2018.

8. Auton A, Brooks LD, Durbin RM, Garrison EP, Kang HM, Korbel JO, et al. A global reference for human genetic variation. Nature. 2015;526(7571):68-74.

9. Ritchie S. liftOverPlink. GitHub; 2014.

10. Bakken Stovner E. snpflip. GitHub2017.

11. Das S, Forer L, Schönherr S, Sidore C, Locke AE, Kwong A, et al. Next-generation genotype imputation service and methods. Nat Genet. 2016;48(10):1284-7.

12. Loh PR, Danecek P, Palamara PF, Fuchsberger C, A Reshef Y, K Finucane H, et al. Reference-based phasing using the Haplotype Reference Consortium panel. Nat Genet. 2016;48(11):1443-8.

13. Danecek P, Bonfield JK, Liddle J, Marshall J, Ohan V, Pollard MO, et al. Twelve years of SAMtools and BCFtools. Gigascience. 2021;10(2).

14. Purcell S, Neale B, Todd-Brown K, Thomas L, Ferreira MA, Bender D, et al. PLINK: a tool set for whole-genome association and population-based linkage analyses. Am J Hum Genet. 2007;81(3):559-75.

15. Choi SW, O'Reilly PF. PRSice-2: Polygenic Risk Score software for biobank-scale data. Gigascience. 2019;8(7).

16. Schizophrenia Working Group of the Psychiatric Genomics Consortium. Biological insights from 108 schizophrenia-associated genetic loci. Nature. 2014;511(7510):421-7.

17. Wray NR, Ripke S, Mattheisen M, Trzaskowski M, Byrne EM, Abdellaoui A, et al. Genome-wide association analyses identify 44 risk variants and refine the genetic architecture of major depression. Nat Genet. 2018;50(5):668-81.

18. StataCorp. *Stata Statistical Software: Release 12*. College Station, TX: StataCorp LP; 2011.

19. Prins BP, Kuchenbaecker KB, Bao Y, Smart M, Zabaneh D, Fatemifar G, et al. Genome-wide analysis of health-related biomarkers in the UK Household Longitudinal Study reveals novel associations. Sci Rep. 2017;7(1):11008.

20. University of Essex, Institute for Social and Economic Research, NatCen Social Research, Kantar Public. Understanding Society: Waves 1-9, 2009-2018 and Harmonised BHPS: Waves 1-18, 1991-2009. [data collection]. 12th Edition ed: UK Data Service; 2019.
