# Supplementary material for: Polygenic risk scores for schizophrenia and major depression are associated with socio-economic indicators of adversity in two British community samples
Source: Transl Psychiatry. 2022 Nov 14;12:477. doi: 10.1038/s41398-022-02247-8 (PMC9663827; doi:10.1038/s41398-022-02247-8)
Supplement: Supplementary file 4 — Supplementary_4_SCZ_and_MDD_results [file 41398_2022_2247_MOESM4_ESM.docx]

**Supplementary Document – Full SCZ and MDD results**

Table of Contents

[Supplementary Table 23: All PRS results for NCDS 2](#_Toc86932228)

[Supplementary Table 24: All PRS results for USoc 6](#_Toc86932229)

[Supplementary Tables 25: NCDS environmental risk factor correlation matrix 12](#_Toc86932230)

[Supplementary Tables 26: USoc environmental risk factor correlation matrix 14](#_Toc86932231)

[References 23](#_Toc86932232)

# **Supplementary Table** **23**: All PRS results for NCDS

| **Environment** | **Threshold**  **z-scored** | **Beta** | **95%CI** | **P-Value** | **Bonferroni Correction** | | | **Sensitivity** | | | | **Interaction** | | |  |
| --- | --- | --- | --- | --- | --- | --- | --- | --- | --- | --- | --- | --- | --- | --- | --- |
|  |  |  |  |  | **0.05/30** | **statistically**  **significant?** | **Beta** | | **95%CI** | **P-Value** | **Wald**  **chi-**  **square** | | **p-value** |  |  |
| **SCZ** | | | | | | | | | | | | | | | |
| **SES** | 0.01 | 0 | -0.03-0.02 | 9.04E-01 | 1.67E-03 | FALSE |  | | | |  | | |  |  |
|  | 0.1 | 0.01 | -0.01-0.03 | 3.21E-01 | 1.67E-03 | FALSE |  |  |  |  |  |  |  |  |  |
|  | 0.2 | 0.02 | -0.01-0.04 | 1.90E-01 | 1.67E-03 | FALSE |  |  |  |  |  |  |  |  |  |
|  | 0.3 | 0.01 | -0.01-0.04 | 2.18E-01 | 1.67E-03 | FALSE |  |  |  |  |  |  |  |  |  |
|  | 0.4 | 0.02 | -0.01-0.04 | 1.69E-01 | 1.67E-03 | FALSE |  |  |  |  |  |  |  |  |  |
|  | 0.5 | 0.02 | -0.01-0.04 | 1.89E-01 | 1.67E-03 | FALSE |  |  |  |  |  |  |  |  |  |
|  | 1 | 0.02 | -0.01-0.04 | 1.77E-01 | 1.67E-03 | FALSE |  |  |  |  |  |  |  |  |  |
| **Number of Rooms** | 0.01 | 0 | -0.03-0.02 | 6.99E-01 | 1.67E-03 | FALSE |  | | | |  | | |  |  |
|  | 0.1 | 0 | -0.02-0.02 | 7.21E-01 | 1.67E-03 | FALSE |  |  |  |  |  |  |  |  |  |
|  | 0.2 | -0.01 | -0.03-0.01 | 4.11E-01 | 1.67E-03 | FALSE |  |  |  |  |  |  |  |  |  |
|  | 0.3 | -0.01 | -0.03-0.01 | 3.61E-01 | 1.67E-03 | FALSE |  |  |  |  |  |  |  |  |  |
|  | 0.4 | -0.01 | -0.03-0.01 | 3.75E-01 | 1.67E-03 | FALSE |  |  |  |  |  |  |  |  |  |
|  | 0.5 | -0.01 | -0.03-0.01 | 3.66E-01 | 1.67E-03 | FALSE |  |  |  |  |  |  |  |  |  |
|  | 1 | -0.01 | -0.03-0.01 | 3.92E-01 | 1.67E-03 | FALSE |  |  |  |  |  |  |  |  |  |
| **Marital status** | 0.01 | 0.06 | -0.01-0.13 | 8.36E-02 | 1.67E-03 | FALSE |  | | | |  | | |  |  |
|  | 0.1 | 0.03 | -0.03-0.10 | 3.29E-01 | 1.67E-03 | FALSE |  |  |  |  |  |  |  |  |  |
|  | 0.2 | 0.03 | -0.03-0.10 | 2.90E-01 | 1.67E-03 | FALSE |  |  |  |  |  |  |  |  |  |
|  | 0.3 | 0.03 | -0.03-0.10 | 3.34E-01 | 1.67E-03 | FALSE |  |  |  |  |  |  |  |  |  |
|  | 0.4 | 0.03 | -0.03-0.09 | 3.70E-01 | 1.67E-03 | FALSE |  |  |  |  |  |  |  |  |  |
|  | 0.5 | 0.02 | -0.04-0.09 | 4.70E-01 | 1.67E-03 | FALSE |  |  |  |  |  |  |  |  |  |
|  | 1 | 0.02 | -0.04-0.09 | 4.91E-01 | 1.67E-03 | FALSE |  |  |  |  |  |  |  |  |  |
| **Smoking** | 0.01 | 0.06 | -0.14-0.27 | 5.52E-01 | 1.67E-03 | FALSE |  | | | |  | | |  |  |
|  | 0.1 | 0.14 | -0.06-0.33 | 1.68E-01 | 1.67E-03 | FALSE |  |  |  |  |  |  |  |  |  |
|  | 0.2 | 0.15 | -0.05-0.34 | 1.34E-01 | 1.67E-03 | FALSE |  |  |  |  |  |  |  |  |  |
|  | 0.3 | 0.15 | -0.05-0.34 | 1.33E-01 | 1.67E-03 | FALSE |  |  |  |  |  |  |  |  |  |
|  | 0.4 | 0.14 | -0.06-0.33 | 1.71E-01 | 1.67E-03 | FALSE |  |  |  |  |  |  |  |  |  |
|  | 0.5 | 0.12 | -0.07-0.32 | 2.11E-01 | 1.67E-03 | FALSE |  |  |  |  |  |  |  |  |  |
|  | 1 | 0.12 | -0.07-0.32 | 2.07E-01 | 1.67E-03 | FALSE |  |  |  |  |  |  |  |  |  |
| **Employment** | 0.01 | 0.08 | -0.02-0.18 | 1.22E-01 | 1.67E-03 | FALSE |  | | | |  | | |  |  |
|  | 0.1 | 0.08 | -0.02-0.17 | 1.34E-01 | 1.67E-03 | FALSE |  |  |  |  |  |  |  |  |  |
|  | 0.2 | 0.07 | -0.02-0.17 | 1.36E-01 | 1.67E-03 | FALSE |  |  |  |  |  |  |  |  |  |
|  | 0.3 | 0.09 | -0.01-0.18 | 8.53E-02 | 1.67E-03 | FALSE |  |  |  |  |  |  |  |  |  |
|  | 0.4 | 0.08 | -0.02-0.18 | 9.87E-02 | 1.67E-03 | FALSE |  |  |  |  |  |  |  |  |  |
|  | 0.5 | 0.08 | -0.01-0.18 | 8.86E-02 | 1.67E-03 | FALSE |  |  |  |  |  |  |  |  |  |
|  | 1 | 0.07 | -0.02-0.17 | 1.33E-01 | 1.67E-03 | FALSE |  |  |  |  |  |  |  |  |  |
| **Tenure** | 0.01 | 0.01 | -0.11-0.13 | 8.39E-01 | 1.67E-03 | FALSE |  | | | |  | | |  |  |
|  | 0.1 | 0 | -0.11-0.11 | 9.65E-01 | 1.67E-03 | FALSE |  |  |  |  |  |  |  |  |  |
|  | 0.2 | 0.01 | -0.10-0.12 | 8.37E-01 | 1.67E-03 | FALSE |  |  |  |  |  |  |  |  |  |
|  | 0.3 | 0.01 | -0.10-0.12 | 8.11E-01 | 1.67E-03 | FALSE |  |  |  |  |  |  |  |  |  |
|  | 0.4 | 0.01 | -0.10-0.12 | 8.74E-01 | 1.67E-03 | FALSE |  |  |  |  |  |  |  |  |  |
|  | 0.5 | 0 | -0.11-0.11 | 9.62E-01 | 1.67E-03 | FALSE |  |  |  |  |  |  |  |  |  |
|  | 1 | 0.01 | -0.10-0.12 | 9.07E-01 | 1.67E-03 | FALSE |  |  |  |  |  |  |  |  |  |
| **MDD** | | | | | | | | | | | | | | | |
| **SES** | 0.01 | -0.02 | -0.04-0.00 | 6.29E-02 | 1.67E-03 | FALSE |  | | | |  | | |  |  |
|  | 0.1 | -0.02 | -0.05--0.00 | 2.86E-02 | 1.67E-03 | FALSE |  |  |  |  |  |  |  |  |  |
|  | 0.2 | -0.02 | -0.04-0.00 | 5.21E-02 | 1.67E-03 | FALSE |  |  |  |  |  |  |  |  |  |
|  | 0.3 | -0.02 | -0.04-0.00 | 9.59E-02 | 1.67E-03 | FALSE |  |  |  |  |  |  |  |  |  |
|  | 0.4 | -0.02 | -0.04-0.00 | 7.95E-02 | 1.67E-03 | FALSE |  |  |  |  |  |  |  |  |  |
|  | 0.5 | -0.02 | -0.04-0.00 | 8.95E-02 | 1.67E-03 | FALSE |  |  |  |  |  |  |  |  |  |
|  | 1 | -0.02 | -0.04-0.00 | 1.11E-01 | 1.67E-03 | FALSE |  |  |  |  |  |  |  |  |  |
| **Number of Rooms** | 0.01 | -0.03 | -0.04--0.01 | 8.93E-03* | 1.67E-03 | FALSE | -0.02 | | -0.05--0.00 | 5.00E-02 | 0.11 | | 7.38E-01 |  |  |
|  | 0.1 | -0.03 | -0.05--0.01 | **5.43E-04**** | 1.67E-03 | TRUE | -0.03 | | -0.05--0.01 | 9.02E-03 | 0.3 | | 5.82E-01 |  |  |
|  | 0.2 | -0.03 | -0.05--0.01 | 1.83E-03* | 1.67E-03 | FALSE | -0.02 | | -0.05--0.00 | 3.09E-02 | 0.19 | | 6.62E-01 |  |  |
|  | 0.3 | -0.03 | -0.05--0.01 | 1.72E-03* | 1.67E-03 | FALSE | -0.02 | | -0.05--0.00 | 3.05E-02 | 0.21 | | 6.48E-01 |  |  |
|  | 0.4 | -0.03 | -0.05--0.01 | **1.16E-03**** | 1.67E-03 | TRUE | -0.03 | | -0.05--0.00 | 2.28E-02 | 0.19 | | 6.62E-01 |  |  |
|  | 0.5 | -0.03 | -0.05--0.01 | **1.51E-03**** | 1.67E-03 | TRUE | -0.02 | | -0.05--0.00 | 3.22E-02 | 0.34 | | 5.58E-01 |  |  |
|  | 1 | -0.03 | -0.05--0.01 | 1.84E-03* | 1.67E-03 | FALSE | -0.02 | | -0.05--0.00 | 3.32E-02 | 0.32 | | 5.72E-01 |  |  |
| **Marital status** | 0.01 | -0.01 | -0.08-0.05 | 6.89E-01 | 1.67E-03 | FALSE |  | | | |  | | |  |  |
|  | 0.1 | 0 | -0.06-0.06 | 9.39E-01 | 1.67E-03 | FALSE |  |  |  |  |  |  |  |  |  |
|  | 0.2 | -0.02 | -0.08-0.04 | 5.20E-01 | 1.67E-03 | FALSE |  |  |  |  |  |  |  |  |  |
|  | 0.3 | -0.02 | -0.08-0.04 | 4.55E-01 | 1.67E-03 | FALSE |  |  |  |  |  |  |  |  |  |
|  | 0.4 | -0.02 | -0.09-0.04 | 4.29E-01 | 1.67E-03 | FALSE |  |  |  |  |  |  |  |  |  |
|  | 0.5 | -0.02 | -0.09-0.04 | 4.29E-01 | 1.67E-03 | FALSE |  |  |  |  |  |  |  |  |  |
|  | 1 | -0.02 | -0.08-0.04 | 4.77E-01 | 1.67E-03 | FALSE |  |  |  |  |  |  |  |  |  |
| **Smoking** | 0.01 | 0.21 | 0.02-0.40 | 3.06E-02* | 1.67E-03 | FALSE |  | | | |  | | |  |  |
|  | 0.1 | 0.22 | 0.03-0.41 | 2.06E-02* | 1.67E-03 | FALSE |  |  |  |  |  |  |  |  |  |
|  | 0.2 | 0.26 | 0.07-0.44 | 7.07E-03* | 1.67E-03 | FALSE |  |  |  |  |  |  |  |  |  |
|  | 0.3 | 0.26 | 0.07-0.44 | 6.57E-03* | 1.67E-03 | FALSE |  |  |  |  |  |  |  |  |  |
|  | 0.4 | 0.25 | 0.06-0.43 | 9.01E-03* | 1.67E-03 | FALSE |  |  |  |  |  |  |  |  |  |
|  | 0.5 | 0.24 | 0.05-0.42 | 1.20E-02* | 1.67E-03 | FALSE |  |  |  |  |  |  |  |  |  |
|  | 1 | 0.23 | 0.05-0.42 | 1.45E-02* | 1.67E-03 | FALSE |  |  |  |  |  |  |  |  |  |
| **Employment** | 0.01 | 0.08 | -0.01-0.18 | 8.92E-02 | 1.67E-03 | FALSE |  | | | |  | | |  |  |
|  | 0.1 | 0.05 | -0.05-0.14 | 3.18E-01 | 1.67E-03 | FALSE |  |  |  |  |  |  |  |  |  |
|  | 0.2 | 0.05 | -0.04-0.14 | 2.93E-01 | 1.67E-03 | FALSE |  |  |  |  |  |  |  |  |  |
|  | 0.3 | 0.05 | -0.04-0.15 | 2.70E-01 | 1.67E-03 | FALSE |  |  |  |  |  |  |  |  |  |
|  | 0.4 | 0.06 | -0.04-0.15 | 2.47E-01 | 1.67E-03 | FALSE |  |  |  |  |  |  |  |  |  |
|  | 0.5 | 0.05 | -0.04-0.15 | 2.78E-01 | 1.67E-03 | FALSE |  |  |  |  |  |  |  |  |  |
|  | 1 | 0.04 | -0.05-0.14 | 3.59E-01 | 1.67E-03 | FALSE |  |  |  |  |  |  |  |  |  |
| **Tenure** | 0.01 | 0.1 | -0.01-0.20 | 8.24E-02 | 1.67E-03 | FALSE | 0.05 | | -0.07-0.18 | 4.10E-01 | 0.56 | | 4.54E-01 |  |  |
|  | 0.1 | 0.19 | 0.08-0.29 | **6.36E-04**** | 1.67E-03 | TRUE | 0.11 | | -0.01-0.23 | 7.64E-02 | 0.85 | | 3.58E-01 |  |  |
|  | 0.2 | 0.16 | 0.05-0.27 | 3.08E-03* | 1.67E-03 | FALSE | 0.1 | | -0.02-0.22 | 1.04E-01 | 1.43 | | 2.31E-01 |  |  |
|  | 0.3 | 0.16 | 0.05-0.26 | 4.25E-03* | 1.67E-03 | FALSE | 0.09 | | -0.03-0.22 | 1.32E-01 | 2.23 | | 1.35E-01 |  |  |
|  | 0.4 | 0.16 | 0.05-0.27 | 3.27E-03* | 1.67E-03 | FALSE | 0.1 | | -0.02-0.22 | 1.03E-01 | 1.61 | | 2.04E-01 |  |  |
|  | 0.5 | 0.16 | 0.05-0.27 | 3.33E-03* | 1.67E-03 | FALSE | 0.09 | | -0.03-0.21 | 1.39E-01 | 2.27 | | 1.32E-01 |  |  |
|  | 1 | 0.15 | 0.04-0.26 | 6.04E-03* | 1.67E-03 | FALSE | 0.08 | | -0.04-0.21 | 1.79E-01 | 2.35 | | 1.25E-01 |  |  |

Note: All results were corrected for multiple testing using the Bonferroni correction (0.05/30 environments = p ≤1.67 x 10^-3^). * = significant, ** = significant after multiple testing. Sensitivity analysis was performed for all statistically significant results after multiple testing only. All regressions were calculated using STATA v12.1 (1).

# **Supplementary Table** **24**: All PRS results for USoc

| **Environment** | **Threshold**  **z-scored** | **Beta** | **95%CI** | **P-Value** | **Bonferroni Correction** | | | **Sensitivity** | | | | | **Interaction** | |
| --- | --- | --- | --- | --- | --- | --- | --- | --- | --- | --- | --- | --- | --- | --- |
|  |  |  |  |  | **0.05/30** | **statistically significant?** | **Beta** | | **95%CI** | **P-Value** | **Wald**  **chi-**  **square** | | | **p-value** |
| **SCZ** | | | | | | | | | | | | | | |
| **SES** | 0.01 | -0.02 | -0.05-0.01 | 1.45E-01 | 1.67E-03 | FALSE |  | | | | |  | | |
|  | 0.1 | -0.02 | -0.05-0.01 | 1.94E-01 | 1.67E-03 | FALSE |  |  |  |  |  |  |  |  |
|  | 0.2 | -0.02 | -0.05-0.01 | 1.66E-01 | 1.67E-03 | FALSE |  |  |  |  |  |  |  |  |
|  | 0.3 | -0.02 | -0.05-0.01 | 2.10E-01 | 1.67E-03 | FALSE |  |  |  |  |  |  |  |  |
|  | 0.4 | -0.02 | -0.05-0.01 | 2.33E-01 | 1.67E-03 | FALSE |  |  |  |  |  |  |  |  |
|  | 0.5 | -0.02 | -0.05-0.01 | 2.25E-01 | 1.67E-03 | FALSE |  |  |  |  |  |  |  |  |
|  | 1 | -0.02 | -0.05-0.01 | 2.70E-01 | 1.67E-03 | FALSE |  |  |  |  |  |  |  |  |
| **Number of Rooms** | 0.01 | -0.02 | -0.04-0.01 | 1.72E-01 | 1.67E-03 | FALSE |  | | | | |  | | |
|  | 0.1 | -0.02 | -0.04-0.01 | 1.36E-01 | 1.67E-03 | FALSE |  |  |  |  |  |  |  |  |
|  | 0.2 | -0.02 | -0.04-0.00 | 8.60E-02 | 1.67E-03 | FALSE |  |  |  |  |  |  |  |  |
|  | 0.3 | -0.02 | -0.04-0.00 | 9.32E-02 | 1.67E-03 | FALSE |  |  |  |  |  |  |  |  |
|  | 0.4 | -0.02 | -0.04-0.00 | 9.62E-02 | 1.67E-03 | FALSE |  |  |  |  |  |  |  |  |
|  | 0.5 | -0.02 | -0.04-0.00 | 8.94E-02 | 1.67E-03 | FALSE |  |  |  |  |  |  |  |  |
|  | 1 | -0.02 | -0.04-0.00 | 9.69E-02 | 1.67E-03 | FALSE |  |  |  |  |  |  |  |  |
| **Marital status** | 0.01 | 0.08 | 0.02-0.13 | 5.42E-03* | 1.67E-03 | FALSE |  | | | | |  | | |
|  | 0.1 | 0.1 | 0.04-0.15 | **7.96E-04**** | 1.67E-03 | TRUE |  |  |  |  |  |  |  |  |
|  | 0.2 | 0.09 | 0.03-0.15 | 1.71E-03* | 1.67E-03 | FALSE |  |  |  |  |  |  |  |  |
|  | 0.3 | 0.09 | 0.03-0.15 | 1.67E-03* | 1.67E-03 | FALSE |  |  |  |  |  |  |  |  |
|  | 0.4 | 0.09 | 0.04-0.15 | **1.21E-03**** | 1.67E-03 | TRUE |  |  |  |  |  |  |  |  |
|  | 0.5 | 0.09 | 0.04-0.15 | **1.13E-03**** | 1.67E-03 | TRUE |  |  |  |  |  |  |  |  |
|  | 1 | 0.09 | 0.03-0.15 | **1.63E-03**** | 1.67E-03 | TRUE |  |  |  |  |  |  |  |  |
| **Income** | 0.01 | -0.02 | -0.04-0.00 | 1.08E-01 | 1.67E-03 | FALSE |  | | | | |  | | |
|  | 0.1 | -0.02 | -0.04-0.00 | 6.61E-02 | 1.67E-03 | FALSE |  |  |  |  |  |  |  |  |
|  | 0.2 | -0.02 | -0.04-0.00 | 7.75E-02 | 1.67E-03 | FALSE |  |  |  |  |  |  |  |  |
|  | 0.3 | -0.02 | -0.04-0.00 | 7.76E-02 | 1.67E-03 | FALSE |  |  |  |  |  |  |  |  |
|  | 0.4 | -0.02 | -0.04-0.00 | 7.61E-02 | 1.67E-03 | FALSE |  |  |  |  |  |  |  |  |
|  | 0.5 | -0.02 | -0.04-0.00 | 7.76E-02 | 1.67E-03 | FALSE |  |  |  |  |  |  |  |  |
|  | 1 | -0.02 | -0.04-0.00 | 6.57E-02 | 1.67E-03 | FALSE |  |  |  |  |  |  |  |  |
| **Alcohol consumption** | 0.01 | 0 | -0.02-0.03 | 9.56E-01 | 1.67E-03 | FALSE |  | | | | |  | | |
|  | 0.1 | -0.01 | -0.03-0.02 | 6.22E-01 | 1.67E-03 | FALSE |  |  |  |  |  |  |  |  |
|  | 0.2 | -0.01 | -0.03-0.02 | 5.24E-01 | 1.67E-03 | FALSE |  |  |  |  |  |  |  |  |
|  | 0.3 | -0.01 | -0.03-0.02 | 5.97E-01 | 1.67E-03 | FALSE |  |  |  |  |  |  |  |  |
|  | 0.4 | -0.01 | -0.03-0.02 | 6.62E-01 | 1.67E-03 | FALSE |  |  |  |  |  |  |  |  |
|  | 0.5 | -0.01 | -0.03-0.02 | 5.80E-01 | 1.67E-03 | FALSE |  |  |  |  |  |  |  |  |
|  | 1 | -0.01 | -0.03-0.02 | 6.42E-01 | 1.67E-03 | FALSE |  |  |  |  |  |  |  |  |
| **Employment** | 0.01 | 0.16 | 0.04-0.27 | 7.81E-03* | 1.67E-03 | FALSE |  | | | | |  | | |
|  | 0.1 | 0.15 | 0.03-0.26 | 1.23E-02* | 1.67E-03 | FALSE |  |  |  |  |  |  |  |  |
|  | 0.2 | 0.15 | 0.04-0.27 | 1.01E-02* | 1.67E-03 | FALSE |  |  |  |  |  |  |  |  |
|  | 0.3 | 0.16 | 0.04-0.27 | 7.12E-03* | 1.67E-03 | FALSE |  |  |  |  |  |  |  |  |
|  | 0.4 | 0.15 | 0.03-0.26 | 1.10E-02* | 1.67E-03 | FALSE |  |  |  |  |  |  |  |  |
|  | 0.5 | 0.15 | 0.03-0.26 | 1.18E-02* | 1.67E-03 | FALSE |  |  |  |  |  |  |  |  |
|  | 1 | 0.15 | 0.04-0.27 | 8.50E-03* | 1.67E-03 | FALSE |  |  |  |  |  |  |  |  |
| **Tenure** | 0.01 | 0.05 | -0.17-0.28 | 6.48E-01 | 1.67E-03 | FALSE |  | | | | |  | | |
|  | 0.1 | 0.01 | -0.22-0.24 | 9.26E-01 | 1.67E-03 | FALSE |  |  |  |  |  |  |  |  |
|  | 0.2 | -0.02 | -0.25-0.22 | 8.80E-01 | 1.67E-03 | FALSE |  |  |  |  |  |  |  |  |
|  | 0.3 | 0.01 | -0.23-0.25 | 9.44E-01 | 1.67E-03 | FALSE |  |  |  |  |  |  |  |  |
|  | 0.4 | 0.03 | -0.2-0.26 | 8.11E-01 | 1.67E-03 | FALSE |  |  |  |  |  |  |  |  |
|  | 0.5 | 0.02 | -0.22-0.25 | 8.86E-01 | 1.67E-03 | FALSE |  |  |  |  |  |  |  |  |
|  | 1 | 0.01 | -0.22-0.25 | 9.01E-01 | 1.67E-03 | FALSE |  |  |  |  |  |  |  |  |
| **Finance Issues** | 0.01 | 0.12 | 0.03-0.20 | 7.99E-03* | 1.67E-03 | FALSE |  | | | | |  | | |
|  | 0.1 | 0.13 | 0.04-0.21 | 3.85E-03* | 1.67E-03 | FALSE |  |  |  |  |  |  |  |  |
|  | 0.2 | 0.13 | 0.05-0.22 | 2.75E-03* | 1.67E-03 | FALSE |  |  |  |  |  |  |  |  |
|  | 0.3 | 0.13 | 0.04-0.22 | 3.55E-03* | 1.67E-03 | FALSE |  |  |  |  |  |  |  |  |
|  | 0.4 | 0.13 | 0.05-0.22 | 2.85E-03* | 1.67E-03 | FALSE |  |  |  |  |  |  |  |  |
|  | 0.5 | 0.14 | 0.05-0.22 | 2.29E-03* | 1.67E-03 | FALSE |  |  |  |  |  |  |  |  |
|  | 1 | 0.14 | 0.05-0.22 | 1.90E-03* | 1.67E-03 | FALSE |  |  |  |  |  |  |  |  |
| **Education** | 0.01 | 0 | -0.36-0.37 | 9.94E-01 | 1.67E-03 | FALSE |  | | | | |  | | |
|  | 0.1 | -0.04 | -0.41-0.33 | 8.23E-01 | 1.67E-03 | FALSE |  |  |  |  |  |  |  |  |
|  | 0.2 | -0.04 | -0.41-0.33 | 8.43E-01 | 1.67E-03 | FALSE |  |  |  |  |  |  |  |  |
|  | 0.3 | -0.06 | -0.43-0.32 | 7.71E-01 | 1.67E-03 | FALSE |  |  |  |  |  |  |  |  |
|  | 0.4 | -0.05 | -0.42-0.32 | 7.86E-01 | 1.67E-03 | FALSE |  |  |  |  |  |  |  |  |
|  | 0.5 | -0.05 | -0.42-0.32 | 7.88E-01 | 1.67E-03 | FALSE |  |  |  |  |  |  |  |  |
|  | 1 | -0.05 | -0.42-0.32 | 7.90E-01 | 1.67E-03 | FALSE |  |  |  |  |  |  |  |  |
| **MDD** | | | | | | | | | | | | | | |
| **SES** | 0.01 | -0.03 | -0.06--0.00 | 2.62E-02 | 1.67E-03 | FALSE |  | | | | |  | | |
|  | 0.1 | -0.02 | -0.05-0.01 | 1.39E-01 | 1.67E-03 | FALSE |  |  |  |  |  |  |  |  |
|  | 0.2 | -0.01 | -0.04-0.02 | 3.72E-01 | 1.67E-03 | FALSE |  |  |  |  |  |  |  |  |
|  | 0.3 | -0.01 | -0.04-0.02 | 4.00E-01 | 1.67E-03 | FALSE |  |  |  |  |  |  |  |  |
|  | 0.4 | -0.01 | -0.04-0.02 | 3.76E-01 | 1.67E-03 | FALSE |  |  |  |  |  |  |  |  |
|  | 0.5 | -0.01 | -0.04-0.02 | 4.94E-01 | 1.67E-03 | FALSE |  |  |  |  |  |  |  |  |
|  | 1 | -0.01 | -0.04-0.02 | 4.17E-01 | 1.67E-03 | FALSE |  |  |  |  |  |  |  |  |
| **Number of Rooms** | 0.01 | -0.04 | -0.07--0.02 | **4.36E-04**** | 1.67E-03 | TRUE | -0.04 | | -0.07--0.02 | 4.78E-04 | 1.98 | | | 1.60E-01 |
|  | 0.1 | -0.04 | -0.06--0.01 | 1.90E-03* | 1.67E-03 | FALSE | -0.04 | | -0.06--0.01 | 3.22E-03 | 0.92 | | | 3.37E-01 |
|  | 0.2 | -0.02 | -0.05--0.00 | 4.06E-02* | 1.67E-03 | FALSE | -0.02 | | -0.05-0.00 | 7.23E-02 | 0.21 | | | 6.47E-01 |
|  | 0.3 | -0.03 | -0.05--0.00 | 3.59E-02* | 1.67E-03 | FALSE | -0.02 | | -0.05-0.00 | 6.93E-02 | 0.15 | | | 7.01E-01 |
|  | 0.4 | -0.02 | -0.05--0.00 | 4.61E-02* | 1.67E-03 | FALSE | -0.02 | | -0.04-0.00 | 1.02E-01 | 0.02 | | | 8.86E-01 |
|  | 0.5 | -0.03 | -0.05--0.00 | 3.46E-02* | 1.67E-03 | FALSE | -0.02 | | -0.05-0.00 | 8.45E-02 | 0.01 | | | 9.35E-01 |
|  | 1 | -0.03 | -0.05--0.00 | 2.74E-02* | 1.67E-03 | FALSE | -0.02 | | -0.05-0.00 | 6.81E-02 | 0.01 | | | 9.43E-01 |
| **Marital status** | 0.01 | 0.08 | 0.03-0.14 | 2.38E-03 | 1.67E-03 | FALSE |  | | | | |  | | |
|  | 0.1 | 0.05 | -0.01-0.10 | 1.03E-01 | 1.67E-03 | FALSE |  |  |  |  |  |  |  |  |
|  | 0.2 | 0.02 | -0.04-0.07 | 5.70E-01 | 1.67E-03 | FALSE |  |  |  |  |  |  |  |  |
|  | 0.3 | 0.01 | -0.04-0.07 | 6.40E-01 | 1.67E-03 | FALSE |  |  |  |  |  |  |  |  |
|  | 0.4 | 0.01 | -0.04-0.07 | 6.64E-01 | 1.67E-03 | FALSE |  |  |  |  |  |  |  |  |
|  | 0.5 | 0.01 | -0.04-0.07 | 6.00E-01 | 1.67E-03 | FALSE |  |  |  |  |  |  |  |  |
|  | 1 | 0.02 | -0.04-0.07 | 5.18E-01 | 1.67E-03 | FALSE |  |  |  |  |  |  |  |  |
| **Income** | 0.01 | -0.03 | -0.05--0.01 | **6.72E-04**** | 1.67E-03 | TRUE | -0.04 | | -0.06--0.02 | 2.98E-04 | 2.86 | | | 9.08E-02 |
|  | 0.1 | -0.02 | -0.04--0.00 | 1.83E-02* | 1.67E-03 | FALSE | -0.03 | | -0.05--0.01 | 1.09E-02 | 1.22 | | | 2.69E-01 |
|  | 0.2 | -0.02 | -0.04--0.00 | 2.41E-02* | 1.67E-03 | FALSE | -0.03 | | -0.05--0.00 | 1.54E-02 | 0.86 | | | 3.53E-01 |
|  | 0.3 | -0.02 | -0.04--0.00 | 4.11E-02* | 1.67E-03 | FALSE | -0.02 | | -0.04--0.00 | 3.03E-02 | 0.46 | | | 5.00E-01 |
|  | 0.4 | -0.02 | -0.04-0.00 | 5.57E-02* | 1.67E-03 | FALSE | -0.02 | | -0.04--0.00 | 4.51E-02 | 0.32 | | | 5.72E-01 |
|  | 0.5 | -0.02 | -0.04--0.00 | 4.75E-02* | 1.67E-03 | FALSE | -0.02 | | -0.04--0.00 | 3.79E-02 | 0.36 | | | 5.48E-01 |
|  | 1 | -0.02 | -0.04--0.00 | 4.31E-02* | 1.67E-03 | FALSE | -0.02 | | -0.04--0.00 | 2.89E-02 | 0.61 | | | 4.34E-01 |
| **Alcohol consumption** | 0.01 | 0.01 | -0.01-0.04 | 3.29E-01 | 1.67E-03 | FALSE |  | | | | |  | | |
|  | 0.1 | -0.01 | -0.03-0.02 | 4.74E-01 | 1.67E-03 | FALSE |  |  |  |  |  |  |  |  |
|  | 0.2 | -0.01 | -0.03-0.02 | 5.03E-01 | 1.67E-03 | FALSE |  |  |  |  |  |  |  |  |
|  | 0.3 | 0 | -0.03-0.02 | 7.37E-01 | 1.67E-03 | FALSE |  |  |  |  |  |  |  |  |
|  | 0.4 | -0.01 | -0.03-0.02 | 6.45E-01 | 1.67E-03 | FALSE |  |  |  |  |  |  |  |  |
|  | 0.5 | 0 | -0.03-0.02 | 7.07E-01 | 1.67E-03 | FALSE |  |  |  |  |  |  |  |  |
|  | 1 | -0.01 | -0.03-0.02 | 6.25E-01 | 1.67E-03 | FALSE |  |  |  |  |  |  |  |  |
| **Employment** | 0.01 | 0.2 | 0.09-0.32 | **5.50E-04**** | 1.67E-03 | TRUE | 0.18 | | 0.07-0.30 | 1.68E-03 | 3.33 | | | 6.79E-02 |
|  | 0.1 | 0.21 | 0.10-0.33 | **2.63E-04**** | 1.67E-03 | TRUE | 0.18 | | 0.06-0.29 | 2.34E-03 | 0.7 | | | 4.04E-01 |
|  | 0.2 | 0.23 | 0.12-0.35 | **5.95E-05**** | 1.67E-03 | TRUE | 0.21 | | 0.09-0.32 | 4.29E-04 | 0.67 | | | 4.14E-01 |
|  | 0.3 | 0.22 | 0.10-0.33 | **2.20E-04**** | 1.67E-03 | TRUE | 0.17 | | 0.06-0.29 | 2.94E-03 | 0.14 | | | 7.12E-01 |
|  | 0.4 | 0.21 | 0.09-0.32 | **3.86E-04**** | 1.67E-03 | TRUE | 0.17 | | 0.05-0.28 | 4.29E-03 | 0.28 | | | 5.97E-01 |
|  | 0.5 | 0.21 | 0.09-0.32 | **3.98E-04**** | 1.67E-03 | TRUE | 0.16 | | 0.05-0.28 | 5.18E-03 | 0.17 | | | 6.80E-01 |
|  | 1 | 0.2 | 0.09-0.31 | **5.61E-04**** | 1.67E-03 | TRUE | 0.16 | | 0.05-0.28 | 5.07E-03 | 0.4 | | | 5.30E-01 |
| **Tenure** | 0.01 | 0.13 | -0.10-0.37 | 2.51E-01 | 1.67E-03 | FALSE |  | | | | |  | | |
|  | 0.1 | 0.13 | -0.10-0.35 | 2.70E-01 | 1.67E-03 | FALSE |  |  |  |  |  |  |  |  |
|  | 0.2 | 0.1 | -0.13-0.33 | 4.07E-01 | 1.67E-03 | FALSE |  |  |  |  |  |  |  |  |
|  | 0.3 | 0.09 | -0.15-0.33 | 4.58E-01 | 1.67E-03 | FALSE |  |  |  |  |  |  |  |  |
|  | 0.4 | 0.09 | -0.14-0.32 | 4.51E-01 | 1.67E-03 | FALSE |  |  |  |  |  |  |  |  |
|  | 0.5 | 0.1 | -0.14-0.33 | 4.15E-01 | 1.67E-03 | FALSE |  |  |  |  |  |  |  |  |
|  | 1 | 0.09 | -0.14-0.32 | 4.29E-01 | 1.67E-03 | FALSE |  |  |  |  |  |  |  |  |
| **Finance Issues** | 0.01 | 0.24 | 0.16-0.33 | **2.91E-08**** | 1.67E-03 | TRUE | 0.23 | | 0.14-0.32 | 9.91E-07 | 2.33 | | | 1.27E-01 |
|  | 0.1 | 0.22 | 0.14-0.31 | **3.48E-07**** | 1.67E-03 | TRUE | 0.19 | | 0.10-0.28 | 5.67E-05 | 0.15 | | | 6.95E-01 |
|  | 0.2 | 0.22 | 0.14-0.31 | **4.09E-07**** | 1.67E-03 | TRUE | 0.19 | | 0.10-0.28 | 5.40E-05 | 0.13 | | | 7.15E-01 |
|  | 0.3 | 0.23 | 0.15-0.32 | **1.13E-07**** | 1.67E-03 | TRUE | 0.19 | | 0.10-0.28 | 4.54E-05 | 0.03 | | | 8.71E-01 |
|  | 0.4 | 0.23 | 0.15-0.32 | **9.79E-08**** | 1.67E-03 | TRUE | 0.19 | | 0.10-0.28 | 4.49E-05 | 0.02 | | | 9.02E-01 |
|  | 0.5 | 0.23 | 0.14-0.32 | **1.52E-07**** | 1.67E-03 | TRUE | 0.19 | | 0.10-0.28 | 6.60E-05 | 0.02 | | | 8.99E-01 |
|  | 1 | 0.23 | 0.15-0.32 | **1.36E-07**** | 1.67E-03 | TRUE | 0.19 | | 0.10-0.28 | 5.42E-05 | 0.02 | | | 8.80E-01 |
| **Education** | 0.01 | 0.2 | -0.17-0.57 | 2.96E-01 | 1.67E-03 | FALSE |  | | | | |  | | |
|  | 0.1 | 0.1 | -0.27-0.47 | 5.93E-01 | 1.67E-03 | FALSE |  |  |  |  |  |  |  |  |
|  | 0.2 | 0.09 | -0.27-0.46 | 6.13E-01 | 1.67E-03 | FALSE |  |  |  |  |  |  |  |  |
|  | 0.3 | 0.09 | -0.27-0.46 | 6.24E-01 | 1.67E-03 | FALSE |  |  |  |  |  |  |  |  |
|  | 0.4 | 0.09 | -0.27-0.46 | 6.11E-01 | 1.67E-03 | FALSE |  |  |  |  |  |  |  |  |
|  | 0.5 | 0.09 | -0.27-0.46 | 6.18E-01 | 1.67E-03 | FALSE |  |  |  |  |  |  |  |  |
|  | 1 | 0.1 | -0.27-0.46 | 6.03E-01 | 1.67E-03 | FALSE |  |  |  |  |  |  |  |  |

Note: All results were corrected for multiple testing using the Bonferroni correction (0.05/30 environments = p ≤1.67 x 10^-3^). * = significant, ** = significant after multiple testing. Sensitivity analysis was performed for all statistically significant results after multiple testing only, except for the one significant SCZ finding due to the lack of available SCZ diagnosis and symptoms in the USoc dataset. All regressions were calculated using STATA v12.1 (1).

# **Supplementary Tables** **25**: NCDS environmental risk factor correlation matrix

Correlations have been run for potential indicators of socio-economic status (SES), including number of bedrooms in the family home, unemployment and tenure

| Environment |  | z_Rooms_23 | z_Rooms_33 | z_Rooms_42 | z_Rooms_46 | z_Rooms_50 | z_Rooms_55 |
| --- | --- | --- | --- | --- | --- | --- | --- |
| z_SES_23 | *r* | 0.0264 | 0.1421 | 0.1408 | 0.2558 | 0.2628 | 0.2994 |
|  | p | 0.1153 | 0 | 0 | 0 | 0 | 0 |
| z_SES_33 | *r* | 0.0364 | 0.0996 | 0.1341 | 0.2488 | 0.2552 | 0.2036 |
|  | p | 0.0243 | 0 | 0 | 0 | 0 | 0 |
| z_SES_42 | *r* | 0.0511 | 0.1169 | 0.15 | 0.276 | 0.2652 | 0.2698 |
|  | p | 0.0016 | 0 | 0 | 0 | 0 | 0 |
| z_SES_46 | *r* | 0.0607 | 0.1287 | 0.155 | 0.2725 | 0.2704 | 0.304 |
|  | p | 0.0002 | 0 | 0 | 0 | 0 | 0 |
| z_SES_50 | *r* | 0.0374 | 0.1192 | 0.1591 | 0.2735 | 0.2822 | 0.296 |
|  | p | 0.0269 | 0 | 0 | 0 | 0 | 0 |
| z_SES_55 | *r* | 0.0588 | 0.1066 | 0.1355 | 0.2662 | 0.2761 | 0.2936 |
|  | p | 0.0012 | 0 | 0 | 0 | 0 | 0 |

Note: *r* = correlation coefficient, p= p-value, rooms = number of bedrooms, SES = socio-economic status, polytomous/continuous variables were z-scored

| Environment |  | z_Rooms_23 | z_Rooms_42 | z_Rooms_50 | z_Rooms_55 |
| --- | --- | --- | --- | --- | --- |
| Unemployment_23 | *r* | 0.0292 | -0.0679 | -0.0979 | -0.0876 |
|  | p | 0.21 | 0.0038 | 0.0001 | 0.1526 |
| Unemployment_42 | *r* | -0.0115 | -0.0165 | -0.0549 | -0.1056 |
|  | p | 0.452 | 0.2385 | 0.0002 | 0.004 |
| Unemployment_50 | *r* | 0.0024 | -0.0381 | -0.0586 | -0.0732 |
|  | p | 0.8827 | 0.0108 | 0.0001 | 0.0578 |
| Unemployment_55 | *r* | -0.0063 | -0.0563 | -0.0771 | -0.1098 |
|  | p | 0.7048 | 0.0003 | 0 | 0.0032 |

Note: *r* = correlation coefficient, p= p-value, rooms = number of bedrooms, polytomous/continuous variables were z-scored

| Environment |  | tenure_23 | tenure_42 | tenure_50 | tenure_55 |
| --- | --- | --- | --- | --- | --- |
| Unemployment_23 | *r* | 0.1423 | 0.1196 | 0.1004 | 0.1218 |
|  | p | 0 | 0 | 0 | 0.0002 |
| Unemployment_42 | *r* | 0.0956 | 0.257 | 0.2059 | 0.1986 |
|  | p | 0 | 0 | 0 | 0 |
| Unemployment_50 | *r* | 0.1005 | 0.2179 | 0.2289 | 0.2226 |
|  | p | 0 | 0 | 0 | 0 |
| Unemployment_55 | *r* | 0.0817 | 0.1837 | 0.1922 | 0.2129 |
|  | p | 0.0001 | 0 | 0 | 0 |

Note: *r* = correlation coefficient, p= p-value

| Environment |  | z_SES_23 | z_SES_42 | z_SES_50 | z_SES_55 |
| --- | --- | --- | --- | --- | --- |
| Unemployment_23 | *r* | -0.1567 | -0.0784 | -0.0898 | -0.0514 |
|  | p | 0 | 0.0022 | 0.0008 | 0.0753 |
| Unemployment_42 | *r* | -0.0846 | . | -0.1173 | -0.1075 |
|  | p | 0 | . | 0 | 0 |
| Unemployment_50 | *r* | -0.091 | -0.0885 | . | -0.0651 |
|  | p | 0 | 0 | . | 0.0002 |
| Unemployment_55 | *r* | -0.095 | -0.0753 | -0.0857 | . |
|  | p | 0 | 0 | 0 | . |

Note: *r* = correlation coefficient, p= p-value. SES = socio-economic status, some missing values due to insufficient responses, polytomous/continuous variables were z-scored

| Environment |  | tenure_23 | tenure_33 | tenure_42 | tenure_46 | tenure_50 | tenure_55 |
| --- | --- | --- | --- | --- | --- | --- | --- |
| z_SES_23 | *r* | -0.1391 | -0.219 | -0.1978 | -0.1762 | -0.1684 | -0.1899 |
|  | p | 0 | 0 | 0 | 0 | 0 | 0 |
| z_SES_33 | *r* | -0.0556 | -0.2291 | -0.233 | -0.1917 | -0.1938 | -0.1981 |
|  | p | 0.0081 | 0 | 0 | 0 | 0 | 0 |
| z_SES_42 | *r* | -0.0487 | -0.17 | -0.2061 | -0.1593 | -0.1711 | -0.1658 |
|  | p | 0.0202 | 0 | 0 | 0 | 0 | 0 |
| z_SES_46 | *r* | -0.0469 | -0.1691 | -0.1661 | -0.1435 | -0.1424 | -0.1449 |
|  | p | 0.0285 | 0 | 0 | 0 | 0 | 0 |
| z_SES_50 | *r* | -0.0343 | -0.1771 | -0.187 | -0.1655 | -0.1788 | -0.1867 |
|  | p | 0.1179 | 0 | 0 | 0 | 0 | 0 |
| z_SES_55 | *r* | -0.0219 | -0.1661 | -0.1564 | -0.1136 | -0.136 | -0.1958 |
|  | p | 0.3518 | 0 | 0 | 0 | 0 | 0 |

Note: *r* = correlation coefficient, p= p-value, SES = socio-economic status, polytomous/continuous variables were z-scored

| Environment |  | tenure_23 | tenure_33 | tenure_42 | tenure_46 | tenure_50 | tenure_55 |
| --- | --- | --- | --- | --- | --- | --- | --- |
| z_Rooms_23 | *r* | -0.1674 | -0.0509 | -0.0221 | -0.0111 | -0.0022 | -0.0433 |
|  | p | -0.1692 | -0.0511 | -0.0225 | -0.0114 | -0.0029 | -0.0448 |
| z_Rooms_33 | *r* | 0 | 0.0017 | 0.143 | 0.4655 | 0.8565 | 0.0373 |
|  | p | -0.1886 | -0.228 | -0.115 | -0.115 | -0.1145 | -0.1368 |
| z_Rooms_42 | *r* | 0 | 0 | 0 | 0 | 0 | 0 |
|  | p | -0.1317 | -0.1633 | -0.1878 | -0.1413 | -0.1484 | -0.1381 |
| z_Rooms_46 | *r* | 0 | 0 | 0 | 0 | 0 | 0 |
|  | p | -0.1823 | -0.2496 | -0.2614 | -0.2673 | -0.2374 | -0.2616 |
| z_Rooms_50 | *r* | 0 | 0 | 0 | 0 | 0 | 0 |
|  | p | -0.1907 | -0.2553 | -0.2472 | -0.2515 | -0.2842 | -0.2765 |
| z_Rooms_55 | *r* | 0 | 0 | 0 | 0 | 0 | 0 |
|  | p | -0.1334 | -0.1801 | -0.2249 | -0.2704 | -0.2145 | -0.3374 |

Note: *r* = correlation coefficient, p= p-value, rooms = number of bedrooms, polytomous/continuous variables were z-scored

# **Supplementary Tables** **26**: USoc environmental risk factor correlation matrix

Correlations have been run for potential indicators of socio-economic status (SES), including number of bedrooms in the family home, unemployment, tenure, income and financial difficulties

| Environment |  | z_Rooms_1 | z_Rooms_2 | z_Rooms_3 | z_Rooms_4 | z_Rooms_5 | z_Rooms_6 | z_Rooms_7 | z_Rooms_8 | z_Rooms_9 |
| --- | --- | --- | --- | --- | --- | --- | --- | --- | --- | --- |
| z_Income_1 | *r* | 0.1186 | 0.121 | 0.1274 | 0.1424 | 0.1571 | 0.1724 | 0.175 | 0.1726 | 0.1766 |
|  | p | 0 | 0 | 0 | 0 | 0 | 0 | 0 | 0 | 0 |
| z_Income_2 | *r* | 0.1332 | 0.1302 | 0.1393 | 0.155 | 0.1671 | 0.1821 | 0.1841 | 0.1934 | 0.2057 |
|  | p | 0 | 0 | 0 | 0 | 0 | 0 | 0 | 0 | 0 |
| z_Income_3 | *r* | 0.1163 | 0.1131 | 0.1185 | 0.1404 | 0.1455 | 0.1598 | 0.1621 | 0.1749 | 0.1829 |
|  | p | 0 | 0 | 0 | 0 | 0 | 0 | 0 | 0 | 0 |
| z_Income_4 | *r* | 0.1288 | 0.1257 | 0.1353 | 0.1464 | 0.1531 | 0.1644 | 0.1714 | 0.1835 | 0.19 |
|  | p | 0 | 0 | 0 | 0 | 0 | 0 | 0 | 0 | 0 |
| z_Income_5 | *r* | 0.118 | 0.1187 | 0.1248 | 0.1362 | 0.1481 | 0.1681 | 0.1705 | 0.1751 | 0.1857 |
|  | p | 0 | 0 | 0 | 0 | 0 | 0 | 0 | 0 | 0 |
| z_Income_6 | *r* | 0.1507 | 0.1399 | 0.1497 | 0.1601 | 0.1721 | 0.1841 | 0.1971 | 0.1909 | 0.1982 |
|  | p | 0 | 0 | 0 | 0 | 0 | 0 | 0 | 0 | 0 |
| z_Income_7 | p | 0.1429 | 0.132 | 0.1412 | 0.1467 | 0.1586 | 0.1759 | 0.1723 | 0.1813 | 0.1847 |
|  | *r* | 0 | 0 | 0 | 0 | 0 | 0 | 0 | 0 | 0 |
| z_Income_8 | p | 0.1484 | 0.146 | 0.1606 | 0.1615 | 0.1725 | 0.1791 | 0.1792 | 0.1839 | 0.1913 |
|  | p | 0 | 0 | 0 | 0 | 0 | 0 | 0 | 0 | 0 |
| z_Income_9 | *r* | 0.1281 | 0.1288 | 0.147 | 0.1509 | 0.1536 | 0.1705 | 0.1723 | 0.1808 | 0.1881 |
|  | p | 0 | 0 | 0 | 0 | 0 | 0 | 0 | 0 | 0 |

Note: *r* = correlation coefficient, p= p-value, rooms = number of bedrooms, polytomous/continuous variables were z-scored

| Environment |  | z_SES_1 | z_SES_2 | z_SES_3 | z_SES_4 | z_SES_5 | z_SES_6 | z_SES_7 | z_SES_8 | z_SES_9 |
| --- | --- | --- | --- | --- | --- | --- | --- | --- | --- | --- |
| z_Income_1 | *r* | 0.4277 | 0.4034 | 0.4015 | 0.3968 | 0.3852 | 0.3655 | 0.3555 | 0.349 | 0.3581 |
|  | p | 0 | 0 | 0 | 0 | 0 | 0 | 0 | 0 | 0 |
| z_Income_2 | *r* | 0.3958 | 0.4313 | 0.4251 | 0.4272 | 0.4035 | 0.3786 | 0.3617 | 0.3603 | 0.3456 |
|  | p | 0 | 0 | 0 | 0 | 0 | 0 | 0 | 0 | 0 |
| z_Income_3 | *r* | 0.3818 | 0.4078 | 0.4241 | 0.4097 | 0.3939 | 0.3734 | 0.3424 | 0.3498 | 0.3286 |
|  | p | 0 | 0 | 0 | 0 | 0 | 0 | 0 | 0 | 0 |
| z_Income_4 | *r* | 0.365 | 0.3894 | 0.4025 | 0.4272 | 0.4131 | 0.3853 | 0.3625 | 0.3651 | 0.3548 |
|  | p | 0 | 0 | 0 | 0 | 0 | 0 | 0 | 0 | 0 |
| z_Income_5 | *r* | 0.3954 | 0.3927 | 0.3972 | 0.4105 | 0.4347 | 0.41 | 0.3881 | 0.3731 | 0.3719 |
|  | p | 0 | 0 | 0 | 0 | 0 | 0 | 0 | 0 | 0 |
| z_Income_6 | *r* | 0.3608 | 0.3753 | 0.3817 | 0.3878 | 0.3992 | 0.4066 | 0.3834 | 0.3764 | 0.3807 |
|  | p | 0 | 0 | 0 | 0 | 0 | 0 | 0 | 0 | 0 |
| z_Income_7 | p | 0.3582 | 0.3714 | 0.3752 | 0.3748 | 0.3791 | 0.372 | 0.3882 | 0.3758 | 0.3749 |
|  | *r* | 0 | 0 | 0 | 0 | 0 | 0 | 0 | 0 | 0 |
| z_Income_8 | p | 0.347 | 0.3421 | 0.3556 | 0.3404 | 0.3492 | 0.3397 | 0.3435 | 0.3845 | 0.3789 |
|  | p | 0 | 0 | 0 | 0 | 0 | 0 | 0 | 0 | 0 |
| z_Income_9 | *r* | 0.3014 | 0.3223 | 0.3397 | 0.3337 | 0.3443 | 0.3409 | 0.3407 | 0.3609 | 0.4064 |
|  | p | 0 | 0 | 0 | 0 | 0 | 0 | 0 | 0 | 0 |

Note: *r* = correlation coefficient, p= p-value, SES= socio-economic status, polytomous/continuous variables were z-scored

| Environment |  | z_SES_1 | z_SES_2 | z_SES_3 | z_SES_4 | z_SES_5 | z_SES_6 | z_SES_7 | z_SES_8 | z_SES_9 |
| --- | --- | --- | --- | --- | --- | --- | --- | --- | --- | --- |
| z_Rooms_1 | *r* | 0.1396 | 0.1478 | 0.1284 | 0.133 | 0.1408 | 0.1303 | 0.1391 | 0.1405 | 0.1405 |
|  | p | 0 | 0 | 0 | 0 | 0 | 0 | 0 | 0 |  |
| z_Rooms_2 | *r* | 0.142 | 0.1653 | 0.1464 | 0.1412 | 0.147 | 0.1428 | 0.1514 | 0.1557 | 0.1507 |
|  | p | 0 | 0 | 0 | 0 | 0 | 0 | 0 | 0 | 0 |
| z_Rooms_3 | *r* | 0.1539 | 0.1734 | 0.1565 | 0.1544 | 0.1559 | 0.1441 | 0.1506 | 0.1523 | 0.154 |
|  | p | 0 | 0 | 0 | 0 | 0 | 0 | 0 | 0 | 0 |
| z_Rooms_4 | *r* | 0.1588 | 0.1808 | 0.1716 | 0.1597 | 0.1695 | 0.1589 | 0.1631 | 0.1639 | 0.1566 |
|  | p | 0 | 0 | 0 | 0 | 0 | 0 | 0 | 0 | 0 |
| z_Rooms_5 | *r* | 0.1783 | 0.1865 | 0.1831 | 0.1737 | 0.1803 | 0.1691 | 0.1757 | 0.1792 | 0.1777 |
|  | p | 0 | 0 | 0 | 0 | 0 | 0 | 0 | 0 | 0 |
| z_Rooms_6 | *r* | 0.1949 | 0.2001 | 0.194 | 0.1944 | 0.1945 | 0.1773 | 0.1851 | 0.1842 | 0.1924 |
|  | p | 0 | 0 | 0 | 0 | 0 | 0 | 0 | 0 | 0 |
| z_Rooms_7 | p | 0.2021 | 0.2076 | 0.1956 | 0.2018 | 0.1901 | 0.1833 | 0.1826 | 0.1761 | 0.1809 |
|  | *r* | 0 | 0 | 0 | 0 | 0 | 0 | 0 | 0 | 0 |
| z_Rooms_8 | p | 0.2144 | 0.2133 | 0.2065 | 0.2117 | 0.1926 | 0.1801 | 0.1815 | 0.174 | 0.1795 |
|  | p | 0 | 0 | 0 | 0 | 0 | 0 | 0 | 0 | 0 |
| z_Rooms_9 | *r* | 0.2161 | 0.2136 | 0.2081 | 0.2093 | 0.2008 | 0.1881 | 0.1912 | 0.1947 | 0.1992 |
|  | p | 0 | 0 | 0 | 0 | 0 | 0 | 0 | 0 | 0 |

Note: *r* = correlation coefficient, p= p-value, rooms = number of bedrooms, SES= socio-economic status, polytomous/continuous variables were z-scored

| Environment |  | Finance_1 | Finance_2 | Finance_3 | Finance_4 | Finance_5 | Finance_6 | Finance_7 | Finance_8 | Finance_9 |
| --- | --- | --- | --- | --- | --- | --- | --- | --- | --- | --- |
| Employment_1 | *r* | 0.2159 | 0.1948 | 0.1763 | 0.1719 | 0.1737 | 0.1638 | 0.165 | 0.1518 | 0.149 |
|  | p | 0 | 0 | 0 | 0 | 0 | 0 | 0 | 0 | 0 |
| Employment_2 | *r* | 0.1874 | 0.2359 | 0.1803 | 0.1649 | 0.1687 | 0.1443 | 0.1327 | 0.1472 | 0.1338 |
|  | p | 0 | 0 | 0 | 0 | 0 | 0 | 0 | 0 | 0 |
| Employment_3 | *r* | 0.1935 | 0.1919 | 0.2162 | 0.1935 | 0.1722 | 0.1454 | 0.1648 | 0.1639 | 0.1301 |
|  | p | 0 | 0 | 0 | 0 | 0 | 0 | 0 | 0 | 0 |
| Employment_4 | *r* | 0.1617 | 0.1766 | 0.2092 | 0.2199 | 0.1878 | 0.1477 | 0.177 | 0.1413 | 0.1527 |
|  | p | 0 | 0 | 0 | 0 | 0 | 0 | 0 | 0 | 0 |
| Employment_5 | *r* | 0.1671 | 0.1637 | 0.1983 | 0.1942 | 0.2025 | 0.1482 | 0.1702 | 0.1465 | 0.1343 |
|  | p | 0 | 0 | 0 | 0 | 0 | 0 | 0 | 0 | 0 |
| Employment_6 | *r* | 0.1424 | 0.1801 | 0.2131 | 0.1902 | 0.1752 | 0.1967 | 0.1889 | 0.1605 | 0.1506 |
|  | p | 0 | 0 | 0 | 0 | 0 | 0 | 0 | 0 | 0 |
| Employment_7 | p | 0.1431 | 0.1851 | 0.1964 | 0.1762 | 0.1726 | 0.1665 | 0.2156 | 0.1727 | 0.1369 |
|  | *r* | 0 | 0 | 0 | 0 | 0 | 0 | 0 | 0 | 0 |
| Employment_8 | p | 0.144 | 0.1735 | 0.1805 | 0.1819 | 0.1829 | 0.1854 | 0.2043 | 0.1893 | 0.1572 |
|  | p | 0 | 0 | 0 | 0 | 0 | 0 | 0 | 0 | 0 |
| Employment_9 | *r* | 0.1372 | 0.1554 | 0.1954 | 0.1866 | 0.1853 | 0.1694 | 0.2192 | 0.1804 | 0.1822 |
|  | p | 0 | 0 | 0 | 0 | 0 | 0 | 0 | 0 | 0 |

Note: *r* = correlation coefficient, p= p-value, finance = financial difficulties

| Environment |  | Finance_1 | Finance_2 | Finance_3 | Finance_4 | Finance_5 | Finance_6 | Finance_7 | Finance_8 | Finance_9 |
| --- | --- | --- | --- | --- | --- | --- | --- | --- | --- | --- |
| tenure_1 | *r* | 0.218 | 0.1868 | 0.1998 | 0.2163 | 0.1759 | 0.1561 | 0.1811 | 0.1732 | 0.1566 |
|  | p | 0 | 0 | 0 | 0 | 0 | 0 | 0 | 0 | 0 |
| tenure_2 | *r* | 0.2117 | 0.181 | 0.1821 | 0.1948 | 0.1704 | 0.1495 | 0.1654 | 0.1635 | 0.1392 |
|  | p | 0 | 0 | 0 | 0 | 0 | 0 | 0 | 0 | 0 |
| tenure_3 | *r* | 0.2066 | 0.1792 | 0.1869 | 0.1965 | 0.1703 | 0.1474 | 0.165 | 0.1709 | 0.1443 |
|  | p | 0 | 0 | 0 | 0 | 0 | 0 | 0 | 0 | 0 |
| tenure_4 | *r* | 0.2102 | 0.1827 | 0.1908 | 0.2056 | 0.1759 | 0.152 | 0.1684 | 0.163 | 0.1467 |
|  | p | 0 | 0 | 0 | 0 | 0 | 0 | 0 | 0 | 0 |
| tenure_5 | *r* | 0.2139 | 0.1887 | 0.1939 | 0.2075 | 0.1778 | 0.1563 | 0.1704 | 0.1686 | 0.1399 |
|  | p | 0 | 0 | 0 | 0 | 0 | 0 | 0 | 0 | 0 |
| tenure_6 | *r* | 0.2263 | 0.1908 | 0.1827 | 0.2092 | 0.1808 | 0.157 | 0.1683 | 0.1694 | 0.1502 |
|  | p | 0 | 0 | 0 | 0 | 0 | 0 | 0 | 0 | 0 |
| tenure_7 | p | 0.2216 | 0.1924 | 0.1767 | 0.2198 | 0.1819 | 0.1559 | 0.1814 | 0.1681 | 0.1406 |
|  | *r* | 0 | 0 | 0 | 0 | 0 | 0 | 0 | 0 | 0 |
| tenure_8 | p | 0.2171 | 0.185 | 0.1843 | 0.2237 | 0.1881 | 0.1597 | 0.1881 | 0.1775 | 0.1456 |
|  | p | 0 | 0 | 0 | 0 | 0 | 0 | 0 | 0 | 0 |
| tenure_9 | *r* | 0.2137 | 0.1838 | 0.1931 | 0.2164 | 0.1977 | 0.175 | 0.1884 | 0.1839 | 0.1531 |
|  | p | 0 | 0 | 0 | 0 | 0 | 0 | 0 | 0 | 0 |

Note: *r* = correlation coefficient, p= p-value, finance = financial difficulties

| Environment |  | tenure_1 | tenure_2 | tenure_3 | tenure_4 | tenure_5 | tenure_6 | tenure_7 | tenure_8 | tenure_9 |
| --- | --- | --- | --- | --- | --- | --- | --- | --- | --- | --- |
| Employment_1 | *r* | 0.2904 | 0.2895 | 0.2959 | 0.3017 | 0.2994 | 0.3055 | 0.3012 | 0.2992 | 0.2952 |
|  | p | 0 | 0 | 0 | 0 | 0 | 0 | 0 | 0 | 0 |
| Employment_2 | *r* | 0.2816 | 0.2798 | 0.2836 | 0.2833 | 0.2901 | 0.2892 | 0.2759 | 0.2763 | 0.2784 |
|  | p | 0 | 0 | 0 | 0 | 0 | 0 | 0 | 0 | 0 |
| Employment_3 | *r* | 0.2764 | 0.2709 | 0.268 | 0.2698 | 0.2655 | 0.2714 | 0.2693 | 0.2703 | 0.2708 |
|  | p | 0 | 0 | 0 | 0 | 0 | 0 | 0 | 0 | 0 |
| Employment_4 | *r* | 0.2731 | 0.2667 | 0.2693 | 0.2756 | 0.268 | 0.2778 | 0.2706 | 0.2693 | 0.2716 |
|  | p | 0 | 0 | 0 | 0 | 0 | 0 | 0 | 0 | 0 |
| Employment_5 | *r* | 0.249 | 0.2446 | 0.2483 | 0.2516 | 0.2572 | 0.2634 | 0.2564 | 0.2571 | 0.256 |
|  | p | 0 | 0 | 0 | 0 | 0 | 0 | 0 | 0 | 0 |
| Employment_6 | *r* | 0.2498 | 0.2408 | 0.2381 | 0.2469 | 0.2441 | 0.2507 | 0.2548 | 0.2469 | 0.2451 |
|  | p | 0 | 0 | 0 | 0 | 0 | 0 | 0 | 0 | 0 |
| Employment_7 | p | 0.2735 | 0.2565 | 0.2562 | 0.2578 | 0.2513 | 0.2647 | 0.261 | 0.2661 | 0.2718 |
|  | *r* | 0 | 0 | 0 | 0 | 0 | 0 | 0 | 0 | 0 |
| Employment_8 | p | 0.2542 | 0.2562 | 0.2566 | 0.2548 | 0.2494 | 0.2554 | 0.256 | 0.2546 | 0.2648 |
|  | p | 0 | 0 | 0 | 0 | 0 | 0 | 0 | 0 | 0 |
| Employment_9 | *r* | 0.2371 | 0.2399 | 0.2505 | 0.253 | 0.2388 | 0.2444 | 0.2384 | 0.2502 | 0.2505 |
|  | p | 0 | 0 | 0 | 0 | 0 | 0 | 0 | 0 | 0 |

Note: *r* = correlation coefficient, p= p-value

| Environment |  | z_Income_1 | z_Income_2 | z_Income_3 | z_Income_4 | z_Income_5 | z_Income_6 | z_Income_7 | z_Income_8 | z_Income_9 |
| --- | --- | --- | --- | --- | --- | --- | --- | --- | --- | --- |
| Finance_1 | *r* | -0.105 | -0.0783 | -0.0739 | -0.0814 | -0.0762 | -0.0806 | -0.0706 | -0.0511 | -0.0489 |
|  | p | 0 | 0 | 0 | 0 | 0 | 0 | 0 | 0.0017 | 0.0037 |
| Finance_2 | *r* | -0.0569 | -0.0929 | -0.0661 | -0.0752 | -0.0667 | -0.0609 | -0.069 | -0.0503 | -0.0385 |
|  | p | 0 | 0 | 0 | 0 | 0 | 0 | 0 | 0.0002 | 0.0066 |
| Finance_3 | *r* | -0.062 | -0.072 | -0.0994 | -0.0977 | -0.0873 | -0.0885 | -0.0794 | -0.0692 | -0.0684 |
|  | p | 0 | 0 | 0 | 0 | 0 | 0 | 0 | 0 | 0 |
| Finance_4 | *r* | -0.0606 | -0.0703 | -0.0783 | -0.111 | -0.0865 | -0.0825 | -0.0758 | -0.0795 | -0.0756 |
|  | p | 0 | 0 | 0 | 0 | 0 | 0 | 0 | 0 | 0 |
| Finance_5 | *r* | -0.0756 | -0.0692 | -0.0779 | -0.0927 | -0.1067 | -0.0782 | -0.0819 | -0.0798 | -0.0729 |
|  | p | 0 | 0 | 0 | 0 | 0 | 0 | 0 | 0 | 0 |
| Finance_6 | *r* | -0.0525 | -0.0523 | -0.0635 | -0.0736 | -0.0801 | -0.1077 | -0.0834 | -0.0797 | -0.0689 |
|  | p | 0.0007 | 0.0001 | 0 | 0 | 0 | 0 | 0 | 0 | 0 |
| Finance_7 | p | -0.0492 | -0.0597 | -0.0675 | -0.0818 | -0.0755 | -0.0775 | -0.1104 | -0.0914 | -0.0855 |
|  | *r* | 0.0019 | 0 | 0 | 0 | 0 | 0 | 0 | 0 | 0 |
| Finance_8 | p | -0.0484 | -0.0462 | -0.0527 | -0.0544 | -0.0506 | -0.0526 | -0.0679 | -0.0864 | -0.0671 |
|  | p | 0.0029 | 0.0008 | 0.0001 | 0.0001 | 0.0003 | 0.0002 | 0 | 0 | 0 |
| Finance_9 | *r* | -0.0518 | -0.0449 | -0.0552 | -0.0705 | -0.0593 | -0.0706 | -0.0711 | -0.0664 | -0.0872 |
|  | p | 0.0021 | 0.0016 | 0.0001 | 0 | 0 | 0 | 0 | 0 | 0 |

Note: *r* = correlation coefficient, p= p-value, finance = financial difficulties, polytomous/continuous variables were z-scored

| Environment |  | z_Income_1 | z_Income_2 | z_Income_3 | z_Income_4 | z_Income_5 | z_Income_6 | z_Income_7 | z_Income_8 | z_Income_9 |
| --- | --- | --- | --- | --- | --- | --- | --- | --- | --- | --- |
| Employment_1 | *r* | -0.379 | -0.3122 | -0.2901 | -0.2607 | -0.2399 | -0.222 | -0.2037 | -0.1879 | -0.1629 |
|  | p | 0 | 0 | 0 | 0 | 0 | 0 | 0 | 0 | 0 |
| Employment_2 | *r* | -0.303 | -0.374 | -0.3074 | -0.277 | -0.2564 | -0.2382 | -0.2199 | -0.1955 | -0.1794 |
|  | p | 0 | 0 | 0 | 0 | 0 | 0 | 0 | 0 | 0 |
| Employment_3 | *r* | -0.2685 | -0.2864 | -0.3604 | -0.2888 | -0.2706 | -0.2473 | -0.2196 | -0.2005 | -0.1935 |
|  | p | 0 | 0 | 0 | 0 | 0 | 0 | 0 | 0 | 0 |
| Employment_4 | *r* | -0.247 | -0.2574 | -0.2933 | -0.3355 | -0.2778 | -0.2561 | -0.2344 | -0.2079 | -0.1985 |
|  | p | 0 | 0 | 0 | 0 | 0 | 0 | 0 | 0 | 0 |
| Employment_5 | *r* | -0.2302 | -0.2283 | -0.2651 | -0.262 | -0.3288 | -0.2693 | -0.2362 | -0.2055 | -0.1935 |
|  | p | 0 | 0 | 0 | 0 | 0 | 0 | 0 | 0 | 0 |
| Employment_6 | *r* | -0.1962 | -0.2065 | -0.2373 | -0.2256 | -0.244 | -0.2839 | -0.2319 | -0.1981 | -0.1871 |
|  | p | 0 | 0 | 0 | 0 | 0 | 0 | 0 | 0 | 0 |
| Employment_7 | p | -0.174 | -0.1773 | -0.1944 | -0.205 | -0.2036 | -0.2176 | -0.2597 | -0.2028 | -0.1937 |
|  | *r* | 0 | 0 | 0 | 0 | 0 | 0 | 0 | 0 | 0 |
| Employment_8 | p | -0.1678 | -0.1681 | -0.1926 | -0.1979 | -0.1998 | -0.2211 | -0.2095 | -0.2524 | -0.2164 |
|  | p | 0 | 0 | 0 | 0 | 0 | 0 | 0 | 0 | 0 |
| Employment_9 | *r* | -0.1517 | -0.1472 | -0.1754 | -0.1842 | -0.1791 | -0.1872 | -0.18 | -0.196 | -0.2421 |
|  | p | 0 | 0 | 0 | 0 | 0 | 0 | 0 | 0 | 0 |

Note: *r* = correlation coefficient, p= p-value, polytomous/continuous variables were z-scored

| Environment |  | tenure_1 | tenure_2 | tenure_3 | tenure_4 | tenure_5 | tenure_6 | tenure_7 | tenure_8 | tenure_9 |
| --- | --- | --- | --- | --- | --- | --- | --- | --- | --- | --- |
| z_Rooms_1 | *r* | -0.3543 | -0.3473 | -0.3443 | -0.3395 | -0.3331 | -0.3407 | -0.3239 | -0.3133 | -0.3019 |
|  | p | 0 | 0 | 0 | 0 | 0 | 0 | 0 | 0 | 0 |
| z_Rooms_2 | *r* | -0.3502 | -0.3413 | -0.3293 | -0.3281 | -0.3183 | -0.3256 | -0.3103 | -0.3062 | -0.2949 |
|  | p | 0 | 0 | 0 | 0 | 0 | 0 | 0 | 0 | 0 |
| z_Rooms_3 | *r* | -0.351 | -0.3382 | -0.3493 | -0.3394 | -0.3269 | -0.3372 | -0.3214 | -0.3206 | -0.3124 |
|  | p | 0 | 0 | 0 | 0 | 0 | 0 | 0 | 0 | 0 |
| z_Rooms_4 | *r* | -0.3534 | -0.3379 | -0.3442 | -0.3559 | -0.3429 | -0.3532 | -0.3413 | -0.3364 | -0.326 |
|  | p | 0 | 0 | 0 | 0 | 0 | 0 | 0 | 0 | 0 |
| z_Rooms_5 | *r* | -0.3494 | -0.3269 | -0.3318 | -0.3395 | -0.3452 | -0.3554 | -0.3417 | -0.3371 | -0.321 |
|  | p | 0 | 0 | 0 | 0 | 0 | 0 | 0 | 0 | 0 |
| z_Rooms_6 | *r* | -0.3495 | -0.3294 | -0.3367 | -0.3489 | -0.3512 | -0.3679 | -0.3601 | -0.3559 | -0.3425 |
|  | p | 0 | 0 | 0 | 0 | 0 | 0 | 0 | 0 | 0 |
| z_Rooms_7 | p | -0.3406 | -0.3297 | -0.33 | -0.3411 | -0.3437 | -0.3601 | -0.3685 | -0.3613 | -0.3495 |
|  | *r* | 0 | 0 | 0 | 0 | 0 | 0 | 0 | 0 | 0 |
| z_Rooms_8 | p | -0.3471 | -0.3346 | -0.3356 | -0.3455 | -0.3458 | -0.3576 | -0.3642 | -0.3759 | -0.3604 |
|  | p | 0 | 0 | 0 | 0 | 0 | 0 | 0 | 0 | 0 |
| z_Rooms_9 | *r* | -0.3308 | -0.3229 | -0.3242 | -0.3311 | -0.329 | -0.3384 | -0.3463 | -0.3565 | -0.3672 |
|  | p | 0 | 0 | 0 | 0 | 0 | 0 | 0 | 0 | 0 |

Note: *r* = correlation coefficient, p= p-value, rooms = number of bedrooms, polytomous/continuous variables were z-scored

| Environment |  | tenure_1 | tenure_2 | tenure_3 | tenure_4 | tenure_5 | tenure_6 | tenure_7 | tenure_8 | tenure_9 |
| --- | --- | --- | --- | --- | --- | --- | --- | --- | --- | --- |
| z_SES_1 | *r* | -0.1445 | -0.1479 | -0.1629 | -0.1541 | -0.1679 | -0.1545 | -0.1658 | -0.1691 | -0.1679 |
|  | p | 0 | 0 | 0 | 0 | 0 | 0 | 0 | 0 | 0 |
| z_SES_2 | *r* | -0.1612 | -0.1724 | -0.1789 | -0.1712 | -0.185 | -0.1882 | -0.1803 | -0.1699 | -0.1753 |
|  | p | 0 | 0 | 0 | 0 | 0 | 0 | 0 | 0 | 0 |
| z_SES_3 | *r* | -0.1666 | -0.1747 | -0.1829 | -0.1798 | -0.1921 | -0.1909 | -0.1839 | -0.1793 | -0.187 |
|  | p | 0 | 0 | 0 | 0 | 0 | 0 | 0 | 0 | 0 |
| z_SES_4 | *r* | -0.1609 | -0.1635 | -0.1724 | -0.1686 | -0.1771 | -0.1852 | -0.1852 | -0.186 | -0.1948 |
|  | p | 0 | 0 | 0 | 0 | 0 | 0 | 0 | 0 | 0 |
| z_SES_5 | *r* | -0.168 | -0.164 | -0.1805 | -0.1785 | -0.1876 | -0.1906 | -0.1894 | -0.1826 | -0.189 |
|  | p | 0 | 0 | 0 | 0 | 0 | 0 | 0 | 0 | 0 |
| z_SES_6 | *r* | -0.1339 | -0.1443 | -0.1611 | -0.1531 | -0.1576 | -0.1661 | -0.1664 | -0.1585 | -0.1677 |
|  | p | 0 | 0 | 0 | 0 | 0 | 0 | 0 | 0 | 0 |
| z_SES_7 | p | -0.1355 | -0.1399 | -0.1476 | -0.1511 | -0.1536 | -0.1594 | -0.158 | -0.1584 | -0.1672 |
|  | *r* | 0 | 0 | 0 | 0 | 0 | 0 | 0 | 0 | 0 |
| z_SES_8 | p | -0.139 | -0.1256 | -0.144 | -0.1419 | -0.1469 | -0.1525 | -0.1439 | -0.1466 | -0.1627 |
|  | p | 0 | 0 | 0 | 0 | 0 | 0 | 0 | 0 | 0 |
| z_SES_9 | *r* | -0.1162 | -0.0957 | -0.1156 | -0.1121 | -0.1168 | -0.1282 | -0.1288 | -0.1222 | -0.1465 |
|  | p | 0 | 0 | 0 | 0 | 0 | 0 | 0 | 0 | 0 |

Note: *r* = correlation coefficient, p= p-value, SES = socio-economic status, polytomous/continuous variables were z-scored

| Environment |  | z_SES_1 | z_SES_2 | z_SES_3 | z_SES_4 | z_SES_5 | z_SES_6 | z_SES_7 | z_SES_8 | z_SES_9 |
| --- | --- | --- | --- | --- | --- | --- | --- | --- | --- | --- |
| Employment_1 | *r* | -0.0563 | -0.1421 | -0.16 | -0.1823 | -0.1732 | -0.1631 | -0.1946 | -0.1796 | -0.1608 |
|  | p | 0.0022 | 0 | 0 | 0 | 0 | 0 | 0 | 0 | 0 |
| Employment_2 | *r* | -0.1001 | -0.0784 | -0.1478 | -0.1805 | -0.1593 | -0.1551 | -0.1622 | -0.1508 | -0.1077 |
|  | p | 0 | 0 | 0 | 0 | 0 | 0 | 0 | 0 | 0 |
| Employment_3 | *r* | -0.0769 | -0.1007 | -0.0728 | -0.1262 | -0.1325 | -0.1272 | -0.1066 | -0.0988 | -0.0662 |
|  | p | 0 | 0 | 0 | 0 | 0 | 0 | 0 | 0 | 0.0011 |
| Employment_4 | *r* | -0.1 | -0.1201 | -0.1069 | -0.0972 | -0.1454 | -0.1158 | -0.1072 | -0.1197 | -0.0909 |
|  | p | 0 | 0 | 0 | 0 | 0 | 0 | 0 | 0 | 0 |
| Employment_5 | *r* | -0.1162 | -0.1082 | -0.0811 | -0.1014 | -0.0703 | -0.1118 | -0.0998 | -0.0727 | -0.0648 |
|  | p | 0 | 0 | 0 | 0 | 0 | 0 | 0 | 0.0002 | 0.0014 |
| Employment_6 | *r* | -0.0878 | -0.1147 | -0.0748 | -0.0773 | -0.1027 | -0.0759 | -0.065 | -0.0127 | -0.0499 |
|  | p | 0 | 0 | 0 | 0 | 0 | 0 | 0.0005 | 0.516 | 0.015 |
| Employment_7 | p | -0.1077 | -0.0958 | -0.0761 | -0.0825 | -0.0633 | -0.0481 | -0.0157 | -0.0407 | -0.0612 |
|  | *r* | 0 | 0 | 0 | 0 | 0.0005 | 0.0095 | 0.3927 | 0.0366 | 0.0028 |
| Employment_8 | p | -0.0935 | -0.0841 | -0.065 | -0.0754 | -0.0714 | -0.0508 | -0.0441 | -0.0289 | -0.0757 |
|  | p | 0 | 0 | 0.0004 | 0 | 0.0001 | 0.0072 | 0.0211 | 0.1301 | 0.0002 |
| Employment_9 | *r* | -0.0601 | -0.0662 | -0.041 | -0.0585 | -0.0708 | -0.0359 | -0.0283 | -0.0407 | -0.0156 |
|  | p | 0.0055 | 0.0003 | 0.0285 | 0.002 | 0.0002 | 0.0654 | 0.1494 | 0.0424 | 0.4369 |

Note: *r* = correlation coefficient, p= p-value, SES = socio-economic status, polytomous/continuous variables were z-scored

| Environment |  | z_Rooms_1 | z_Rooms_2 | z_Rooms_3 | z_Rooms_4 | z_Rooms_5 | z_Rooms_6 | z_Rooms_7 | z_Rooms_8 | z_Rooms_9 |
| --- | --- | --- | --- | --- | --- | --- | --- | --- | --- | --- |
| Finance_1 | *r* | -0.0871 | -0.0963 | -0.0938 | -0.1033 | -0.109 | -0.1074 | -0.1113 | -0.1002 | -0.0993 |
|  | p | 0 | 0 | 0 | 0 | 0 | 0 | 0 | 0 | 0 |
| Finance_2 | *r* | -0.057 | -0.0634 | -0.0675 | -0.086 | -0.0865 | -0.0905 | -0.0913 | -0.0953 | -0.0986 |
|  | p | 0 | 0 | 0 | 0 | 0 | 0 |  | 0 | 0 |
| Finance_3 | *r* | -0.0695 | -0.0634 | -0.0705 | -0.072 | -0.0703 | -0.0829 | -0.0825 | -0.0866 | -0.096 |
|  | p | 0 | 0 | 0 | 0 | 0 | 0 |  | 0 | 0 |
| Finance_4 | *r* | -0.0709 | -0.0607 | -0.0639 | -0.0723 | -0.0754 | -0.0885 | -0.0947 | -0.0948 | -0.0883 |
|  | p | 0 | 0 | 0 | 0 | 0 | 0 |  | 0 | 0 |
| Finance_5 | *r* | -0.0412 | -0.0474 | -0.0434 | -0.0497 | -0.0561 | -0.0623 | -0.0652 | -0.0707 | -0.0781 |
|  | p | 0.0055 | 0.0002 | 0.0006 | 0.0001 | 0 | 0 | 0 | 0 | 0 |
| Finance_6 | *r* | -0.0501 | -0.0542 | -0.0527 | -0.0596 | -0.0678 | -0.0707 | -0.0711 | -0.0755 | -0.0855 |
|  | p | 0.0012 | 0 | 0.0001 | 0 | 0 | 0 | 0 | 0 | 0 |
| Finance_7 | p | -0.0573 | -0.0652 | -0.0623 | -0.0683 | -0.0705 | -0.0744 | -0.0744 | -0.0918 | -0.1083 |
|  | *r* | 0.0003 | 0 | 0 | 0 | 0 | 0 | 0 | 0 | 0 |
| Finance_8 | p | -0.0788 | -0.0651 | -0.0656 | -0.0663 | -0.0738 | -0.0697 | -0.071 | -0.0771 | -0.0896 |
|  | p | 0 | 0 | 0 | 0 | 0 | 0 | 0 | 0 | 0 |
| Finance_9 | *r* | -0.0649 | -0.0652 | -0.0604 | -0.0684 | -0.0655 | -0.0688 | -0.0667 | -0.0687 | -0.0666 |
|  | p | 0.0001 | 0 | 0 | 0 | 0 | 0 | 0 | 0 | 0 |

Note: *r* = correlation coefficient, p= p-value, finance = financial difficulties, rooms = number of bedrooms, polytomous/continuous variables were z-scored

|  |  | z_Room~1 | z_Room~2 | z_Room~3 | z_Room~4 | z_Room~5 | z_Room~6 | z_Room~7 | z_Room~8 | z_Room~9 |
| --- | --- | --- | --- | --- | --- | --- | --- | --- | --- | --- |
| Employment_1 | *r* | -0.0282 | -0.0327 | -0.0332 | -0.0461 | -0.0489 | -0.0656 | -0.069 | -0.0643 | -0.0645 |
|  | p | 0.0402 | 0.0173 | 0.0186 | 0.0014 | 0.001 | 0 | 0 | 0.0001 | 0.0001 |
| Employment_2 | *r* | -0.0438 | -0.0279 | -0.0355 | -0.0409 | -0.0512 | -0.0534 | -0.0482 | -0.0524 | -0.0634 |
|  | p | 0.0014 | 0.0175 | 0.0031 | 0.0009 | 0 | 0 | 0.0003 | 0.0001 | 0 |
| Employment_3 | *r* | -0.0351 | -0.0138 | -0.0158 | -0.0221 | -0.0224 | -0.0174 | -0.0226 | -0.0205 | -0.0291 |
|  | p | 0.0131 | 0.2516 | 0.1841 | 0.0722 | 0.0743 | 0.1837 | 0.091 | 0.1347 | 0.0402 |
| Employment_4 | *r* | -0.0286 | -0.0207 | -0.0189 | -0.031 | -0.0226 | -0.0254 | -0.0288 | -0.03 | -0.0375 |
|  | p | 0.0482 | 0.0918 | 0.1252 | 0.0114 | 0.0727 | 0.0524 | 0.0312 | 0.0289 | 0.0084 |
| Employment_5 | *r* | -0.0353 | -0.024 | -0.0204 | -0.0305 | -0.0202 | -0.0293 | -0.0228 | -0.0261 | -0.0357 |
|  | p | 0.0169 | 0.0557 | 0.1037 | 0.0157 | 0.1075 | 0.0253 | 0.0894 | 0.0578 | 0.0123 |
| Employment_6 | *r* | -0.0419 | -0.0318 | -0.0335 | -0.0345 | -0.0255 | -0.0328 | -0.0323 | -0.0295 | -0.0363 |
|  | p | 0.0065 | 0.0149 | 0.0105 | 0.0086 | 0.0528 | 0.0121 | 0.0169 | 0.0335 | 0.0115 |
| Employment_7 | p | -0.0527 | -0.036 | -0.0377 | -0.0423 | -0.0369 | -0.0367 | -0.0363 | -0.0392 | -0.0448 |
|  | *r* | 0.0009 | 0.0071 | 0.0048 | 0.0017 | 0.0062 | 0.0069 | 0.0065 | 0.0047 | 0.0018 |
| Employment_8 | p | -0.0659 | -0.0675 | -0.0725 | -0.0706 | -0.0675 | -0.065 | -0.0706 | -0.0712 | -0.0762 |
|  | p | 0 | 0 | 0 | 0 | 0 | 0 | 0 | 0 | 0 |
| Employment_9 | *r* | -0.0438 | -0.0386 | -0.0453 | -0.0497 | -0.0489 | -0.0511 | -0.0444 | -0.0508 | -0.0464 |
|  | p | 0.0093 | 0.0065 | 0.0014 | 0.0005 | 0.0006 | 0.0004 | 0.0019 | 0.0004 | 0.0012 |

Note: *r* = correlation coefficient, p= p-value, rooms = number of bedrooms, polytomous/continuous variables were z-scored

# **References**

1. StataCorp. *Stata Statistical Software: Release 12*. College Station, TX: StataCorp LP; 2011.
